# Human-environment interaction during the Holocene in Eastern South America: Rapid climate changes and population dynamics

**DOI:** 10.1371/journal.pone.0315747

**Published:** 2025-02-03

**Authors:** Astolfo G. M. Araujo, Letícia Cristina Correa, Glauco Constantino Perez, Enrico Dalmas Di Gregorio, Mercedes Okumura

**Affiliations:** 1 Museum of Archaeology and Ethnology, University of São Paulo, São Paulo, Brazil; 2 School of Arts, Sciences, and Humanities, University of São Paulo, São Paulo, Brazil; 3 Laboratory of Archaeology, Evolutionary and Experimental Prehistory, Federal University of Rio Grande do Sul, Rio Grande do Sul, Brazil; 4 Institute of Biosciences, University of São Paulo, São Paulo, Brazil; Universidad Nacional de la Plata Facultad de Ciencias Naturales y Museo, ARGENTINA

## Abstract

About 15 years ago, we suggested that the low frequency of archaeological sites dating from the mid-Holocene in several regions of Lowland South America (which was then called the “Archaic Gap”) was due to an increase in the magnitude of dry periods related to the mid-Holocene hypsithermal. Since then, data regarding paleoenvironmental reconstructions for this vast area, coupled with an increase in the archaeological knowledge, allow us to reassess the idea of the “Archaic Gap” and redefine both the spatial extent of the phenomenon and its possible causes. Our present analysis aims to present a broader picture of the relations between humans and the environment in Eastern South America since the Late Pleistocene. The obtained results suggest that the extent of the areas that were somewhat depopulated during the mid-Holocene is larger than previously thought; not only Central Brazil, but parts of the Amazon and the Pantanal (close to the Bolivian border) seem to show the same pattern. However, as expected when larger datasets are available, it is possible to perceive oscillations in the archaeological signal that suggest reoccupation of some areas. Although we maintain that the main reasons underlying these patterns are related to climate, they are most probably related to an increase in climatic variability, and not necessarily to an increase in dryness. These observations are of interest to the current debate about the effects of the global warming on human populations.

## Introduction

The Holocene is a time period in the Earth’s history that can be considered mild in climatic terms when compared to the Pleistocene [1–3], and these benign conditions played a key role in several aspects of the history of our species, from the colonization and/or recolonization of new territories [4] to the establishment of agriculture as a viable system of food acquisition [5]. However, even if less drastic, climatic shifts during the Holocene were not uncommon, and were called “rapid climate changes”, or RCCs [6]. The definition of RCCs is “changes [which] are sufficiently fast from the point of view of human civilization (i.e., a few hundred years and shorter)” that they may be considered ‘rapid’” ([6]:245). Since then, an increasing number of authors started to pay attention to such events, as can be seen on Table 1.

**Table 1:** Main RCC events, age ranges and references.

RCC label	Age range	Authors
Meltwater pulse 1A	14.9 to 13.9 ka	[8]
14.8 ka climate shifts	Ca. 14.8 ka	[39, 40, 260]
Lake Agassiz Herman outburst	ca. 12.9 ka	[9]
Lake Agassiz Norcross outburst	ca. 11.7 ka	[9]
11.4 ka warm excursion	ca. 11.4 ka	[250]
Lake Agassiz Tintah outburst	ca. 11.2 ka	[9]
Lake Agassiz Upper Campbell outburst	ca. 10.6	[9]
Meltwater pulse 1C	9.8 ka	[8]
9 to 8 ka Glacial Aftermath	8.8 to 8.0 ka	[6]
9.2 ka event	9.5 to 9.2 ka	[38, 178]
8.2 ka event	ca. 8.2 ka	[93, 261]
7.6 ka event	7.6 to 7.4 ka	[8, 250]
7.2 ka event	7.6 to 7.0 ka	[41]
6.4 ka event	ca. 6.4 ka	[97]
5.5 ka event	ca. 5.5 ka	[95]
5.3 ka event	ca. 5.3 ka	[97]
4.8 warm excursion	ca. 4.8 ka	[250]
4.2 ka event	4.2 to 4.0 ka	[91, 250]
3.6 ka event	3.6 to 3.4 ka	[12]
3.0 to 2.3 ka event	3.0 to 2.3 ka	[96]
2.8 ka event	2.8 to 2.71 ka	[94]
2.1 ka event	2.3 to 2.0 ka	[12]
Roman Warm Period (RWP)	2.0 to 1.3 ka	[179]
1.6 ka cold excursion	ca. 1.6 ka	[250]
Late Antique Little Ice Age (LALIA)	1.6 to 1.3 ka	[179]
1.4 ka event	ca. 1.4 ka	[97]
1.0 ka warm excursion	ca. 1.0 ka	[250]
Little Ice Age (LIA)	0.75 to 0.1 ka	[262]

Factors that lead to a RCC are many, probably a conjunction of 1) solar variability superimposed on changes in insolation [6]; 2) orbitally driven cycles of insolation [7]; 3) meltwater pulses [8]; 4) major glacial lakes collapse [9]; 5) changes in the ocean circulation patterns [10]; 6) increase in volcanic activity [11, 12]. In spite of the recognition that climatic shifts can have a major impact on human populations [13, 14], this tends to be regarded mostly as a phenomenon that affects either large-scale agriculturalists [15–17] or hunter-gatherers and pastoralists living in marginal environments [18–21]. However, previous research [22, 23] suggested that climate could affect hunter-gatherers living in much milder environmental conditions in a sensible way, such as the case of Lowland South American tropical settings. A few years later, based on several paleoenvironmental and geomorphologic studies, we proposed that prehistoric human populations in Eastern South America were most probably impacted by extremely variable climatic conditions prevailing in specific periods during the Holocene, rather than by prolonged dry / wet periods [24]. This idea was resumed by Riris and Arroyo-Kalin [25] which suggested the same pattern could be extrapolated to South America as a whole. As a natural next step, it is now necessary to explore the available data in a more detailed way and check how the variable climate model can be applied in wider areas.

### Geographic and environmental characteristics of the area

**Fig 1:**
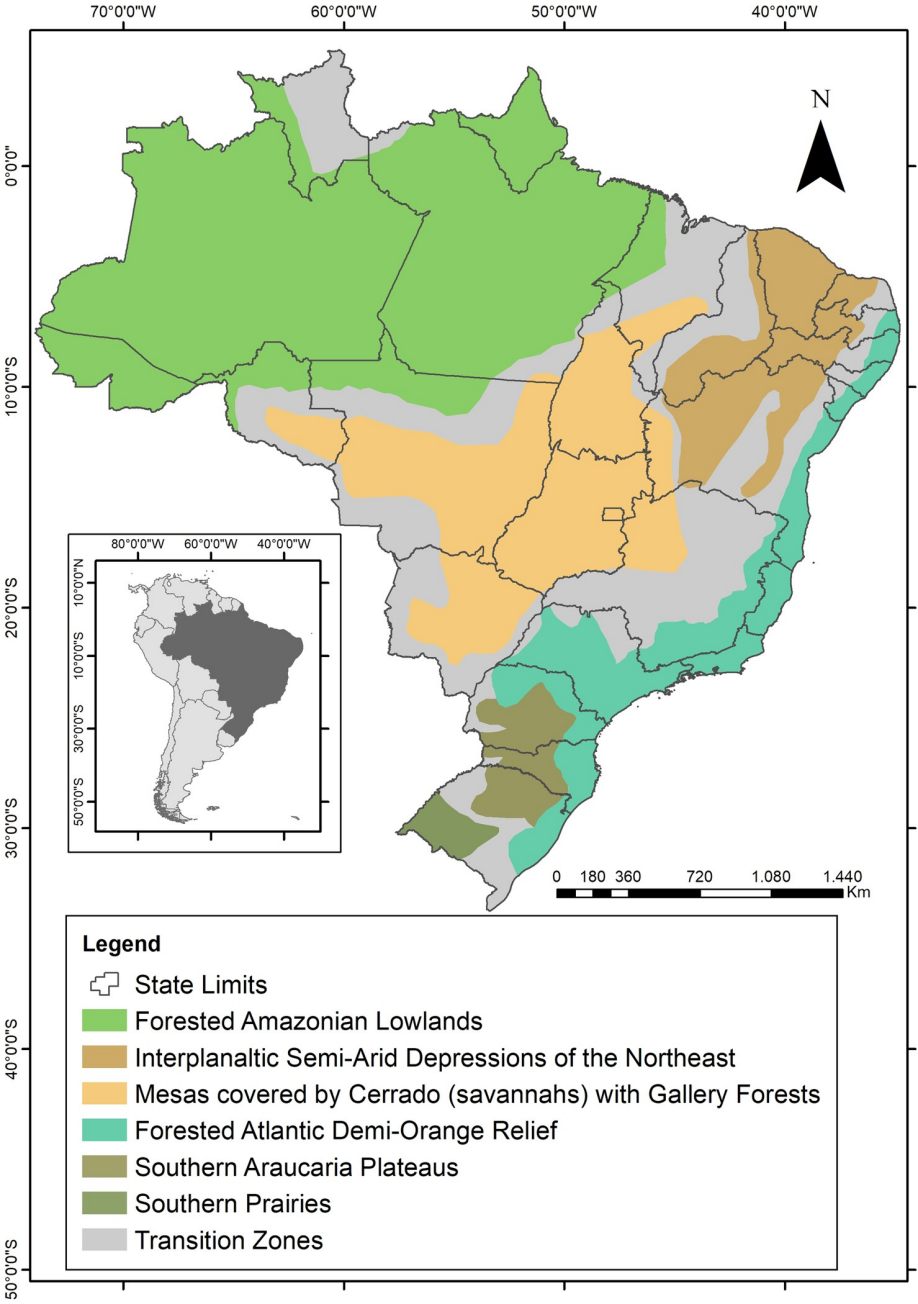
Environmental domains in Eastern South America, after Ab’Sáber (2007).

From a geomorphological point of view, the eastern portion of South America is traditionally divided, from North to South, into the Guiana Highlands, the Amazon Basin, the Brazilian Highlands, and a very large plain that stretches from the center of the continent towards south, reaching the River Plate estuary, called Pantanal / Gran Chaco / Paraguay-Parana Basin. Of course, this small scale relief classification simplifies a mosaic of features that can be recognized in larger scales, including the human one. The Brazilian Highlands label, for instance, encompasses a huge diversity of landforms and climates, from the semi-arid to the pluvial, from extensive plateaus to high mountain ranges. In this article we will take into account the major characteristics of the biomes and relief in order to organize our text and convey the basics of the Eastern South American environmental / landscape variability. Therefore, we will establish the following environmental domains, partially based on Ab’Sáber [26] landscape domains: a) Forested Amazonian Lowlands; b) Interplanaltic Semi-Arid Depressions of the Northeast; c) Forested Atlantic Demi-Orange Relief; d) Mesas covered by Cerrado (savannahs) with Gallery Forests; e) Pantanal Basin; f) Southern Araucaria Plateaus; and g) Southern Prairies (Fig 1).

Long-term climate patterns affecting Eastern South America (ESA) seem to be predominantly related to the intensity of summer solar radiation received by the planet, following Earth’s precession cycles [27]. When we examine the climate patterns of the Southern Hemisphere, given the complex interplay of climate forcings, there is also evidence of east-west anti-phasing. For instance, the paleo precipitation in NE Brazil seems to follow a reversed pattern when compared to the rest of South America [28]. These differences in a continental scale are composed by a mosaic of differences on more local scales, as we will see in the several examples presented. This means that, in spite of general paleoenvironmental trends, local signals can be at variance with the overall picture, and we assume that the archaeological data can be sensitive to these local signals.

### Theoretical underpinnings

Here we will address the question of the variability in the archaeological signal during the Holocene in different regions that is perceived throughout lowland South America, more specifically in its Eastern portion, or what is today Brazilian territory. The main assumption underlying the argument is that there is a relationship between the number of archaeological sites dating from a given period and the size of the population that produced them [29, 30]. Recent research in areas with very refined archaeological and chronological data suggested that this assumption is warranted [31, 32]. The age frequency curves obtained (“summed probability distribution” of the ages, or SPD [33]) can be analyzed against a background of paleoenvironmental data, and possible correlations can be sought (c.f. [34]).

A second main assumption of this study is that Holocene rapid climatic changes (“RCCs”, following Mayewski et al. [6]) are more relevant in terms of understanding the impact of climate on living beings (including humans) than extended periods of dry or humid conditions. Crossing data at a global scale, Mayewski et al., [6] proposed at least six RCCs placed between ca. 8.9 to 8.0 ka BP, 6.0 to 5.3 ka BP, 4.2 to 3.8 Ka BP, 3.3 to 2.5 ka BP, 1.4 to 1.0 ka BP, and the last 600 years. The first RCC encompasses the 8.2 ka event, a short cooling event triggered by a burst of fresh meltwater in the North Atlantic, and whose signal is recognized globally [35, 36]. The third RCC was probably triggered after the 4.2 ka event, a megadrought recorded in Middle East and Eurasia with an extreme impact on coral reefs in the Pacific, but whose mechanism is still nuclear [37]. In recent years, several other RCCs were detected around the globe (e.g., [38–42]). Not all RCCs will have the same impact at the same location, but their signals in the overall picture will be clear as we proceed with our analysis.

From the geological and geomorphological points of view, we find also important to underline the role that RCCs play on tropical landscapes [24]. We must acknowledge the important contribution that came from the work of Henri Erhart [43], who proposed the “biorhesistatic theory”, in which a given landscape with stable vegetal cover (or in “biostasy”) will be subject mainly to chemical leaching, releasing soluble minerals and retaining insoluble ones. When there is a disruption in climate, however, the biological equilibrium is lost (“rhesistasy”), vegetation retracts and the clastic and clayey insoluble component that was stored under it is released, causing valley infilling and deposition of several meters of sediment in short intervals. A parallel development of Erhart’s biorhesistatic theory was proposed by Knox [44], who devised a model of biogeomorphic response to abrupt climate changes. Despite being originally applied to the Eastern United States, the model was used by Roberts and Barker [45] in tropical Africa, and by Thomas [46, 47] in interpreting the signal of climatic changes in several tropical settings. The model predicts that an increase in precipitation causes an increase in relative vegetation cover, and therefore a decrease in the potential for hillslope erosion, and vice-versa. However, the transitions from humid to dry and from dry to humid are asymmetrical in relation to a fourth variable, namely the relative geomorphic work, or sediment yield. In normal conditions, the sediment yield in vegetated areas is lower than in semi-arid areas, due to the lack of soil protection against torrential rains in the latter. The transition from a dry to humid period will produce a peak in sediment yield, because the soil is unprotected, and rainfall would be high. This situation will last until the vegetation adjusts to the new conditions. The opposite situation, from humid to dry, tends to produce a depression in the sediment yield, since the soil is covered, and the precipitation is low. Again, this situation will last until the new vegetation (or lack thereof) prevails, and the normally high sediment yield of semi-arid settings is established. This reasoning is relevant in order to understand that sometimes the evidence of rapid climate changes is being signalled in the input of siliciclastic materials, and not in the regularly used proxies. The same reasoning goes for fire events in the Cerrado vegetation, that tend to be more frequent when rapid climatic shifts put the vegetation under stress. In fact, RCCs can explain several instances of disagreeing proxies, since the time averaging inherent in lake records, for instance, will eventually condense several decades of dry-wet episodes into a single “mid-term” episode.

In sum, any interdisciplinary research must deal with the convergence of multiple lines of evidence [48–52], and proxies which do not converge have to be dealt with accordingly. We believe that there is no fixed hierarchy for any kind of data or proxy, each one contributing in a different way to account for a given past scenario.

A more in depth discussion about the problems which can interfere in the proposed relationship between ages, populations, and paleoenvironments, as well which kind of information can be extracted from SPDs, can be found in S1 File.

## Methods ‐ The updated paleoenvironmental and archaeological database

**Fig 2:**
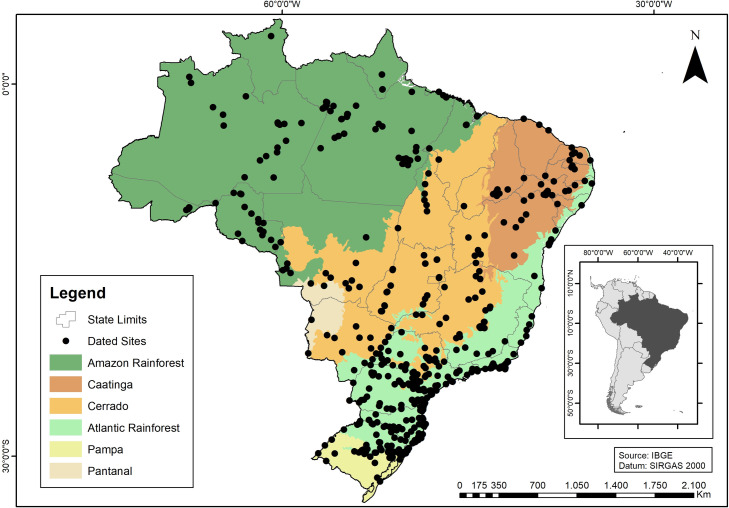
Spatial distribution of dated sites and main extant biomes.

**Fig 3:**
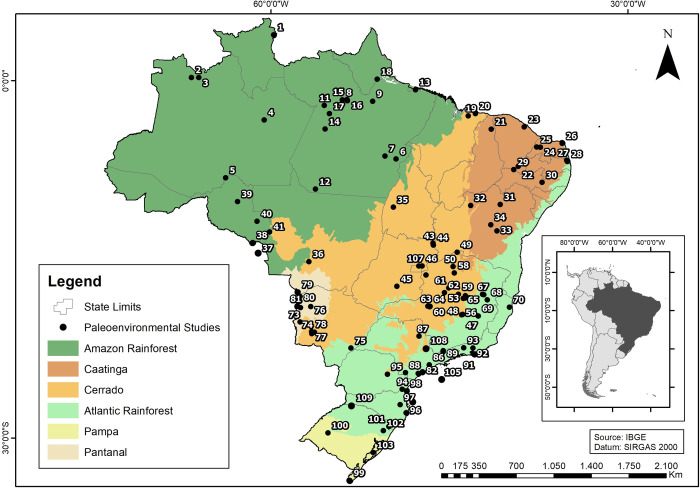
Extant biomes and paleoenvironmental studies in Eastern South America. Biomes: Amazonian Rainforest; Caatinga (xeric schrublands); Cerrado (savannahs); Atlantic Rainforest; Pampa (grasslands), and Pantanal (seasonal wetlands). Paleoenvironmental studies: 1)Caracanã Lake; 2) Hill Six Lakes; 3) Pata Lake; 4) Calado Lake; 5) Campos Humaitá; 6 and 7) Carajás; 8) Comprido Lake; 9) Curuá river; 10) Geral Lake; 11) Curuaí Lake; 12) Saci Lake; 13) Curuçá Lake; 14) Paraiso Cave;15) Santa Maria Lake; 16) Saracuri Lake; 17) Tapajós Lake; 18) Tapera Lake; 19) Caçó Lake; 20) Barreirinhas; 21) Sete Cidades; 22) Araripe soils 1; 23) Serra Maranguape; 24) Rio Grande do Norte caves; 25) Chapada do Apodi; 26) Boqueirão Lake; 27 and 28) Guaribas; 29) Arararipe soils 2; 30) Catimbau; 31) Salitre travertines; 32) Icatu dunes; 33) Paixão Cave; 34) Diva de Maura Cave; 35) Confusão Lake; 36) Pau D´Alho Cave; 37) Chaplin Lake (Bolivia); 38) Bella Vista Lake (Bolivia); 39) Ariquemes soils; 40) Pimenta Bueno soils; 41) Vilhena soils; 42) São Bernardo Cave; 43) Angelica Cave; 44)São Mateus Cave; 45) Cromínia swamp; 46) Feia Lake; 47) Olhos Lake; 48) Lagoa Santa; 49) Lapa Grande Cave; 50) Pandeiros Swamp; 51) Pinheiro swamp; 52) Pau de Fruta swamp; 53) Serra da Doida peat bog; 54) Rio Preto swamp; 55) Machado soils; 56) Mares Lake; 57) Tamboril Cave; 58) Lapa sem Fim Cave; 59) Juquinha swamp; 60) Salitre Lake; 61) São José swamp; 62) Laçador swamp; 63) Serra Negra Lake; 64) Salitre soils; 65) Nova Lake; 66) Aleixo Lake; 67) Pires Lake; 68) Água Preta Lake; 69) Dom Helvécio Lake; 70) Sooretama; 71) Negra Lake; 72) Castelo Lake; 73) Nabileque; 74) João Arruda Cave; 75) Taquaraçu; 76) Nhecolândia; 77) Bodoquena; 78) Jaraguá Cave; 79) Gaiba Lake (Bolivia); 80) Mandioré Lake (Bolivia); 81) Cáceres Lake; 82) Juréia; 83) Cananéia; 84) Serra da Bocaina; 85) Campos do Jordão; 86) Colônia; 87) Tamanduá river; 88) Santana Cave; 89) Morro de Itapeva; 90) Cabo Frio; 91) Maricá; 92) Serra dos Órgãos; 93) Paraíba do Sul; 94) Serra de Araçatuba; 95) Serra Campos Gerais; 96) Palhoça; 97) Botuverá Cave; 98) Volta Velha; 99) Mirim Lake; 100) São Francisco de Assis; 101) Cambará do Sul; 102) São Francisco de Paula; 103) Patos Lagoon; 104) Ocean Core 7606; 105) Ocean Core 7616; 106) Ocean Core 7620; 107) Águas Emendadas; 108) Mogi Guaçu river; 109) Mina Modelo pond.

The database presented here is an expansion of what we already explored in our earlier papers [22, 23]. In the last decade there was an increase in the chronological database for archaeological sites; for Central Brazil, from the original 488 to 1042 ages (a 114% increase), and in Southern Brazil from 306 to 816 ages (a 170% increase). In addition, we are presenting ages for the coastal area (n = 962), the Amazon region (n = 749), and NE Brazil (n = 518). Most of these ages were published in reports or publications of limited access to an international audience. It is also important to mention that more than 60 paleoenvironmental studies covering Eastern South America have been published since 2005. Fig 2 shows the spatial distribution of dated sites and Fig 3 shows paleoenvironmental studies and extant biomes.

The archaeological database is composed mainly of radiocarbon ages, in which means they were subject to calibration using the CalPal program [53], version 2020.11. The complete archaeological database used here can be accessed as Supporting Information (DOI: 10.5281/zenodo.7637553). All ages in this paper were calibrated using the INTCAL2020 curve.

A more detailed account about the database construction and the methods used to compare age datasets can be consulted in S2 File.

## Results

**Fig 4:**
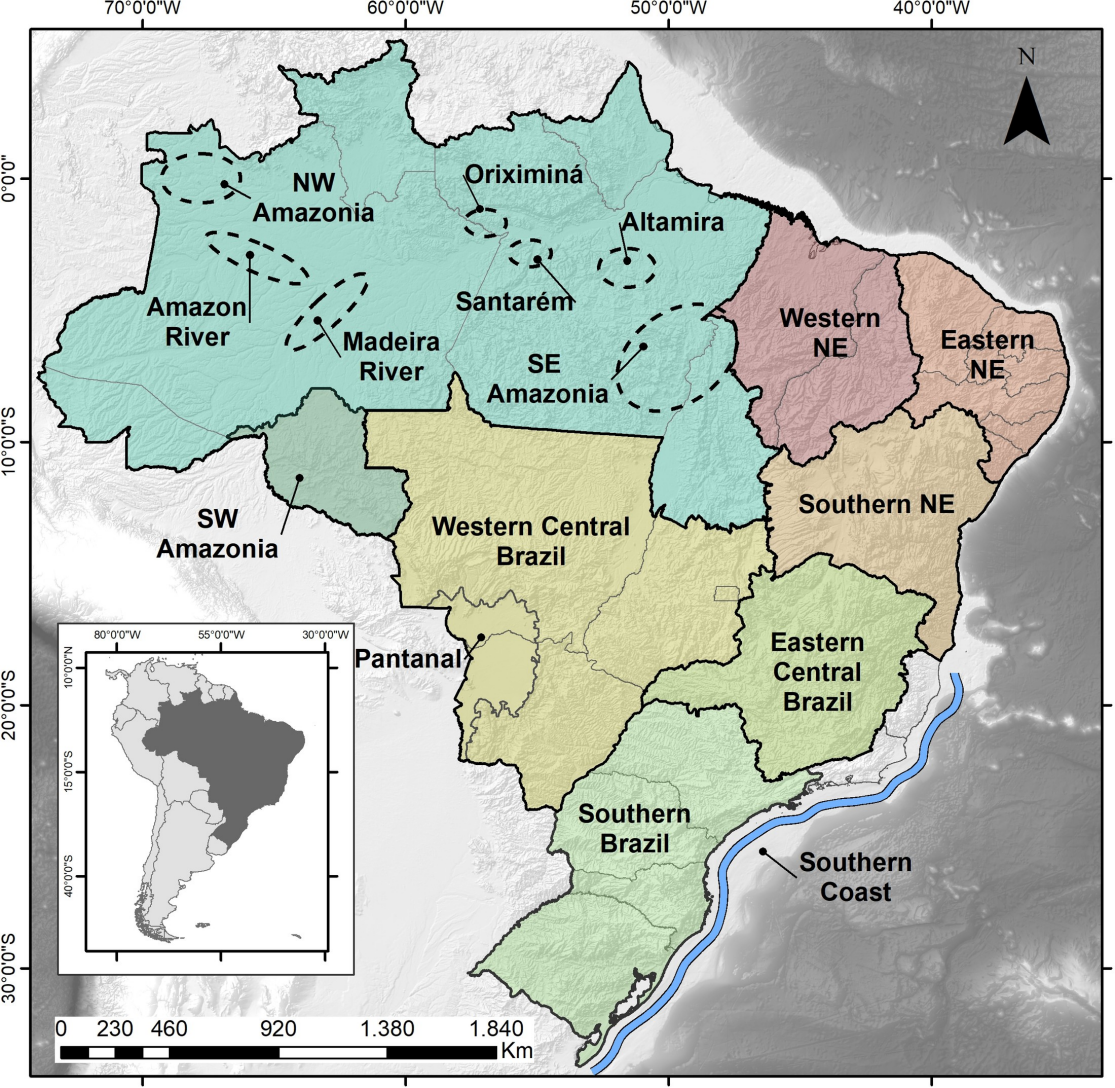
Eastern South America divided into areas as discussed in the text.

In order to enhance our understanding of the possible role that paleoclimates played in the dynamics of prehistoric human occupation of Eastern South America, we will expand the original focus based on Central, Eastern and Southern Brazil [22, 23], to comprise the Amazonian Lowlands and Northeastern Brazil, since recent data point to the existence of age patterns that need to be explored. At the same time, whenever possible we will try to establish a better spatial resolution aiming to track possible populational movements across time and space. Fig 4 shows the subdivisions of ESA we used in order to organize the data.

### The forested Amazonian Lowlands

The vast area which comprises the Amazonian Lowlands can be summarized under a single label of “tropical forest”, with a mean annual precipitation of 2300 mm [54], but some areas show much less precipitation, for instance across the “Amazonian Dry Corridor”, a NW-SE zone with mean annual precipitation below 1750 mm extending from Venezuela to Central Brazil (e.g., [55, 56]), and therefore the region harbors a high diversity of phytophysiognomies, ranging from forests to savannahs. This characteristic is related not only to soils and topography, where well drained, higher grounds support *terra firme* forests, whereas seasonally inundated lower terrains present *varzea/igapó* vegetation. Prehistoric human impact on the vegetation history of this region is contentious, and despite claims about profound human induced modifications (e.g. [57]), there is ground to acknowledge that such an impact can be meaningful but, at the same time, very localized [58–60].

#### Paleoenvironments

The role of paleoenvironments is obviously important to understand the extant Amazonian region, and we can invoke the Last Glacial Maximum (LGM), around 23 to 19 ka [61], when climate was much colder than today, as well as the middle Holocene dry phases [62] as important climatic events that impacted the region in the last millennia. In this regard, paleoenvironmental studies for the Amazonian Lowlands reached a consensus about a decrease in temperature during the LGM, some consensus about the decrease in the precipitation, and no consensus about the degree of forest fragmentation leading to the establishment of savannahs or forest refugia ([63–73]). A more comprehensive discussion about the paleoenvironmetal data for this region is provided in the S3 File.

#### Archaeological data

**Fig 5:**
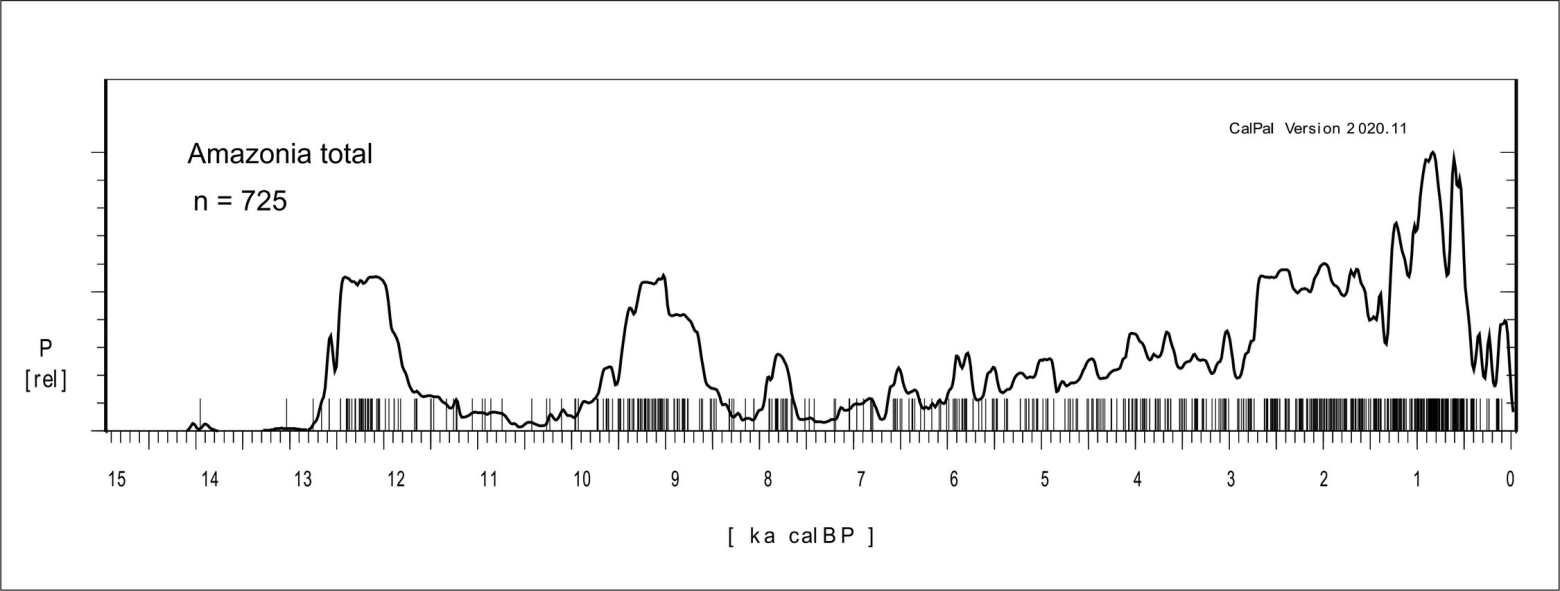
Summed probability distribution graph of 725 ages for Amazonia.

The summed probability distribution (SPD) graph for whole Amazonian database, comprising 725 ages, is presented in Fig 5. The multimodal character of the curve is readily appreciated, with a conspicuous cluster of ages bracketed between 12.8 and 11.7 ka BP and a second cluster between 10.0 and 8.3 ka BP. After 8.3 ka BP the distribution of ages suggests lower population levels, with minima between 7.6 and 6.0 ka BP, only reaching values comparable to the early Holocene at 2.8 ka, when population increases sharply and is maintained until 1.7 ka BP. After that, there seems to be a a depression around 1.3 ka BP followed by a sharp increase, with population reaching its historical maximum between 0.9 and 0.8 ka BP. It is interesting to note that after this major peak, there is apparently a sharp decrease in ages 100 years later, around 0.7 ka BP, 200 years before the first European contact. From this initial and admittedly rough picture it is possible to perceive that the Mid-Holocene low lake stands recognized between 7.0 and 6.0 ka BP in the paleoenvironmental literature are coincident with the lowest human occupation signal in Amazonia. In fact, it is possible to use the archaeological data as a proxy itself and suggest that the droughts started at 7.6 ka BP, and not at 7.0 ka BP.

The next step was to compare sets of ages which are geographically close and, whenever possible, discuss them based on specific paleoenvironmental studies.

**Fig 6:**
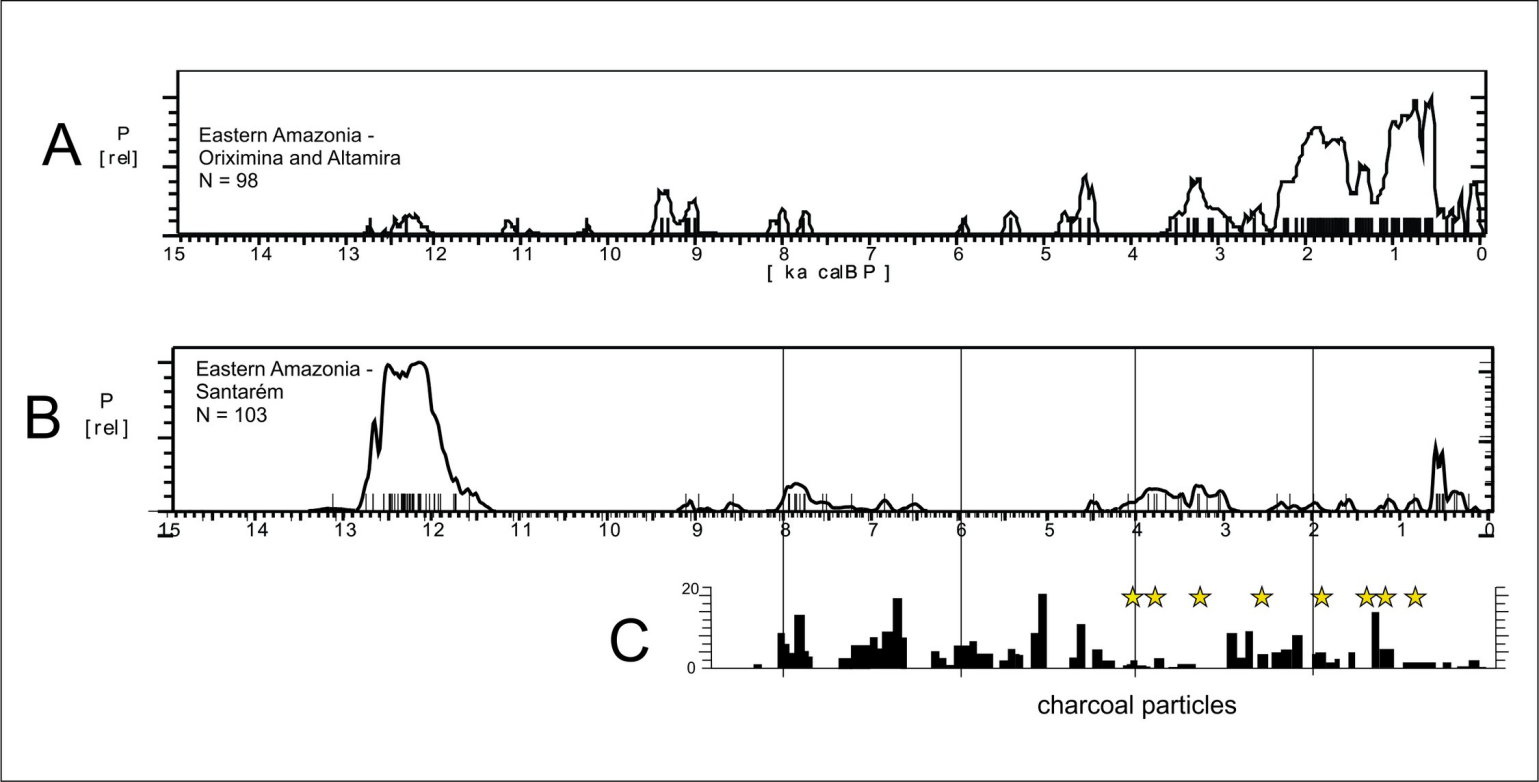
Summed probability distribution graphs for A) Altamira / Oriximiná and B) Santarém. The lower bar graph (C) was modified from Bush et al. 2007 and shows charcoal particles recovered at Geral lake. The stars mark the presence of maize pollen (Zea mays).

*Eastern Amazonia*. This region can be divided into three main areas or clusters of dated archaeological sites (Fig 4): From East to West, Altamira (n = 44), Santarém (n = 103), and Oriximiná (n = 53). Initially we ran a Kruskall-Wallis test to see if there were significant differences between the age distributions, and the results pointed towards the similarity of Altamira and Oriximiná (H = 89.4532, 2 df, p = 0.847), but not Santarém (p < 0.0001). It is important to note that Santarém comprises Pedra Pintada rockshelter [74], an Early Holocene site intensively dated, which could impart a bias in the age distribution for the Santarém area. We made a test running the analysis after excluding Pedra Pintada, but the results were the same. Therefore, we can safely assume that Santarém shows a different pattern in comparison to Altamira (280 km eastwards) and Oriximiná (200 km westwards). Fig 6 shows SPD graphs for Santarém and Oriximiná / Altamira. The early Holocene shows a strong signal of human occupation at Santarém (again, biased by Pedra Pintada) also perceived in the East (Paquiçamba sites) but not in the West. The difference can be related to the fact that Pedra Pintada is a rockshelter, where human occupation is concentrated, whereas in other portions of Amazonia this same Paleoamerican horizon would be subject to the strong influence of the fluvial geomorphic processes as well as to the prevalent academic bias on later periods. Santarém shows a total absence of ages between 11.5 and 9 ka BP, and again between 6.5 and 4.5 ka BP, while the other areas, which are located both upriver and downriver, show signs of human occupation. The Curuá river record [75], located 180 km NE of Altamira, shows a strong increase in charcoal (human presence) at ca. 2.6 ka BP, including manioc (*Manihot esculenta*) pollen, what is in accordance with the age pattern for the region (Fig 6A).

There are several paleoenvironmental studies in the Santarém region: Geral lake, Saracuri lake, Santa Maria lake (Fig 3, number 10, 15, 16; [58]), Comprido lake (Fig 3, number 8; [76]), Tapajós lake (Fig 3, number 17; [77]) and Paraíso cave (Fig 3, number 14; [78]). While all the lake records point consistently towards drier climates in the mid-Holocene, the Paraíso speleothems show the opposite trend. As previously mentioned, the archaeological record (Fig 6) shows a clear, major hiatus in the Santarém area between 6.5 and 4.5 ka BP, which tends to support the lake records. Bush et al. [79] discussed at length the possible human impacts on the three lake records: at Geral lake (~ 12 km from the Amazon river and 50 km from Pedra Pintada) the record of pollen and charcoal suggests human impact on the lake vicinities. However, when we compare the graph of charcoal particles (indicative of vegetation burn) provided by Bush et al. ([79]:214; Fig 6C) and the age distribution (Fig 6B), it becomes clear that the mid-Holocene charcoal peak (ca. 5 ka BP) is not coincident with an increase in human activity. On the contrary, it falls inside the age hiatus. Similar charcoal peaks around 5 ka BP were found in the nearby Santa Maria and Saracuri lakes. On the other hand, pollen signalling the onset of horticultural practices (*Zea mays*) starts to appear around 4 ka BP at Geral lake, this time coincident with the reoccupation of the Santarém region. In sum, Bush et al. [79] were rightly cautious in acknowledging the possible human origin of charcoal particles, but our data suggests that the charcoal produced in the mid-Holocene was most probably related to natural factors.

The Tapajós lake record [77] is related to the Tapajós river itself, and not to a closed lake, and as the authors acknowledge, it would present “a more muted paleoecological signal than a small closed lake in an ecotonal setting” ([77]:530). The record starts ca. 10 ka BP (not clear if the four radiocarbon ages across the 42 m core were determined in organic matter or charcoal) and there are two paleoenvironmental shifts detected at ca 8.8 ka BP (high siderite input suggesting a very low water stand) and an increase in *Cecropia*, a pioneer plant related to open forest canopy, between 9.2 and 4.6 cal BP. Unfortunately, the small number of radiocarbon ages and the inderminacy of the dated materials impart problems with the match between paleoenvironmental and archaeological data. It is possible that the “8.8 ka BP” low lake stand is actually related to the gap between 8.6 and 8.2 ka BP, and that the “9.2 to 4.6 ka BP” period of forest disturbance is actually related to the observed gap in ages between 6.4 and 4.6 ka BP. In the specific case of Tapajós lake, its position along a major river and geographical proximity to several archaeological sites (e.g., [80]) can produce a mixed environment / human signal and, as acknowledged by the authors, “a change in the intensity of human land use as early as 4300 cal years BP is not unlikely in this setting ([77]:531). This last remark is in absolute agreement with the archaeological signal. The Comprido lake record, located 90 km East of Santarém and at the opposite margin of the Amazon river in relation to Pedra Pintada rockshelter, showed signals of dry climate between 10.3 and 7.8 ka BP (low TOC values), and a “gap sedimentation due to a complete dryness of the lake” between 7.8 and 3.0 ka BP ([76]: 55), which seems in good accordance with the SPD curve (Fig 6B).

Paraíso cave is located 200 km SW of Santarém. There are 13 ages for the surrounding area (Itaituba and Rurópolis municipalities), and none falls in the interval between 6.4 and 3.0 ka BP. However, we believe that the weakening of the archaeological signal is not necessarily related to increases or decreases in precipitation, but to climatic variability, as we will explore below. The archaeological signal is very weak during the mid-Holocene, which at first glance could favour the dryness scenario. However, we believe the archaeological data points to a third possibility, which actually explains both trends observed in lakes and in the speleothems: the mid-Holocene was climatically *very unstable*. The conflicting paleoenvironmental scenarios are due to different proxies and their different sensitivity to environmental variables. The archaeological data in this regard should be understood as a different proxy (a point we already made in [22]). The lowering of the archaeological signal reflects the instability of the climatic pattern, and especially before the spread of agricultural practices, mobility was probably a very effective way to cope with unpredictable resources. To make our point clearer, we ran a series of analyses using the available data for Paraiso cave (see supplementary material in [78]). Our alternative analysis took into consideration two parameters: 1) the rates of speleothem growth and 2) the *variation* in the time series of isotopic data.

**Fig 7:**
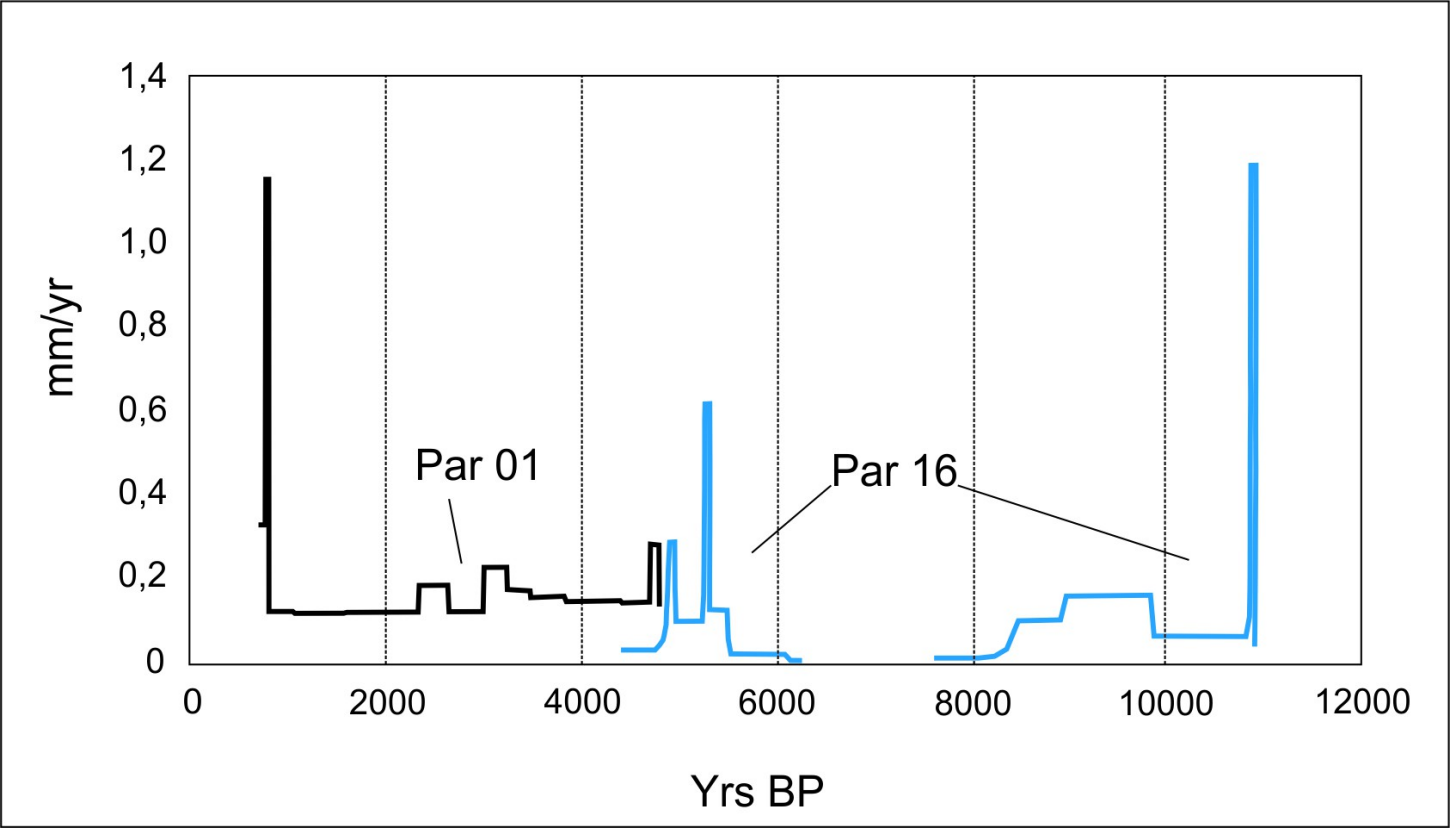
Paraiso cave speleothem growth rates. Speleothem “Par 16” shows a hiatus between ca. 8 and 6 ka BP.

Rates of speleothem growth can be considered sensitive to several factors, but our reasoning is that their variation is indicative of some kind of disturbance or change in some set of environmental variables. This is a very simple yet enlightening perspective, since we can easily see steps in the variation of the speleothem growth rates combining speleothems PAR 1 and PAR 16, which cover a time interval between 0.7 and 11 ka BP (Fig 7). Moreover, the very existence of large periods of stable speleothem growth observed as plateaus in Fig 7 suggest that these rates are far from random and, therefore, significant for our purposes. The visual inspection of Fig 7 shows a peak around 11 ka BP followed by a relatively slow growth rate, followed by steps around 9 and 8 ka BP. Between 8 and 6 ka BP the speleothem was inactive and the growth resumes after 6 ka BP. Most importantly, in the period between 6 and 4 ka BP the rates show a very significant fluctuation and speleothem PAR 16 stops growing again. These hiatuses in speleothem growth can be related to very localized water paths inside the rock and not necessarily informative of the signal of climate change (if towards dryness or not) but at least they are informative of some change, since there is some overlap with speleothem PAR 1, that resumed growth during the fluctuation period.

**Fig 8:**
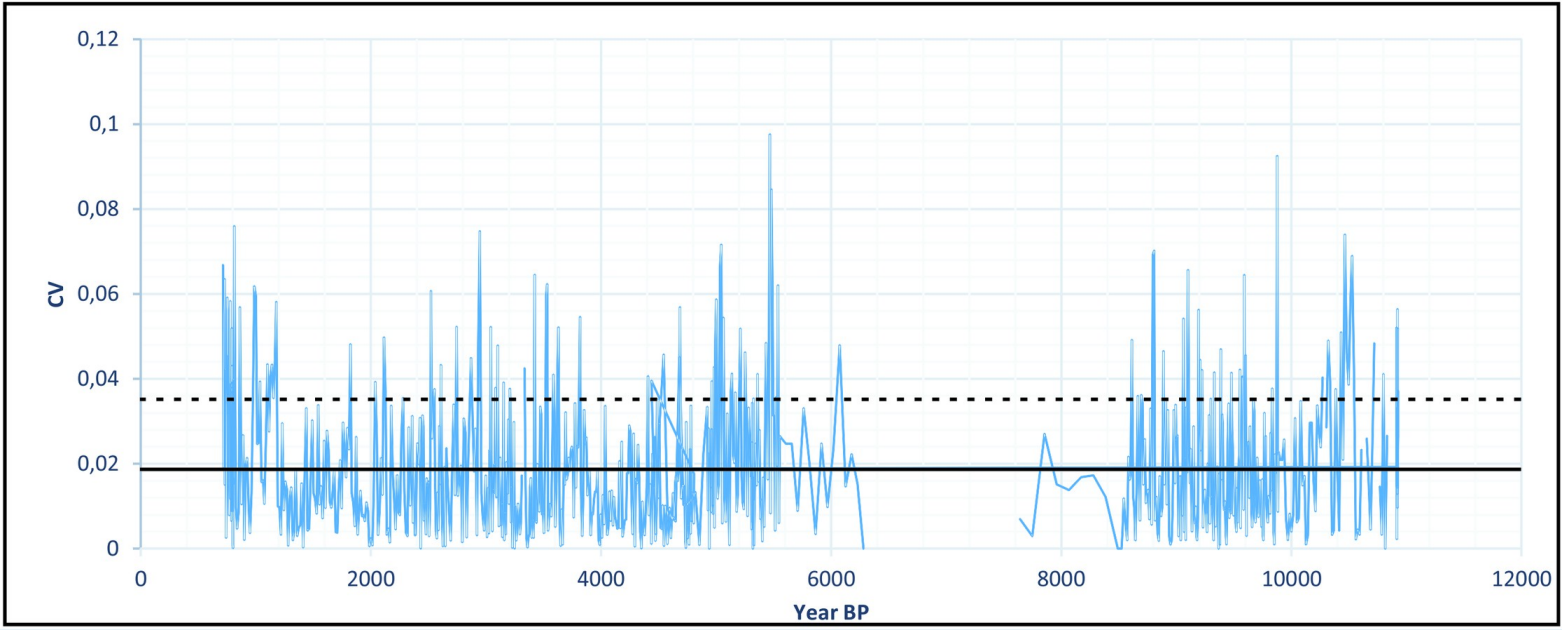
Coefficient of variation for the delta ^18^O isotopic sequence from Paraiso cave in the last 11 ka BP. The horizontal solid line represents the mean value (0,019) and the dashed line the mean plus one standard deviation (0,035). We considered values above the dashed line to represent extreme fluctuations.

As a first approach, the growth rates can help us see a broader picture. However, in order to better understand what degree of variation we are dealing with, we ran the coefficient of variation (CV) for each two adjacent measurements of delta ^18^O in the PAR1 and PAR16 speleothems. This provided a measure of isotopic variation inside a time slice with resolution between 4 and 30 years, which is well related to a human perspective. Fig 8 shows the obtained graph where very high variation peaks appear throughout the sequence.

**Fig 9:**
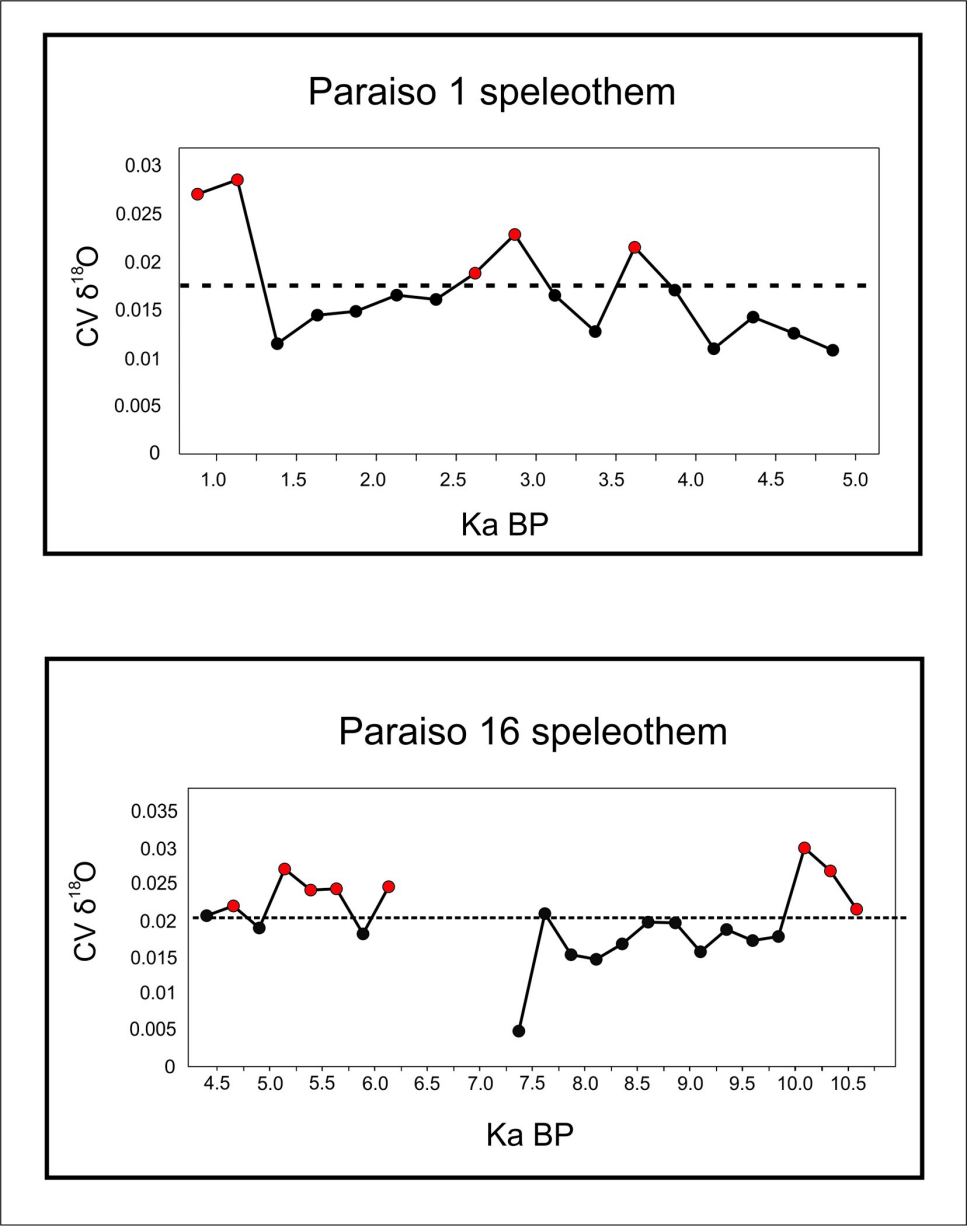
Mean of delta ^18^O CVs of 250-year time intervals for Paraiso 1(PAR 1) and Paraiso 16 (PAR 16) speleothems. The dashed horizontal line represents the value of the mean plus one standard deviation. Red dots represent time intervals where the variation was considered extreme.

If we take the mean CV (0,019) and the standard deviation (0,016), values above one standard deviation (0,035) could be considered as representing extreme variations. In order to have a better idea about which periods could be considered more variable across the Holocene, we ran a new analysis taking the mean of the CVs in 250 years’ time slots. The results are shown in Fig 9.

It is possible to observe that there was a period of extreme variation in the early Holocene (between 11 and 10 ka BP) followed by benign climatic conditions until perhaps 6 ka BP. After that, we have several centuries of extreme climatic variation up to 4.2 ka BP. Therefore, seen from this perspective, the results from Paraiso speleothems tend to show the reason for the pollen assemblage present at the lakes: extreme climatic variability, rather than long periods of drought. It is worth mentioning that several drought events intercalated with high lake stands can be responsible for major vegetation shifts and the colonization of margins by pioneer species. On the other hand, the isotopic signal of Paraiso cave can indeed point towards more rain, but on *average*.

When we compare Figs 9 and 6C, it becomes clear how well the results match: the PAR16 data start at 11 ka BP, already showing very high CVs, with a peak ca. 10 ka BP. This is the center of the first age hiatus shown in Fig 6. The Santarém region shows a weak archaeological signal from 9 ka BP onwards, and a stronger human presence seems to appear at 8 ka BP, precisely when the CV reach its lower values. Oriximiná and Altamira (Fig 6A), on the other hand, seem antiphased in relation to Santarém. Several gaps in one area show human presence in the other, suggesting population movements along the Amazon river. For instance, the onset of maize agriculture and population growth in the Santarém area, ca. 4 ka BP, coincides with an age hiatus in both Altamira and Oriximiná.

**Fig 10:**
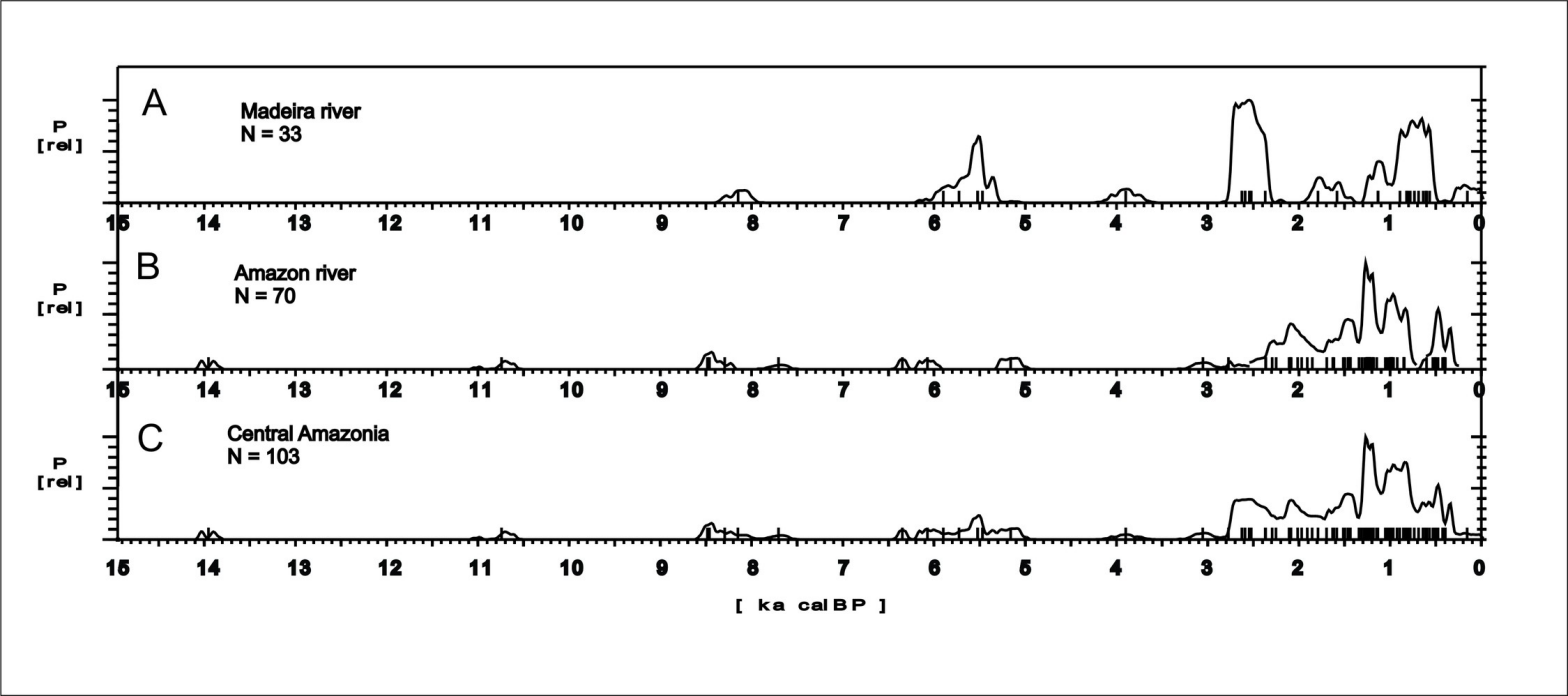
Summed probability distribution graphs for A) Madeira river area; B) Amazon river area; C) Central Amazonia (Madeira and Amazon river areas together).

**Fig 11:**
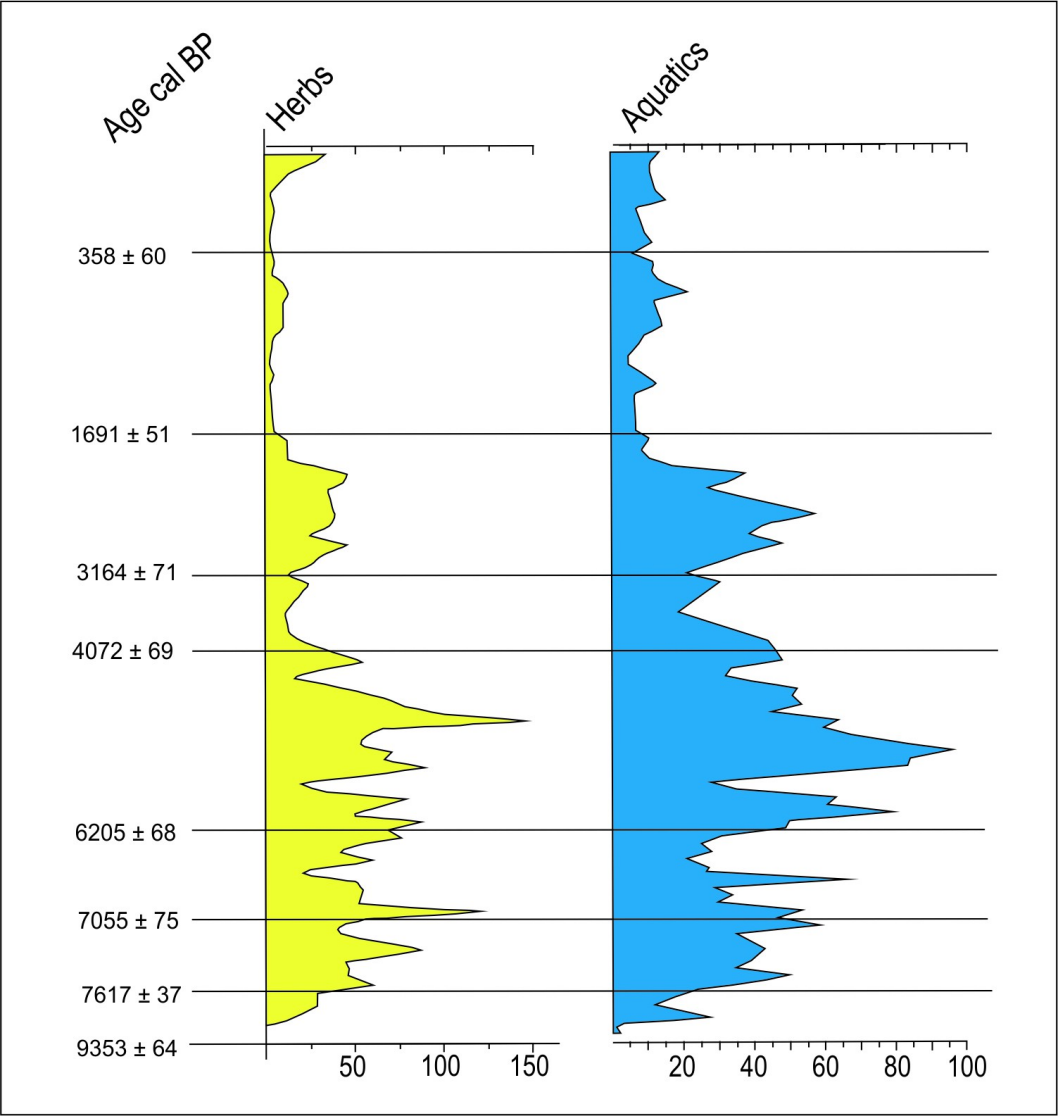
Calado lake pollen diagrams for herbs and aquatic plants, modified from Behling et al. **(2001).** It can be observed that the most variable period falls between 4 and 6 ka BP. All ages calibrated according to CalPal version 2020.11, INTCAL 2020 curve.

*Central Amazonia*. The age patterns for Central Amazonia (Figs 4 and 10C), where most sites are found along the Amazon and Madeira rivers, are somewhat antiphased in relation to Oriximiná (~450 km East) and Santarém (~ 600 km East). Central Amazonia shows some archaeological signal between 9 and 8 ka BP, when Oriximiná shows a gap, the same happening around 4 ka BP. On the other hand, a clear gap in Central Amazonia between 5 and 4 ka BP is also observed in Oriximiná. It is also possible to discern some variability along the major rivers. Sites located along the Amazon river (Fig 10B) show differences in age patterns when compared to the ones located along the Madeira river (Fig 10A). For instance, there is a cluster of ages between 6 and 5.3 ka BP in the Madeira river (coincident with one of the RCCs proposed by Mayewsky et al. [6]), when the Amazon river is depleted in the same interval. The same pattern is observed around 2.5 ka BP, when there is a peak in the Madeira river and a similar depression in the Amazon river. In fact, since 6 ka BP the Madeira and the Amazon seem antiphased. Age clusters in one area are accompanied by low age densities in the other. Again, these patterns suggest population movements whose meaning needs to be properly addressed. At ca. 0.4 ka BP the Amazon appears as depopulated (it is important to note that a null archaeological signal does not mean there were no indigenous groups in the area; It only means that their numbers were so small that they were not archaeologically visible). This feature is most probably due to European diseases and escape from slavery, while the Madeira shows a weak signal. The only paleoenvironmental study in the region was made in the Calado lake (Fig 3, number 4; [81]. The authors recognized “short periods of seasonally high-water levels and long periods of low water levels” (op. cit.: 99) since 8.5 ka BP until ca. 4.6 ka BP. From this time onwards, lake levels seemed to be higher, especially after 2 ka BP. According to Behling et al.([81]:98) the herb pollen are related to low lake stands, whereas the aquatic pollen to high lake stands. Fig 11 shows a simplified pollen diagram for Calado lake modified from Behling et al. ([81]:96), showing only herbs and aquatics. The behaviour of the pollen frequencies once again suggests strong climatic variations: the period between 4 and 6 ka BP shows the highest peaks of both herbs *and* aquatic plants.

**Fig 12:**
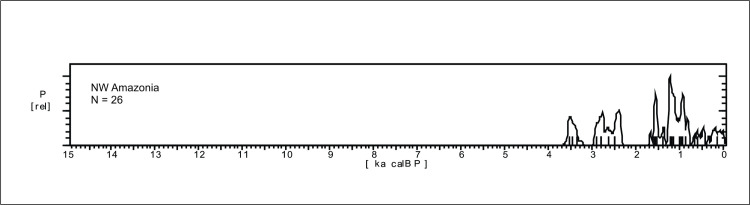
Summed probability distribution graphs for NW Amazonia.

*Northwest Amazonia*. This region is not very well known archaeologically (Figs 2 and 4), and we only have 26 ages available, ranging from 3.7 ka BP until the present. Even so, there seems to be a pattern of age gap ca. 2 ka BP (Fig 12) which is antiphased with data from the Amazon river area (Fig 10B).

A strong decrease in the archaeological signal can be perceived around 0.8 ka BP, before the onset of the European occupation in the area. Paleoenvironmental data for this vast area is related to Hill of Six Lakes area (Fig 3, number 2; Pata lake and others ‐ [66, 82, 83]). The record of Six Lakes reaches more than 50 ka BP and the discussions revolve around the LGM. We did not find detailed information about the late Holocene, which hampers comparisons with the available archaeological data.

**Fig 13:**
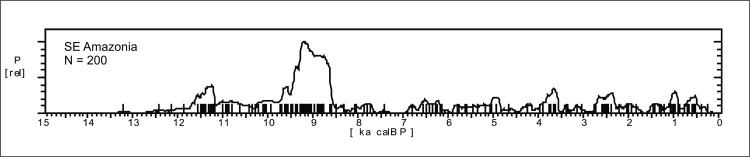
Summed probability distribution graphs for SE Amazonia (including part of Tocantins).

*Southeast Amazonia*. Southeast Amazonia (Fig 4) comprises a savannah / forest ecotone and can therefore be considered a region where climatic shifts are more visible in the pollen record [62]. We ran a Mann-Whitney U test to see if there were any significant differences between the age patterns at Carajás area and central / northern Tocantins state, and the null hypothesis was accepted (U = 3620, p = 0.615), therefore we considered the northern part of Tocantins as SE Amazonia. Fig 13 shows the age pattern for the region (n = 200), where it is possible to note a strong signal between 9.8 and 8.5 ka BP, followed by an abrupt depletion of ages that is maintained more or less at the same level throughout the middlle and late Holocene. This suggests that after a strong increase in the human population in the early Holocene, the area became less populated and remained a peripheric zone.

Several of the dated sites (n = 149) are located near Carajás, where the first paleoenvironmental studies in Amazon were carried out (Fig 3, number 7; [63]).

A gap in ages can be perceived in Fig 13 around 8.2 ka BP and between 7.3 and 6.9 ka BP, coincident with a period of forest degradation described by Turcq et al. [84]. It is important to note that at least four periods of forest degradation were detected in the Carajás lakes, three of them occurring in the late Pleistocene, but the fourth in the mid-Holocene was considered anomalous because “it does not show any marked increase in the percentage of savannah pollen (…). Moreover, the dominant arboreal pollen (…) is from *Piper* which is a low shrub pioneer in the rainforest” ([84]:140). Between “7000 and 4000 yrs BP” the authors also detected sponge spicules whose reproductive elements were incompletely developed, suggesting short inundation events and lack of nutrient supplies. Even after 4000 yrs BP, data from other cores in the area suggest that fires were frequent. The authors’main conclusion was that “the average climatic conditions were favorable to rainforest development, which was only limited by repeated occurrences of fires and dry climate events” ([84]:141). This case study is enlightening for two reasons: 1) the suppposed peak of human occupation is not related to peaks in charcoal, on the contrary; 2) the coexistence of fire events, low lake stands and the virtual absence of savannah point towards a strong climatic *variability*, and not a clear sign of dryness, the same situation we detected in the Paraíso cave record (see section 5.1.2.1.). The weak archaeological signal observed during the mid and late Holocene for SE Amazonia is, therefore, most probably related to a very unpredictable climatic scenario, with years of abundant rain followed by years of drought, affecting animal and plant resources in a human timescale, leaving a somewhat contradictory signal in the pollen record. It is also important to bear in mind that Turcq et al. [84] convey their age estimates in radiocarbon years, and it is not clear if the ages are based on organic matter or charcoal. Hence, the “7000 to 4000 yrs BP” could actually mean 7.8 to 4.5 ka BP. Taking the archaeological signal shown in Fig 13 as proxy, we would say that the onset of the mid-Holocene frequent fires and low lake stands in Carajás started at ca. 8.2 ka BP. A more recent study using geochemical analyses at Carajás [85] found evidence of a “dry” period between ca. 7 and 3 ka BP. Again, in this case the geochemical signals are somewhat contradictory since there is a major increase in organic content and, at the same time, low sedimentation rates. While the organic content suggests eutrophic conditions, the low sedimentation suggests low lake levels, and this can be understood as climatic variability, and not 4000 years of a stable, dry period.

Another palynological study was carried out at Confusão lake (Fig 3, number 35; [86]), 450 km south of Carajás. However the short sediment core obtained (the entire Holocene represented by the upper 23 cm) and some age indeterminacies hamper accurate comparisons. Even so, it is interesting to note that ca. 6.5 ka BP (5460 ^14^C years BP, interpolated) the palynological record shows, at the same time, both “high percentages of *cerrado* herbs (about 50%)” and “Amazon (…) trees and shrubs (…) (28–38%)”. Despite of the author’s interpretation of “expansion of Amazon rain forest since the mid Holocene” ([86]:35) the record can be equally understood as the result of several millennia of greatly fluctuating dry and wet periods compressed in less than 23 cm.

Lastly, at Saci lake (Fig 3, number 12; [87]) a pollen and sedimentological analysis was performed spanning the last 35 ka BP. Evidence suggests events of variable climate and droughts that correlate well with other portions of Amazonia ([87]:174). However, Saci lake is very far away from any dated archaeological site (450 km South of the Tapajós sites, and 800 km Southwest from Carajás) and we do not expect to get a reliable comparison.

**Fig 14:**
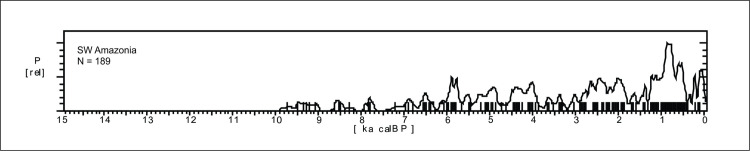
Summed probability graph for SW Amazonia.

*Southwest Amazonia*. This area comprises mostly Rondonia (RO) and Acre (AC) states (Fig 4), with 189 available ages. The Mann-Whitney U test showed no significant differences between the age patterns found in these two states (U = 1765.50, p = 0.468). The SPD diagram is presented in Fig 14 and shows a pattern without any marked gaps in the age distribution, in stark contrast to the other Amazonian regions. There are, however, probable gaps between 9 and 8.7 ka BP and again around 8.2 ka BP Some time intervals seem to signal an increase in population, such as the 7 to 4 ka BP period, which would be in contrast with Central Amazonia (Fig 10). There are two periods showing major decreases in the archaeological signal between 4 and 3 ka BP and around 1.5 ka BP (both possibly related to RCCs). A strong diminution of the archaeological signal is perceived around 0.4 ka BP, this time coinciding with the onset of the European presence, but there is an increase of ages after that, which can signal either a population recovery or the arrival of other populations displaced from elsewhere.

Paleoenvironmental studies for this region come from two sources: Vilhena-Ariquemes soil isotopes (Fig 3, number 41; [88]), and pollen from oxbow lake sediments at Humaitá, near the Upper Madeira river (Fig 3, number 5;[89]).

The 400 km SE-NW transect Vilhena-Ariquemes studied by Pessenda et al. [88] covered four types of vegetation, from wooded savanah (“cerrado”) to tropical rainforest. Soil trenches were opened and the trends of delta ^13^C were recorded. The results suggested that in the last 8 ka BP there was a trend from a C3 signature (forest) to C4 (savannah) around 6.8 ka BP, and back to C3 in the ecotone areas, and the stability of the C3 signal in the forest area. The timing of these events is not very reliable given the few radiocarbon ages available, but at least point to a probable mid-Holocene tendency towards a lengthier dry season in the ecotone between forest and savannah, and no clear changes in the forested areas. The pollen record from Humaitá suggests growing percentages in forest taxa since 10.7 ka BP ([89]:43). Both lakes at Humaitá showed gaps in the pollen assemblages (but not in the sedimentation) which suggest low lake stands (between ca. 35.2 and 7.2 ka BP in one case, and indeterminate due to age inversions in the other). However, this data should be treated with caution since low lake stands for such a long period are unlikely. In sum, the archaeological signal for SW Amazonia suggests an almost continuous human occupation, with minor paleoenvironmental impacts, which could be related to trends of slowpaced climatic changes, without the extreme variability observed in other Amazonian regions. Even so, rapid climate changes not detected by pollen or soil isotopes can be sometimes observed in the archaeological record: for instance, Miller ([90]:342) draws attention to a hiatus in the occupation of Monte Castelo shellmidden, in Rondonia, between 4350 and 4100 cal BP, where a 30 cm sterile layer separates a pre-ceramic and ceramic occupations. This is in accordance with the 4.2 ka BP rapid climate change (RCC) event [6, 91, 92].

#### Main observed paleoenvironmental and archaeological trends for the Amazonian Lowlands

The archaeological signal in different areas of the Amazonian Lowlands seem to correlate well with several published RCC events worldwide [6, 9, 93–98]. As expected, some regions show to be sensitive to RCCs that are not apparent in others. Some paleoenvironmental studies, especially the ones related to lakes, sometimes show pollinical or sedimentary gaps that span large periods of time (e.g., Comprido lake, with a gap between 7.8 and 3.0 ka), which could be either the effect of several RCCs in sequence, or just vagaries of sampling, radiocarbon dating, or age interpolation.

Table 2 summarizes the main paleoenvironmental and archaeological data for the Amazonian Lowlands and their possible relations to published RCC events.

**Table 2:** Paleoenvironmental and archaeological data for the Amazon.

Author	Area / region	Instability Age Ka	Evidence	RCC relation
Cordeiro et al 2008	Amazonian lakes	7.0 to 6.0	Low lake stands	Unclear ‐ 6.4 ka event?
Mayle & Power 2008				
Prado et al. 2013				
This paper	Santarém	11.3 to 9.2	Age SPD depletion	Yes ‐ Lake Agassiz events [9]
		8.1 to 8.4		
		6.5 to 4.5		Yes ‐ 8.2 ka event [93, 261]
				Yes ‐ 6.4 ka [97] + 5.5 ka [95]+ 5.3 ka events [97]
		2.9 to 2.5		
				Yes ‐ 3.0 to 2.3 ka event [96] + 2.8 ka event [94]
		1.5 to 1.2		
				Yes ‐ 1.4 ka event [97]
This paper	Oriximiná/Altamira	12.0 to 9.5	Age SPD depletion	Yes ‐ Lake Agassiz events [9]
		9.0 to 8.2		
		7.7 to 6.0		Yes ‐ 9 to 8 ka Glacial Aftermath [6]
		4.4 to 3.7		
				Yes ‐ 7.2 ka [41] + 6.4 ka event [97]
		2.4		
				Yes ‐ 4.2 ka event [91]
		1.5 to 1.2		Unclear ‐ 2.1 ka event?
				Yes ‐ 1.4 ka event [97]
Irion et al. 2006	Tapajós lake	ca. 8.8	Siderite peak	Unclear ‐ 9 to 8 ka Glacial aftermath?
		9.2 to 4.6	*Cecropia* pollen	
				Unclear ‐ 8.2 ka + 6.4 ka + 5.5 ka + 5.3 ka events?
Moreira et al. 2013	Comprido lake	10.3 to 7.8	Low values TOC	Unclear
		7.8 to 3.0	Sedimentation gap	Unclear ‐ 7.6 to 7.0 ka + 6.4 ka + 5.5 ka + 4.2 ka + 2.8 ka events?
Wang et al. 2017	Paraiso cave	6.0	Increase in precipitation	Unclear ‐ 5.5 ka event?
This paper	Paraiso cave	11.0 to 10.0	Strong oscillation delta ^18^O	Yes ‐ Lake Agassiz events [9]
		6.0 to 4.2		
				Yes ‐ 5.5 ka [95]+ 5.3 ka [97] + 4.2 ka event [91]
This paper	Amazon and Madeira rivers	6.0 to 5.3	Antiphasing of ages	Yes ‐ 5.5 ka event [95]+ 5.3 ka event [97]
		1.4	Age SPD depletion	Yes ‐ 1.4 ka event [97]
This paper	Central Amazonia	7.5 to 6.5	Age SPD depletion	Yes ‐ 7.6 to 7.0 ka [41] + 6.4 ka event [97]
		5.0 to 4.1		
				Yes ‐ 4.2 ka event [91]
		3.7 to 3.2		Yes ‐ 3.8 to 3.1 ka event [96]
		1.4		
				Yes ‐ 1.4 ka event [97]
Behling et al. 2001	Calado lake	6.0 to 4.0	Pollen signal oscillations	Yes ‐ 5.5 ka [95]+ 5.3 ka [97] + 4.2 ka events [91]
Turcq et al. 1988	Carajás lake	7.8 to 4.5	Pollen and sponge spicules	Yes ‐ 7.6 to 7.0 ka event [41] and 5.5 ka event [95]
This paper	SE Amazonia	8.2	Age SPD depletion	Yes ‐ 8.2 ka event [93]; [261]
		7.3 to 6.9		Yes ‐ 7.6 to 7.0 ka event [41]
Guimarães et al. 2016	Carajás lake	7.0 to 3.0	Contradictory geochemical signals	Unclear ‐ 5.5 ka and 4.2 ka events?
Behling 2002a	Confusão lake	ca. 6.5	Contradictory pollen signals	Yes ‐ 6.4 ka event [97]
Fontes et al. 2017	Saci lake	7.5 to 5.0	Pollen and charcoal	Yes ‐ 7.6 to 7.0 ka event [41] and 5.5 ka event
This paper	SW Amazonia	9.0 to 8.7	Age SPD depletion	Yes ‐ 9 to 8 ka Glacial Aftermath [6]
		8.2		
		4.0 to 3.0		Yes ‐ 8.2 ka event [93, 261]
		1.5		Yes ‐ 3.8 to 3.1 ka event [96]
				Yes ‐ LALIA [179] and 1.4 ka event [97]
Miller 2013	Monte Castelo site	4.3 to 4.1	Occupation hiatus	Yes ‐ 4.2 ka event [91]

### Brazilian nordeste

Currently the Nordeste (NE Brazil) is the region that receives the lower amount of rainfall in the Brazilian territory, with a mean annual precipitation of 1800 mm in the coast but reaching only 400 mm in the hinterlands, presenting a xeric vegetation that covers 60% of the area [99], ranging from scrubland to dry savannahs (*Caatinga* biome), with very localized patches of woody vegetation.

#### Paleoenvironments

The Nordeste is a region considered to be “antiphased” in relation to other regions [28] Smith and Mayle 2018). There is a relatively good agreement between different proxies [27, 100–103], signalling a wet mid-Holocene in contrast with increasing aridity in the late Holocene, which confirms an opposing trend in relation to Amazonia. However, it is important to note that local / regional variations are very marked in this region. Auler and Smart [104] also point to the importance of regional differences in climate that can be superimposed over general atmospheric circulation patterns. Nimer [105] observes that today the rain patterns in NE Brazil are significantly conditioned by orography. The distribution of the dry season is very variable, with mountains being responsible for the abbreviation of the dry season, and plains responsible for its extension. There are also marked contrasts between the coast and the hinterland. We can reasonably expect that these patterns can be extended to the past, and in fact this was observed by Montade et al. (2014) regarding a 5 ka BP lake record (Maranguape lake). Another example comes from Catimbau swamp, which nowadays is a 3 km strip of arboreal vegetation in the middle of a semi-arid region [106]. Even leeward and windward sides of a same mountain will show marked precipitation, soil, and vegetational contrasts [107, 108].

#### Archaeological data

**Fig 15:**
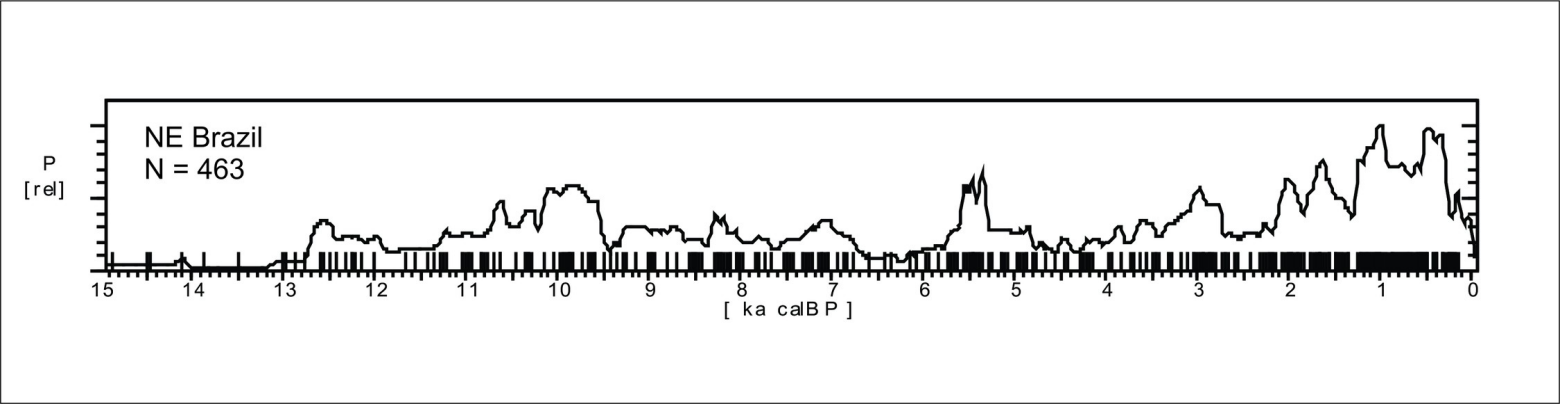
Summed probability graph of 463 ages for NE Brazil.

The summed probability graph comprising 488 ages for the Nordeste is shown in Fig 15. The graph comprises only the last 15 ka BP, and older ages will not be discussed in this paper. It is possible to note that the age pattern is somewhat different from Amazonia (Fig 4), although there are similarities such as the onset of marked human presence at ca. 12.8 ka BP. While Amazonia showed a two-peaked pattern in the early Holocene, the Nordeste showed a steady increase in the human signal until ca. 9.5 ka BP, followed by an oscillating curve without apparent marked decreases, and an age cluster around 6 and 4.8 ka BP. Our results differ from a recent paper by Utida et al. [109], where the authors made a compilation of archaeological ages for NE Brazil and perceived “a high frequency of radiocarbon dates during the Early and Late Holocene and a low frequency during the Middle Holocene” ([109]:14). Since their database comprised only 267 ages, we believe their conclusions suffer from a sample size effect. Moreover, as we will show, to rely on a general pattern for NE Brazil, be it archaeological or paleoenvironmental, is misleading since it masks important differences among regions.

#### Regional age patterns in Nordeste

In order to explore potential differences in the age patterns we first sub-divided the Nordeste into three regions: 1) Western, comprising Maranhão and Piauí states; 2) Eastern, comprising Rio Grande do Norte, Paraiba, Ceará, Pernambuco, Sergipe, and Alagoas states; 3) Southern, comprising Bahia State (Fig 4).

**Fig 16:**
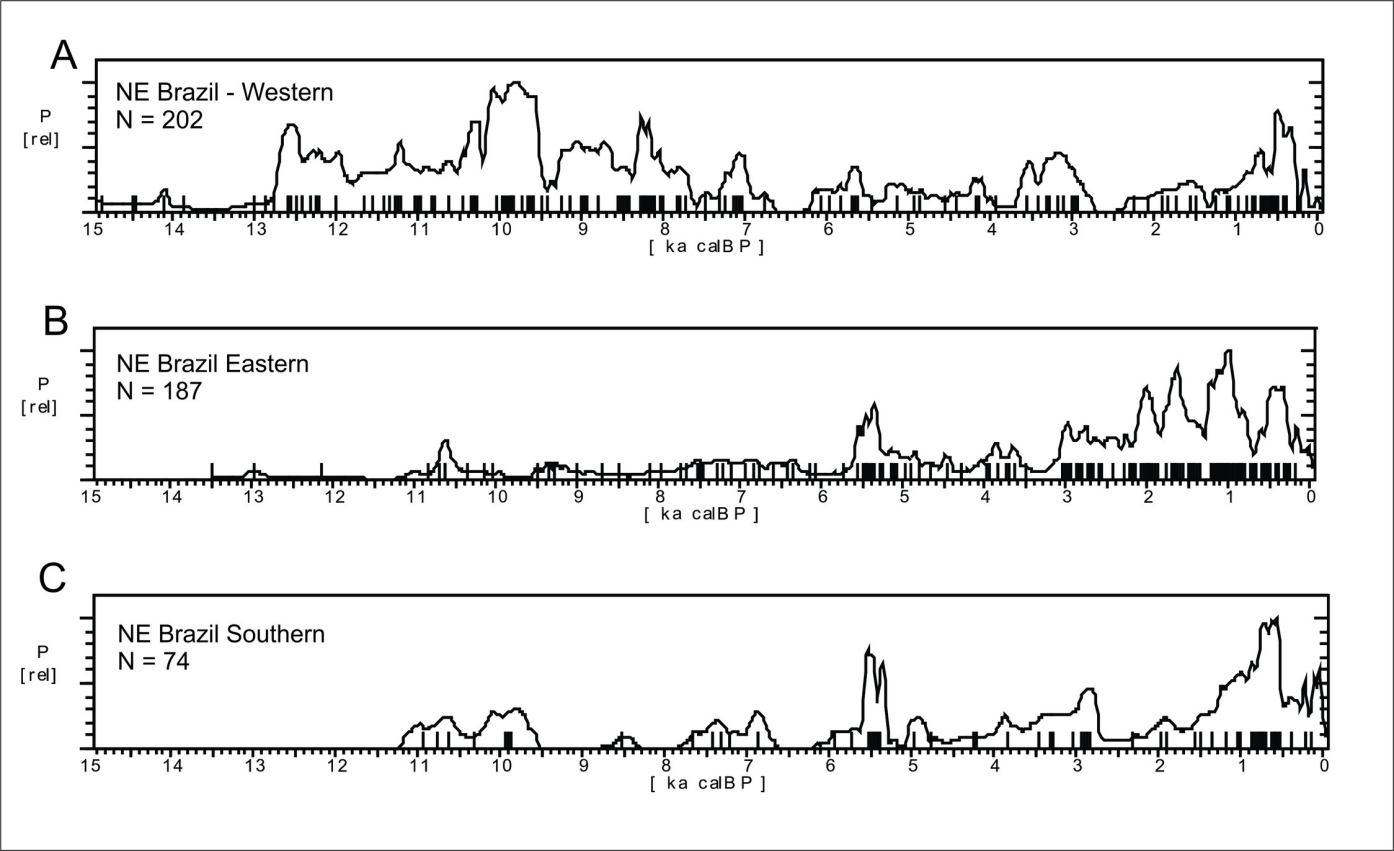
Summed probability graph for the eastern and western portions of NE Brazil. A) Western: Maranhão and Piaui states, 202 ages. B) Eastern: Rio Grande do Norte, Paraiba, Pernambuco, Ceará, and Alagoas states, 187 ages; C) Southern: Bahia State, 74 ages.

Fig 16 shows the SPD graphs for each region. The Mann-Whitney U test showed significant differences between the age patterns found in the Eastern and Western regions (U = 8392, p < 0.0001), granting that they must be treated separately. However, the Eastern and Southern patterns are very similar and can be treated as a single group (U = 6388, p = 0.4385).

The Western Nordeste shows a strong human presence between 12.8 and 7.7 ka BP. It is important to note that this region shows a strong archaeological signal during the Younger Dryas (YD) cold event, a period of marked and rapid climatic change between 12.9 ka BP and 11.6 ka BP (Carlson, 2010), something that is not very common in other regions of Eastern South America. On the other hand, the curve shows a marked hiatus between 7 and 6.2 ka BP, whereas the Eastern portion showed a much weaker and oscillating signal during the early to mid-Holocene, without any marked hiatus, and an age cluster between 5.6 and 5 ka BP. There is a marked growth in the Eastern curve ca. 3 ka BP which seems antiphased with the Western curve. In the last 3 ka BP the Eastern portion seems more occupied by humans than the Western one.

The paleoenvironmental background is somewhat scarce in the Western region; Caçó lake (Fig 3, number 19) and vicinities was studied by several authors [70, 111, 112]. The authors interpreted the paleoenvironment as passing through a major climatic disruption between 12.8 and 11 ka BP, when forest is rapidly replaced by an open vegetation “within a 100 yr time period” ([110]:1118). Between 11 and 8.5 ka BP the forest taxa increase again, together with other indicators of increasing moisture. From 8.5 ka BP to the present there is an increase in savannah taxa, and other proxies such as the *Botryococcus* algae attest frequent episodes of low lake stands (see Fig 6 from [110]:1117). In spite of the very detailed data and good chronological coverage, we have three problems in order to relate Caçó lake data and archaeological age patterns: the proximity of the lake to the coast (~70 km), which imparts a milder climate, subject to coastal umidity, in contrast with the archaeological sites that are located much further inland; the distance of more than 600 km between the lake and the nearest sites; and the mismatch between the chronology obtained at the lake (radiocarbon on soil organic matter) versus the radiocarbon obtained on charcoal at the archaeological sites.

Regarding the Eastern Nordeste, we have the Chapada do Apodi caves, located 700 km towards East ([109]; Fig 3, number 25). Data from Chapada do Apodi can be contrasted with Caçó Lake. While there is some agreement between proxies in the Late Glacial, for instance a hiatus in the speleothem growth at Apodi between 15 and 13 ka BP ([109]:10) matching two episodes of “abrupt forest regression” in Caçó at 15 and 13.5 ka BP ([110]:1115), the same cannot be said for the Holocene. For instance, while Utida et al. ([109]:12) mention that cave drainage was very active between 8 and 4.2 ka BP, and possibly much drier conditions prevailed since 4.2 ka BP, data from Caçó suggest frequent episodes of low lake stands from 8.5 ka BP until recent times, without a signal of marked dryness. These differences can account for the different archaeological age patterns between the Eastern and Western regions on one hand, but on the other hand we do not see a good match between the Eastern age patterns and the Chapada do Apodi caves. For instance, the marked 4.2 ka BP event with the onset of drier conditions present in the Apodi records is not perceived in the archaeological age curve. Here lies a potentially interesting example of the impact of RCCs versus “dryness” hypothesis. The 4.2 ka RCC is well marked in the archaeological signal (no ages at this period, as can be seen in Fig 16B), followed by an increase of ages between 4 and 3.5 ka BP, when the climate could dry, but perhaps *consistently* dry, allowing human groups to cope with predictable resources. Another possibility is that the archaeological data is more in accordance with the results obtained by Nascimento et al. [106] at Catimbau, Pernambuco (Fig 3, number 30). The authors interpret the lake record as showing an increase in moisture from 6.8 ka BP to 1.6 ka BP (interpolated ages).

In Southern Nordeste there is a partial match between the available paleoenvironmental studies [113–116] and the archaeological patterns. For instance, at Icatu (Fig 3, number 32) De Oliveira et al. [115] presented a combined record of dune activity and pollen suggesting an early to mid-Holocene humid climate, with no dune formation around 10 ka BP, coincident with a cluster of archaeological ages (Fig 16C). There is a change towards semi-arid conditions around 6.5 ka BP, marked by absence of pollen, and accordingly in Fig 16C it is possible to see a hiatus in the ages. However, it should be noted that there are no dated archaeological sites close to Icatu. In fact, the age patterns observed in Fig 16C after the mid-Holocene are antiphased with the Icatu record. The largest peak of dune activity at Icatu was recorded between 4 and 2 ka BP, and this is when we have an increase in the ages in the sites located towards East.

We believe the reason for the paleoenvironmental inconsistencies in Nordeste lies in the abovementioned high orographic-related climatic differences. It is possible that human populations were shifting their settlements between areas located a few kilometers away, which would cause a blurring of the patterns and, at the same time, a lack of match with paleoenvironmental studies. As an upshot, it is possible that paleoenvironmental studies in Nordeste cannot be extrapolated too far away from the collection points. In their compilation of 120 paleoclimatic datasets for the mid-Holocene in Eastern South America, Prado et al. [117] also perceived that, contrary to other regions that showed clear signals of increase in dryness around 6 ka BP, “northeastern Brazil (…) exhibits an unclear climate signal” ([117]:2121).

#### Main observed paleoenvironmental and archaeological trends for the Brazilian Nordeste

In spite of the absence of an overall climatic pattern for the Nordeste, each region showed a very good agreement between archaeological and paleoenvironmental data. For Western NE, all major depressions in the age SPD curves could be correlated to at least one RCC, with the possible exception of the 3.9 to 3.7 ka interval. For Eastern NE the same can be said, with the only lack of correlation occurring in the interval between 3.5 to 3.0 ka, which could be tentatively correlated with the 3.6 ka event [12]. For Southern NE, the correlation with RCCs could be assigned for all the five SPD depressions (see Table 3).

**Table 3:** Paleoenvironmental and archaeological data for the Nordeste.

Author	Area / region	Instability Age Ka	Evidence	RCC relation
This paper	Western NE	14.8 to 13.3	Age SPD depletion	Yes ‐ 14.8 ka climate shifts, Meltwater pulse 1A [39, 40, 260]
		9.8		
		7.6		Yes ‐ Meltwater pulse 1C [8]
		6.6 to 6.3		Yes ‐ Melwater pulse CRE3 [8]
		3.9 to 3.7		Yes ‐ 6.4 ka event [97]
		2.7 to 2.4		Unclear
		1.3		Yes ‐ 3.0 to 2.3 ka event [96]
				Yes ‐ 1.4 ka event [97]
Jacob et al. 2004	Caçó lake	12.8 to 11.0	Rapid pollen taxa change	Yes ‐ 11.8 ka climate excursion [250]
Ledru et al. 2006				
Pessenda et al. 2005				
This paper	Eastern NE	11.6 to 11.2	Age SPD	Yes ‐ Meltwater pulse 1B [8]
		9.9 to 9.6	depletion	Yes ‐ Meltwater pulse 1C [8]
		6.0 to 5.5		Yes ‐ 5.5 ka event [95]
		4.3 to 4.0		Yes ‐ 4.2 ka event [91]
		3.5 to 3.0		Unclear ‐ 3.6 ka event?
		1.4		Yes ‐ 1.4 ka event [97]
This paper	Southern NE	9.5 to 8.8	Age SPD depletion	Yes ‐ Meltwater pulse MWP3 [8]
		8.3 to 7.8		
		6.6 to 6.2		Yes ‐ 8.2 ka event [93]
		5.2		Yes ‐ 6.4 ka event [97]
		2.8 to 2.2		Yes ‐ 5.3 ka event [97]
				Yes ‐ 3.0 to 2.3 ka event [96]

### Central Brazil

This area is mostly covered with *Cerrado*, which presents the highest floristic richness among the world’s savannahs [118], with a mean annual precipitation of 1500 mm and climate classified as Aw (tropical with a dry season in the winter) according to the Koppen classification [119, 120]. Rainfall is heavily influenced by the South American Summer Monsoon (SASM) activity, and this phenomenon can be tracked back to the Pleistocene / Holocene transition [121].

#### Paleoenvironments

There were some controversies regarding the signal of the climatic variations across the late Pleistocene / Holocene for this vast region (see S4 File).

Some possible explanations for these interpretive discrepancies were presented in Araujo et al. ([22]:299), and were interpreted as problems in comparability of pollen data due to different research methods, problems of chronology related to the interpolation of C14 dates or to radiocarbon reservoir effect, or even to variations in localized, differential moisture gradients due to microclimatic/orographic factors. Although we maintain that these problems can still play their role, more recent work tends to suggest, again, that climatic variabiliririty rather than sustained droughts can be the key in interpreting the often conflicting scenarios that different researchers tend to present [24, 122, 123]. Papers published in recent years are more aware of these factors [121, 124].

#### Archaeological data

**Fig 17:**
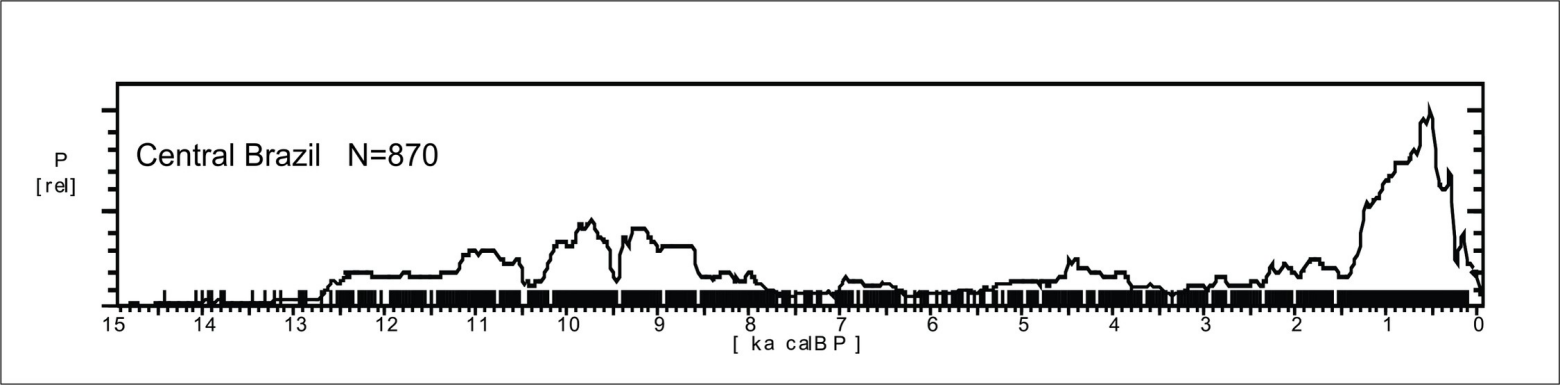
Summed probability graph of 870 ages for Central Brazil. Ages older than 15 ka BP are not shown.

The SPD graph of the 870 ages available for Central Brazil are shown in Fig 17. The pattern is very different from Amazonia (see Fig 4 for a comparison), with a very stable pattern in the early Holocene, suggesting a well-developed and increasing population since at least 12.7 ka BP, followed by a depression ca. 8.6 ka BP and a somewhat lower signal throughout the mid-Holocene, with a small increase in ages between 4.5 and 3.8 ka BP. The archaeological signal starts to increase steadily about 2.3 ka BP and shows a major peak between 1.4 and 1.2 ka BP. As we showed previously, the signal is much stronger in Amazonia since 2.8 ka BP, therefore it is possible that the lagged signal increase in Central Brazil is related to the expansion of horticulturalists outside Amazonia [125]. The steep growth is interrupted at 500 cal BP, consonant with the arrival of Europeans.

**Fig 18:**
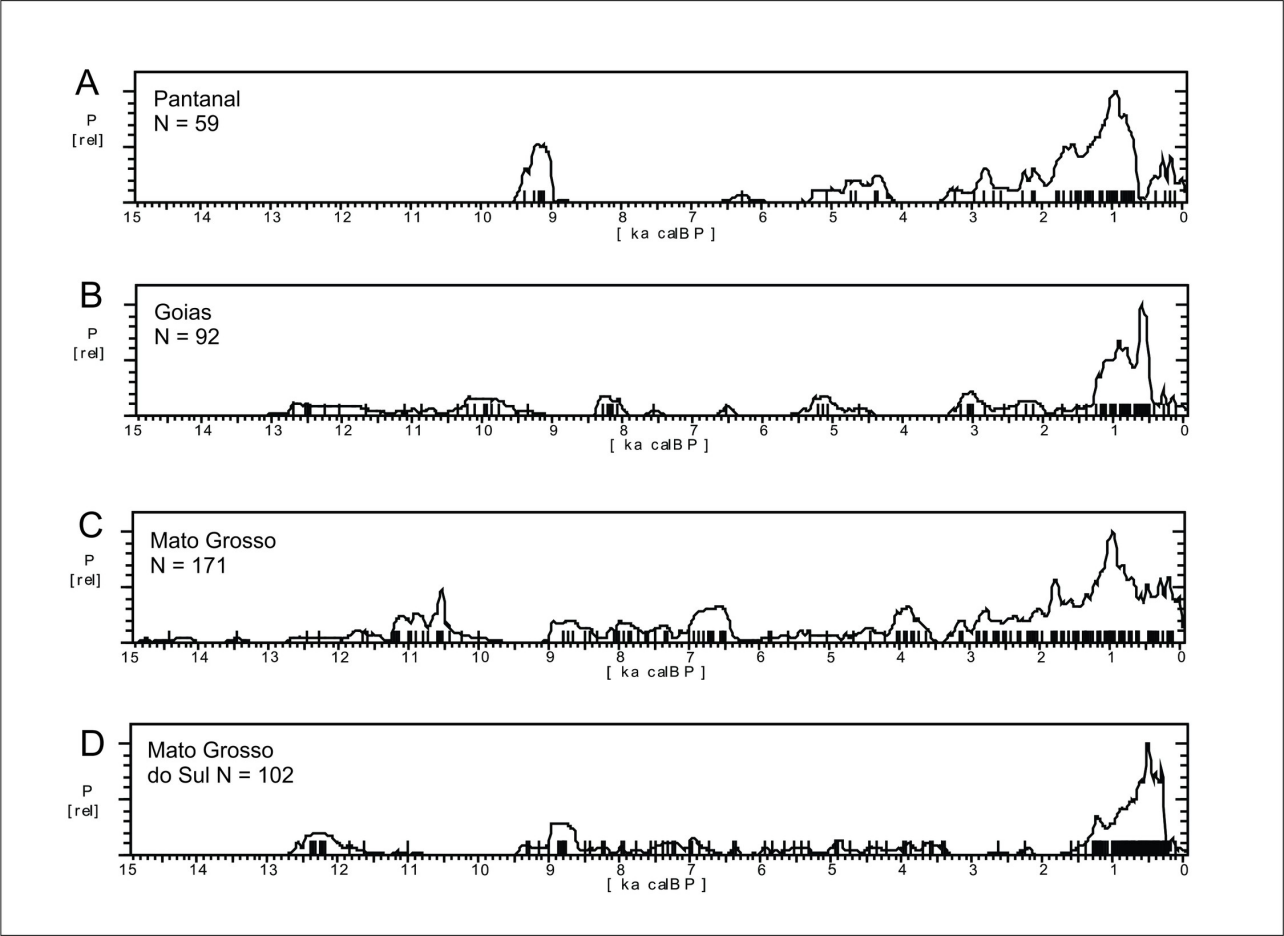
Summed probability distribution graphs for A) Pantanal; B) Goiás (GO); C) Mato Grosso (MT); D) Mato Grosso do Sul (MS). Ages older than 15 ka BP are not shown.

*Western Central Brazil*. This area comprises Mato Grosso (MT), Mato Grosso do Sul (MS), Goías (GO), and the Pantanal, which comprises the western portion of MT and MS (Fig 4). The Pantanal shows a marked dry/wet cycle, very dependent on the rainfall regime [126], and therefore presenting a very distinctive vegetation (Seasonally Dry Tropical Forest–SDTF; [127]), different both from the Cerrado and from the Amazonian Rainforest. Fig 18 shows the age patterns for each area: MT and Pantanal on the West, GO towards East, and MS towards South. It is possible to perceive that there is a strong antiphased pattern between Pantanal and the other areas, where a cluster of ages between 9 and 9.5 ka is present at Pantanal but absent at GO and MT, and weak at MS. The reverse occurs between 6.5 and 9 ka BP, where Pantanal seems to be depopulated, GO shows a somewhat weak signal, while MT and MS show a stronger signal. It is also important to compare GO and MT, who also show antiphased curves. For instance, there is a small shift of ages around 8.2 ka BP, when a cluster of ages in GO coincides with a hiatus in MT, and this East-West antiphased pattern is observable between these two regions throughout the Holocene (8.5 to 9 ka BP; 6.5 to 8 ka BP; 3.5 to 4.5 ka BP; 1.3 to 2 ka BP). Between 3.5 and 2 ka BP, MS shows a very weak signal, antiphased with the other three regions.

Starting with the Pantanal, it must be taken into consideration that even today the region can be subject to extremes of temperature, from 44° C in the summer to 10°C or less in the winter, depending on the strength of the polar fronts [128]. From the human point of view such extremes, if enhanced by even more extreme fluctuating conditions throughout the Holocene, could be very problematic.

**Fig 19:**
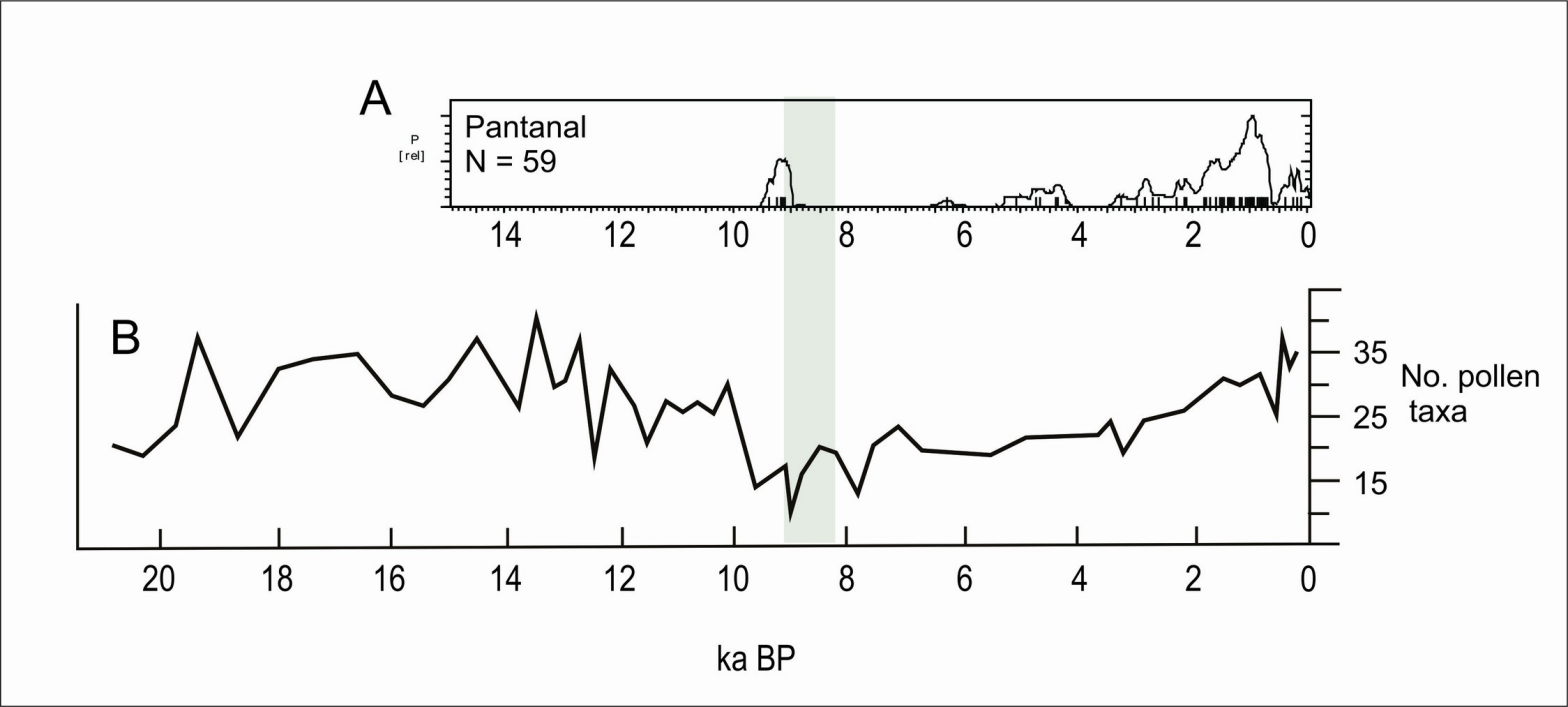
A) Age distribution for the Pantanal compared with B) the number of pollen taxa for La Gaiba lake (modified from Whitney et al 2014). The lowest number of taxa can be considered as a proxy for the occurrence of extreme climatic events. At 9 ka BP the number of taxa was even lower than during the LGM.

The most complete multiproxy paleoenvironmental record for the Pantanal comes from La Gaiba lake [127, 129] (Fig 3, number 79). Data from La Gaiba suggest a dryer LGM ([129]; also corroborated further south at Nhecolândia [130] Fig 3, number 76; and at Nabileque [131] ‐ Fig 3, number 73) followed by an increase in temperature (but not in precipitation [129]) in the Late Glacial, from 19.5 until 12.2 ka BP. Around 12.2 ka BP there was an increase in precipitation, with Pantanal floods reaching higher levels. Beginning at 10 ka BP there are signals of an increasingly “dryer or more seasonal climate (…) [with] a floristic shift towards a more drought-tolerant/adapted SDTF community”with a peak ca 6.4 ka BP ([129]:189). Whitney et al. [132] corroborated the earlier results and added that although “mean lake levels, a proxy for total annual precipitation at this site, remained relatively high throughout the Holocene (…), we infer that these changes in the SDTF flora may have been driven by increased drought manifested as a longer dry season” (op. cit.: 10). Of course, this could be also related to higher seasonal variability, and not necessarily to an increase in the lenght of the dry season alone. Supporting our interpretation, the same authors state that “floristic changes suggest that mid-Holocene drying, manifested through an extended dry season, caused greater drought stress on the Chiquitano SDTF compared with the late Pleistocene when total rainfall was demonstrably lower” (op.cit.: 10). Lastly, Whitney et al. ([132]:10) also observe a decrease in the palynological richness in the early to mid-Holocene, and relate it to “enhanced drought and/or a lengthened dry season” which negatively influenced plant diversity. Again, extreme and unpredictable climatic events could be the case here, providing a better explanation for the loss in plant diversity due to ecological stress. Fig 19 shows the comparison between the number of pollen taxa and the age distribution. It can be clearly perceived that the abrupt disappearance of archaeological sites at 9 ka BP coincides with the period where the number of pollen taxa reaches the lowest value, even lower than what was detected during the LGM. After 3.5 ka BP the palynological richness steadily increases ([132]:10), suggesting a climatic amelioration that is also mirrored in the age distribution curve (Fig 19).

Another multiproxy study was carried out by McGlue et al. [133] at La Gaiba and Mandioré lake (Fig 3, number 80). The authors found several episodes of sedimentar hiatuses pointing towards extreme droughts, one of them younger than 4.7 ka BP, and the other younger than 5.3 ka BP. The authors also detected a major shift in the sedimentary record ca. 9 ka BP, with a peak in oxidized sand grains and charcoal input that suggest very low lake stands and fire ([133]:289), which coincides both with the low number of pollen taxa detected by Whitney et al. [132] and the absence of archaeological signal (Fig 19).

There is evidence of both drought and higher precipitation events in the Pantanal during the mid-Holocene. For instance, there is a decrease of the riverine system and the formation of lakes inside abandoned river channels, around 6.5 ka BP [131], or even later (3.9 ka BP [134]). Rasbold et al. [135] describe a sedimentation hiatus at Negra lake (Fig 3, number 71) between 5.7 and 3 ka BP, and also the strong presence of sponge gemmuloscleres (indicative of lake drying) between ca. 7.5 and 5.7 ka BP. Based on carbon content from sediments of Cáceres lake (Fig 3, number 81), Rasbold et al. [136] suggest a period of very shallow lake levels between 7.3 and 6 ka BP. At the same time, there is evidence of very high river levels, with the deposition of carbonate tufas at Serra da Bodoquena [137] (Fig 3, number 77), with ages between 6.5 and 0.6 ka BP. These somewhat conflicting proxies suggest rapid, fluctuating climatic conditions that can be responsible for the weak (and even absent) archaeological signal between 9 and 2.5 ka BP. According to Metcalfe et al. [138] the extant conditions reigning in the Pantanal can be tracked back to 2.1 ka BP (or 2.6 ka BP [133]). This could explain the rising of the archaeological signal in the last two millennia.

Paleoenvironmental studies for Goiás are fewer: Águas Emendadas swamp, Cromínia swamp, and Feia lake (Fig 3, numbers 107, 45, and 46).

At Águas Emendadas, Barberi et al. [139] found a 14,200 years sedimentation hiatus between 21.5 and 7.2 ka BP. Their interpretation was that “a semi-arid climate” reigned during this period. However, these data must be taken with caution in terms of their representativeness. As the authors stated, the plateau (1030 m a.s.l.) where Águas Emendadas is located shows, nowadays, “the most severe conditions for cerrado vegetation; they do not occur in other sites previously studied” ([139]:251). This suggests that conditions signalling severe dryness affecting the plateau probably cannot be extrapolated over wide areas, as it became clear when other regions nearby were studied [121, 140]. In fact, the archaeological data shows a steady (albeit not very strong) signal from 12.7 to 9 ka BP (Fig 18B), well inside the “semi-arid” conditions of Águas Emendadas.

Cromínia swamp has a continuous record between 36 ka BP and 7.5 ka BP, and in contrast with the abovementioned Águas Emendadas record, shows evidence of drying in a much shorter interval, between ca. 10.5 ka BP (extrapolated) and 7.5 ka BP [141]. Unfortunately, this study suffers from few radiocarbon ages (only one age for the late Pleistocene, and one for the Holocene), and therefore the age intervals are widely extrapolated.

**Fig 20:**
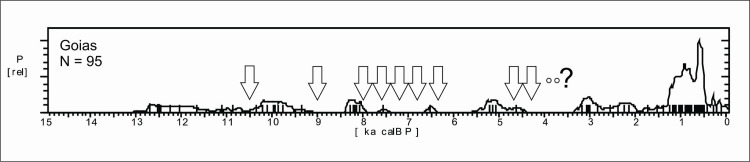
Age distribution for Goiás and peaks of Ti and Fe concentrations (arrows) found at Feia lake (after Cassino et al. 2020). The Ti and Fe peaks are probably related to events of rhesistasy, when soil erosion / deposition is stronger. Ages calculated for the events are 10.5 ka BP; 9.1 to 8.8 ka BP; 8 to 6.8 ka BP; 6.4 ka BP; and before 4.7 ka BP.

Feia lake is located only 30 km East of Águas Emendadas, but in a lower topographic setting (870 m a.s.l.). It was first published by Ferraz Vicentini [142] and later by Turcq et al. [140] who studied a core spanning the last 11 ka BP without discontinuities. Ferraz Vicentini [142] studied the uppermost 375 cm of the core, spanning the last 6 ka BP, and found episodes of high charcoal input between 5.8 and 5.7 ka BP, 4.3 and 4.1 ka BP, and 3.4 to 3.1 ka BP. All these intervals are coincident with the absence of archaeological signal (Fig 18B), suggesting that the fires were driven by climatic stress, and not human action. Turcq et al. [140] found high levels of total organic carbon (TOC) across the profile, except for one episode, ca. 9.7 ka BP, which was interpreted as a strong input of clastic mineral grains (quartz, gibsite, and goethite), which suggests vegetation distress and erosion due to a rapid climate change episode. This event coincides, again, with a decrease in the archaeological signal, which started to increase at 10.5 ka BP and is absent after 9 ka BP. More recently, another core was extracted from the lake with ages between 18.5 and 5.0 ka BP, and multiproxy studies were carried out [121, 143]. The results suggest strong shifts between wet and dry episodes within a somewhat dryer period between 11 and 7 ka BP. Several samples along the core showed sterile layers, suggesting lake desiccation ([121]:7). Peaks in Ti and Fe, probably signaling soil remobilization and depositional events related to rhesistatic conditions, were recorded at some intervals (10.5 ka BP (9280 ± 50 ^14^C BP); 9.1 to 8.8 ka BP (8195 ± 50 to 8030 ± 35 ^14^C BP); 8 to 6.8 ka BP (7240 ± 45 to 5985 ± 30 ^14^C BP); 6.4 ka BP (5630 ± 35 ^14^C BP); and before 4.7 ka BP (4215 ± 30 ^14^C BP); ([121]:4–5). Again, these peaks match well the several instances of diminution or absence of the archaeological signal (Fig 20).

Paleoenvironmental data for Mato Grosso is scant, and comprises a single stalagmite from Pau D’Alho cave (Fig 3, number 36; [144], which covers only the last 1.4 ka BP. Mato Grosso do Sul has data coming from Jaraguá cave on the West (Fig 3, number 78; [145, 146]) and at Taquarassu, near the Paraná river on the East (Fig 3, number 75; [147, 148].

The Pau D’Alho cave record shows fluctuations that suggest sensitivity to both the Medieval Climate Anomaly–MCA (ca. 900 to 1100 AD) and to the Little Ice Age ‐ LIA (ca. 1600 to 1820 AD). Based on ^18^O isotope data, Jaqueto et al. ([144]:7028) posited that the MCA was a dryer period. Again, an analysis of the raw data [144] shows an increase of the ^18^O variability (CV) between 1.2 and 1.0 ka BP. This coincides with the MCA, with a decrease in ages for Mato Grosso and an increase in ages for Mato Grosso do Sul (Fig 18B and 18C), suggesting population movement southwards.

The Jaraguá cave record comprises both stalagmite isotopes and sedimentary analysis [149]. The isotope data is based on ^18^O and ^13^C, covering the last 28 ka [145, 146], but we will center our analysis on the last 15 ka BP, using the raw data provided by Novello et al.[149]. The authors concluded that the Holocene was dryer than the late Pleistocene and remarked that their data did not fit the paleoenvironmental scenario proposed by other authors for the Pantanal, who proposed the contrary, based on biological proxies [127, 129, 132]. As stated by Novello et al. ([145]:3) “vegetation in the region may not have responded primarily to changes in precipitation”. One of the most relevant conclusions by Whitney et al. [132] is that the floristic composition at La Gaiba lake was much more affected by temperature than by precipitation. The temperature rising after 19.5 ka BP (estimated in 4° C) imparted a much more pronounced vegetation shift than the rising in precipitation at 12.5 ka BP; the forest started to grow at 19.5 and not at 12.5 ka BP, probably due to the end of frost events.

**Fig 21:**
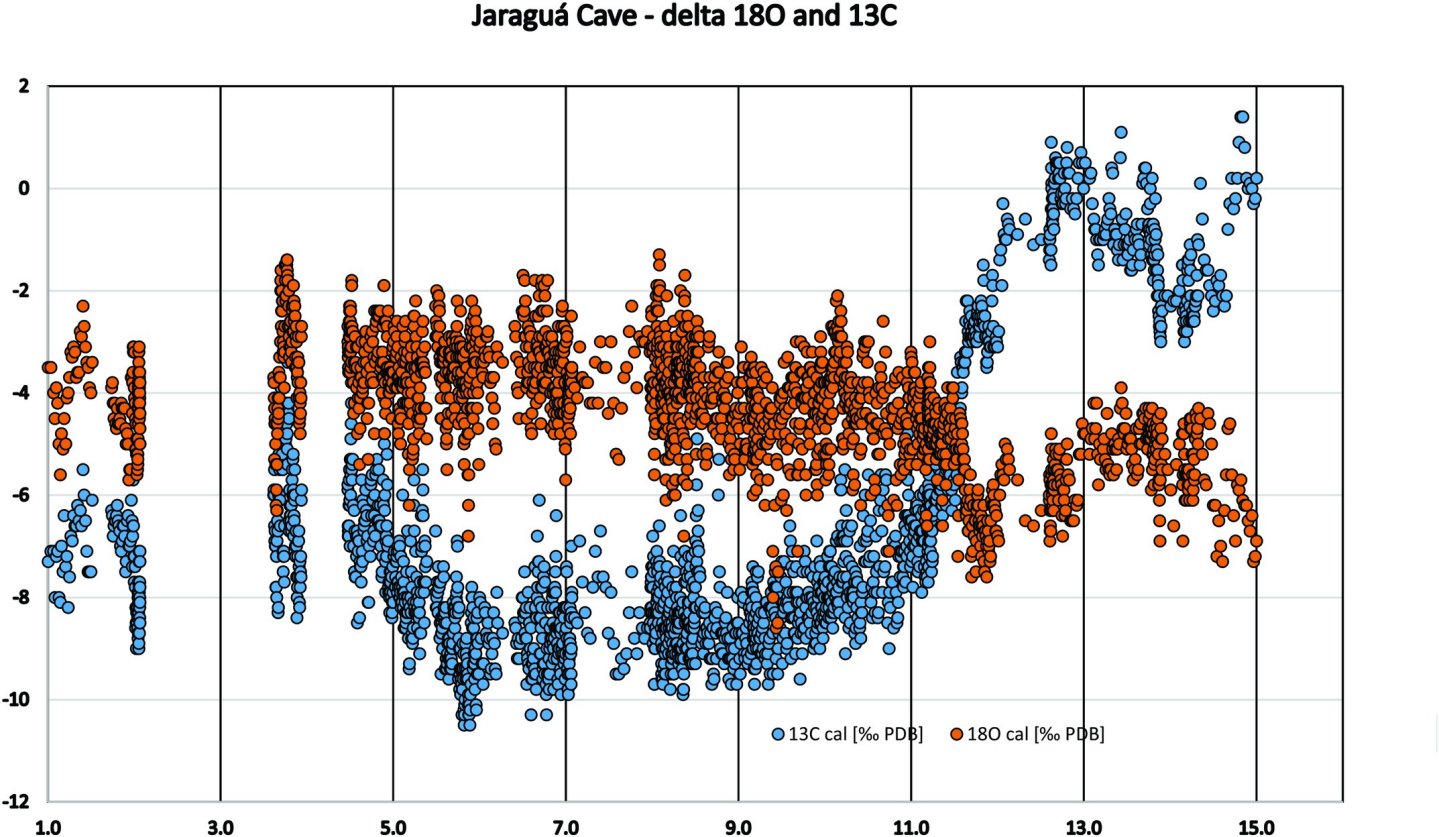
Jaraguá cave. Plot of delta ^18^O (orange dots) and delta ^13^C (blue dots) values across the last 15 ka BP, after Novello et al. (2019). There is a strong correlation among the proxies since 4 ka BP, but a lack of correlation before. Higher values of delta ^18^O would mean lower precipitation, while higher values of delta ^13^C would mean less trees. Note that betwwen ca. 12 ka BP and 4 ka BP the proxies suggest less precipitation but more trees.

If we take it that ^18^O is considered as a proxy for precipitation and ^13^C as a proxy for vegetation [149–151], the most surprising feature is the general lack of correlation between them (Spearman r = -0.109; N = 2383). Fig 21 shows the plot of ^18^O and ^13^C across the last 15 ka BP. It is possible to perceive that the correlation is variable: strong and positive in the last 4 ka BP (Spearman r = 0.471; N = 553), but very weak between 12 and 4 ka BP (Spearman r = 0.018; N = 1559), and between 15 and 12 ka BP (r = 0.045; N = 271). However, the most striking feature shown in Fig 21 is the fact that, according to the proxies, between ca. 12 ka BP and 4 ka BP less precipitation would be accompanied by more C3 plants (trees). This suggests a more complex interplay between factors, and that ^18^O values cannot be related to paleoenvironments in a direct manner, something that is starting to be acknowledged in the recent literature [152–154].

**Fig 22:**
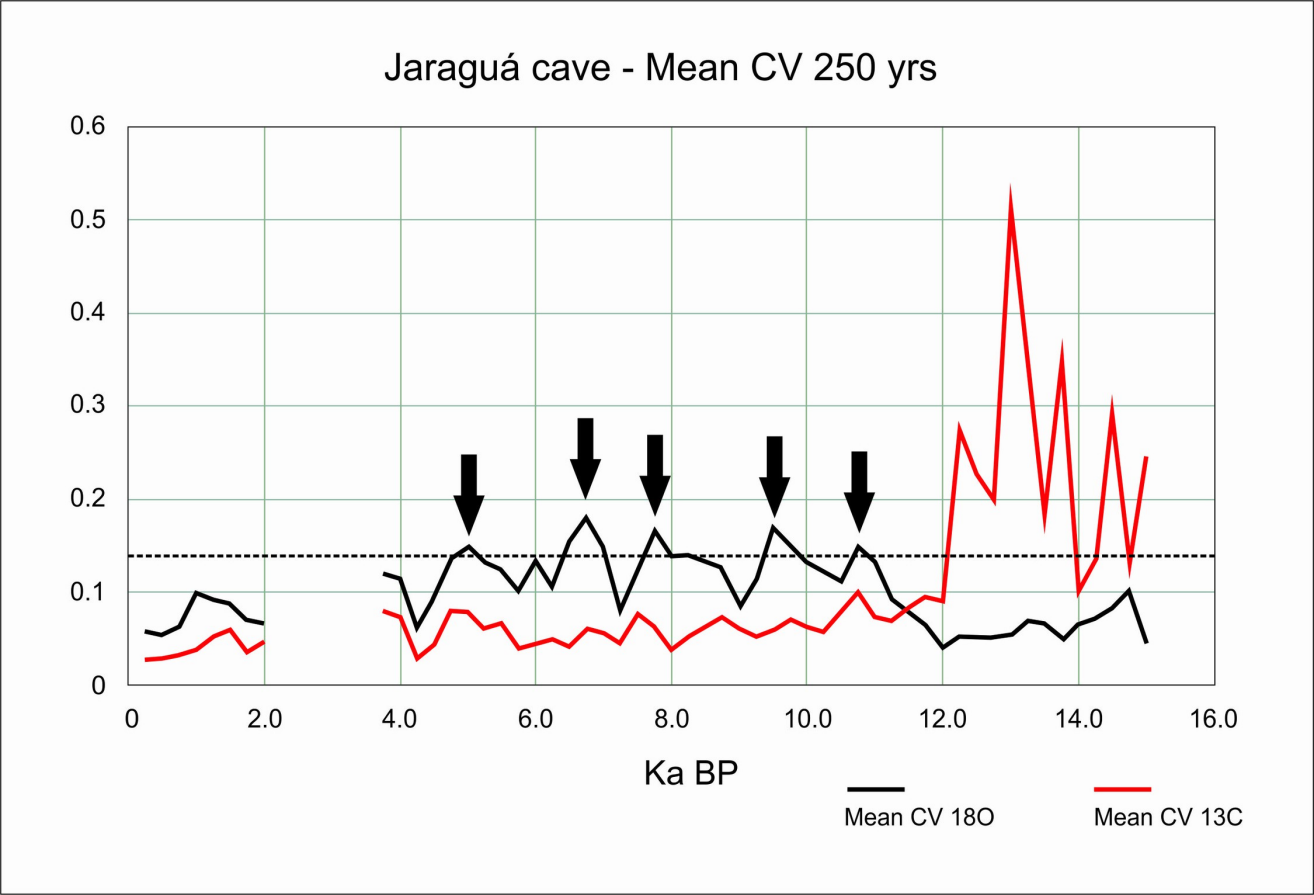
Jaraguá cave. Mean coefficients of variation (CVs) in 250 years slots for delta ^18^O and delta ^13^C (after Novello et al. 2019). Dashed line marks the mean plus one standard deviation value (0.14) for delta ^18^O. Intervals above it are interpreted as extreme variations (black arrows).

When we proceed to focus on variability inside the proxies, it is also possible to observe a major discrepancy between the 250-yr mean CVs of ^18^O and ^13^C, especially in the late Pleistocene, where strong variations observed in the ^13^C are not present in the ^18^O data (Fig 22). When we examine the ^18^O mean CVs (mean = 0.098; std dev = 0.044), it is possible to perceive that the peaks that surpass one standard deviation (0.14) fall ca. 10.75 ka BP, 9.5 ka BP, 7.75 ka BP, 6.75 ka BP, and 5 ka BP. This is also in very good agreement with the age curve for Mato Grosso do Sul (Fig 18D).

**Fig 23:**
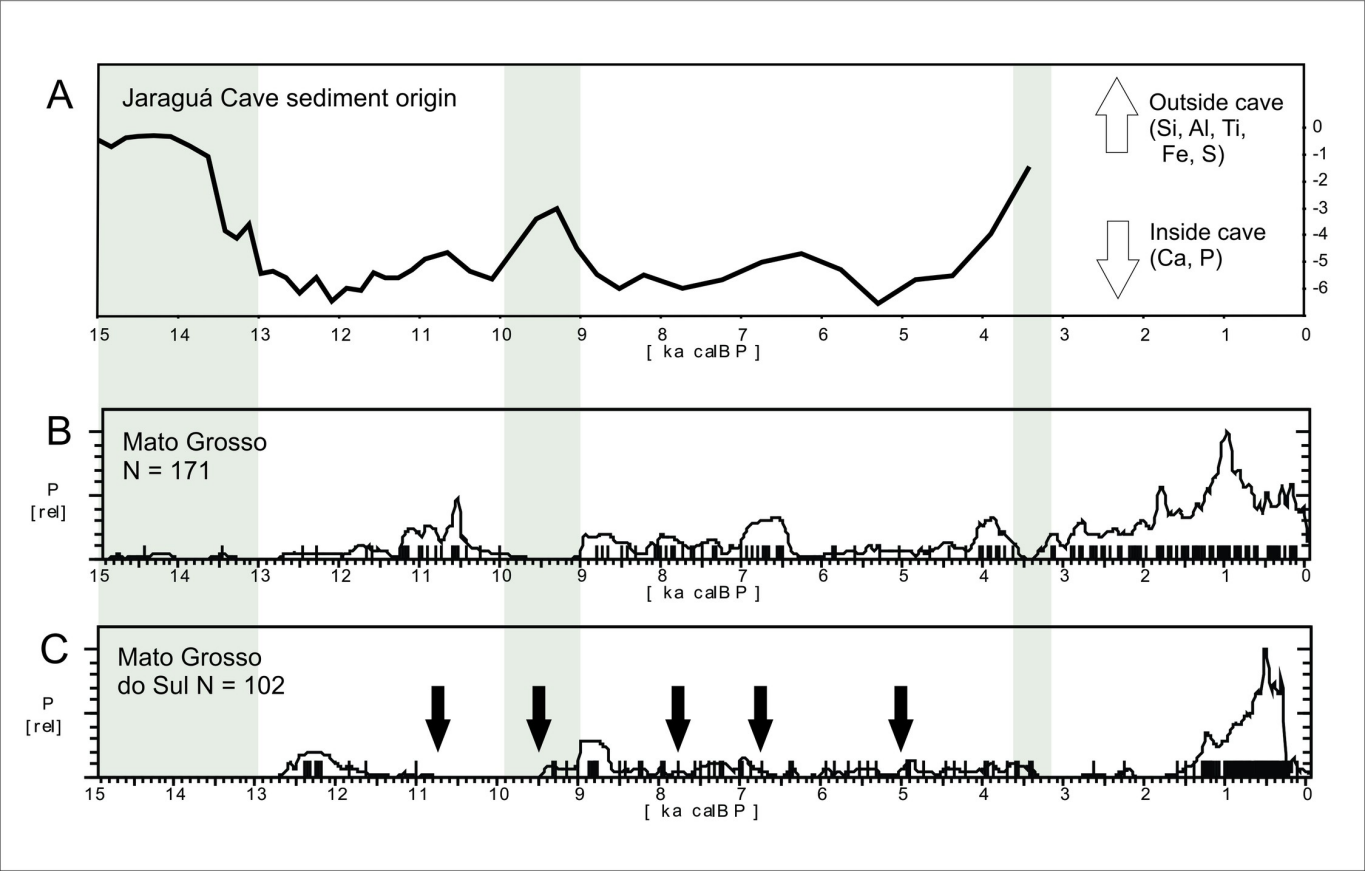
A) Data from Jaraguá cave (Novello et al. 2019) showing the different natures of sediment input (outside x inside cave) until 3.5 ka BP. Sediments coming from outside are probably signaling disturbances in the vegetational cover (rhesistasy); B) Summed probability age graph for Mato Grosso; C) Summed probability age graph for Mato Grosso do Sul. The shaded bars highlight the match between rhesistatic conditions and the diminution of the archaeological signal. Black arrows indicate the periods where delta ^18^O shows extreme variability.

The nature of sediment input at Jaraguá cave is also of interest, since it can be linked to disturbances in soil cover and, ultimately, to rhesistatic periods. The stronger presence of minerals related to the outside soil (Si, Al, Ti, Fe, S) mark such disturbances, in contrast with minerals that are formed inside the cave (Ca, P–[149]). Fig 23A shows this relationship, again pointing towards the relation between stronger disturbances and absence of the archaeological signal. Both the 8.2 ka and the 4.2 ka events show age depressions. It is worth mentioning that ca. 9.5 ka BP there seems to be a strong environmental disturbance, marked by the mean ^18^O CV curve, the peak of soil input, and the absence of archaeological signal.

Lastly, Parolin and Stevaux [147] found evidence of a strong arid episode with dune formation at Taquarassu ca. 3 ka BP, which coincides with an absence of archaeological ages in Mato Grosso do Sul (Fig 18D).

**Fig 24:**
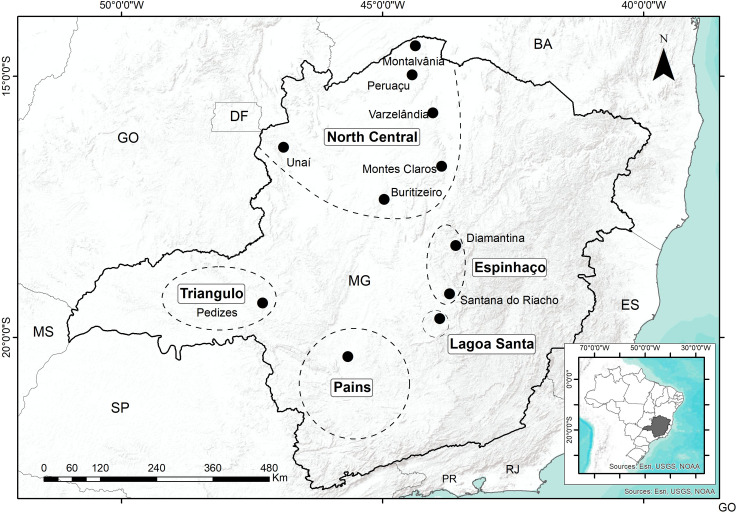
Eastern Central Brazil (Minas Gerais state) and the five regions discussed in the text (North Central, Espinhaço, Lagoa Santa, Pains, and Triângulo).

**Fig 25:**
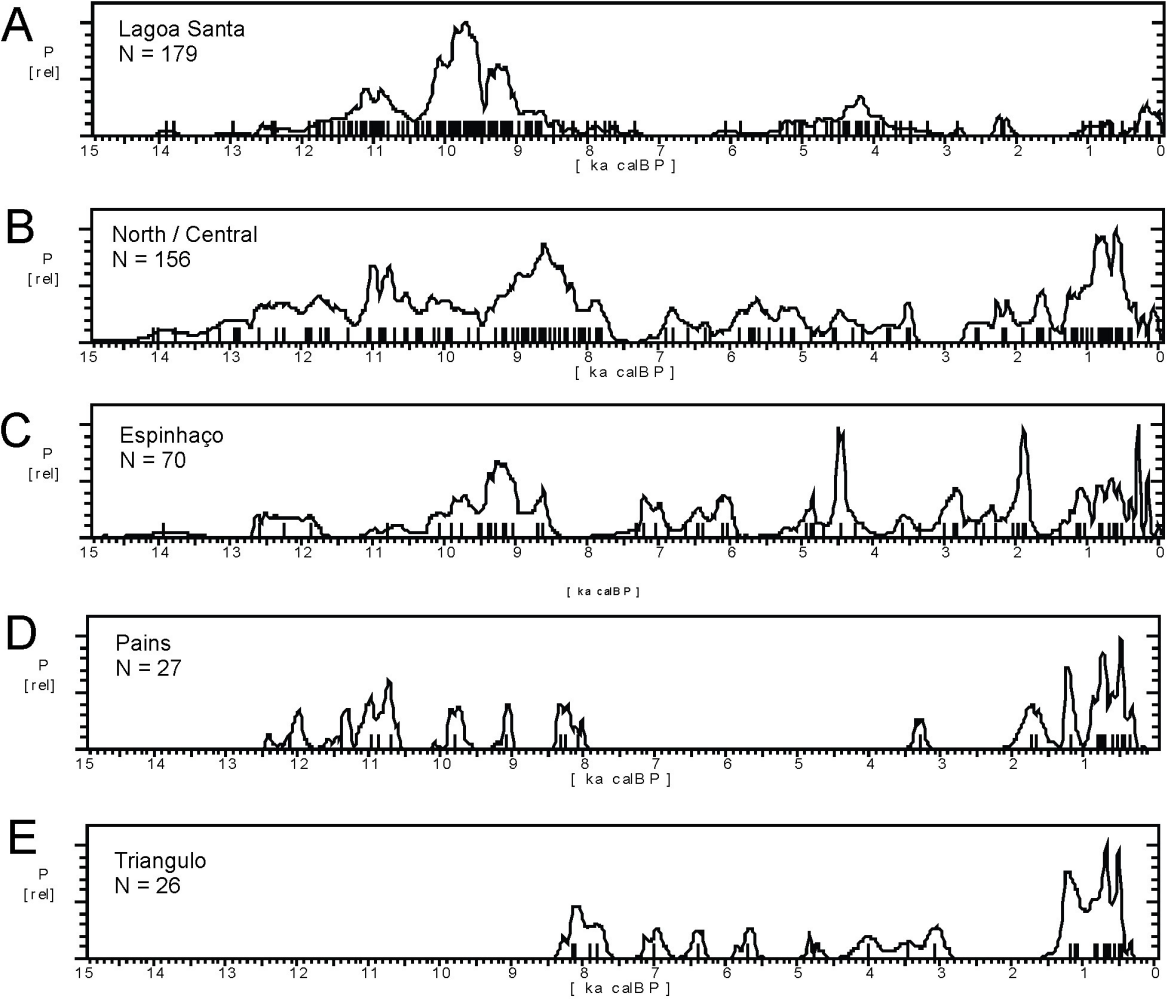
Summed probability distribution graphs for A) Lagoa Santa; B) North/Central MG; C) Espinhaço; D) Pains; E) Triangulo.

*Eastern Central Brazil*. This area comprises Minas Gerais State (MG–Fig 4) and contains a wealth of both archaeological and paleoenvironmental data (Figs 2 and 3). The northern portion of MG is more related to the Nordeste in terms of climate and vegetation, with a very marked and extended dry season, resulting in a scrub savannah, whereas the southern portion is more vegetated, and the western portion was covered with rainforest. The Espinhaço mountain range spreads in a north / south orientation and constitutes a limit between the western rainforest and the eastern savannahs. Based on the age patterns, we subdivided the region into five areas: North/ Central, Lagoa Santa, Espinhaço, Triangulo, and Pains (Fig 24). Fig 25 shows the age patterns for these five areas.

Lagoa Santa and North/Central MG areas (Fig 25A and 25B) show a more robust database, resulting from intense research and a greater investment in dating. It is readily perceived that the patterns are overall very different, especially between Lagoa Santa and Espinhaço (U = 4216; p = 0.0001), but also between the other areas. A simple inspection suggests a population movement from north to south in the late Pleistocene, at least since 14 ka BP, when the archaeological signal is steadily increasing in the North/Central area but faint at Lagoa Santa until 12 ka BP. There are some antiphased periods between these two areas, such as the increase in the archaeological signal at North/Central after 9 ka BP, when Lagoa Santa starts to become depopulated. Another instance occurs between 7.0 and 5.5 ka BP, when Lagoa Santa shows almost no sign of human occupation, while North/Central shows the opposite. In common, both areas show a sharp depletion in the signal ca. 9.5 ka BP. This event marks the onset of a strong archaeological signal at Espinhaço. Later, Espinhaço shows an antiphased pattern in relation to Lagoa Santa between 6 ka BP and 7 ka BP, and also in the last 4 ka BP. Given the similarities in the lithic industry found at Lagoa Santa and Espinhaço [155, 156], it is possible that this antiphased pattern is related to a population movement between the two areas. Pains, towards south, shows an antiphased pattern in relation to both Lagoa Santa and Triangulo since 8.5 ka BP. The population crash at the arrival of the Europeans (ca. 500 BP) is observable in all regions, except for Lagoa Santa, which seemed to be a refugium for the Aratu ceramist groups.

Paleoenvironmental data for Lagoa Santa was carried out by different authors. Parizzi et al. [157] obtained a core at Lagoa Santa lake (Fig 3, number 48) where only the upper 120 cm showed palynomorphs, and the chronology was based on two ^14^C ages. The interpretation was that “the lake did not exist prior to 5300 [6.1 ka BP] years ago and the climate was very dry” ([157]:315). About 5.3 ka BP (or 4600 ^14^C BP, interpolated) “the intermithtent marsh was replaced by a small, shallow lake that occupied part of the valley” ([157]:317). In spite of possible problems related to the age interpolations, this interpretation suggests a good match with the onset of reoccupation of Lagoa Santa at 5.3 ka BP shown in Fig 25 A. More recently, another team [123, 158] worked at two nearby lakes in the region, Mares and Olhos (Fig 3, number 56 and 47), with the explicit aim of testing the “aridity” hypothesis (which was not the term originally used by Araujo et al. [22]) regarding the abandonment of Lagoa Santa region by humans, this time with a much better chronological control. The main conclusion was that “the abrupt changes in fern and algal representation were probably best interpreted as responding to oscillations in humidity and lake level, and also supported the idea of increased climatic instability during the Archaic Gap” ([123]:148). It is important to note that at Olhos lake, wich has a much better chronological control comprising 15 ages, there is a marked interval with age inversions ca. 7.5 to 7.8 ka BP (op. cit: 146), suggesting very low sedimentation rates and events of lake drought. Once more, this matches well the onset of depopulation shown at Fig 25A. Another important lesson from these three neighbouring lakes is that there is no good match *between* them: the interpretation of a dry early Holocene for Santa lake does not match Olhos lake, only 2 km away, and the mixed forest taxa present at Olhos lake covers a period from 12.6 ka BP to 5.5 ka BP, while at Mares, only 8 km away, the forest is present between 15 ka BP and 9.6 ka BP. Again, this suggests the role of fluctuating environments in confusing signals, and the problems of extrapolating specific interpretations over wide areas, especially during periods of high climatic variability.

Paleoenvironmental studies for North / Central MG also comprise speleothems and lake cores [150, 159–161].

Cassino and Meyer [159] presented a pollen study for Laçador swamp (Fig 3, number 62). Despite the low resolution of the core (13 ka BP compressed in 1 m), it is possible to perceive at ca. 7.8 ka BP the co-occurrence in high pollen concentrations of taxa pertaining to different phytophysiognomies (Zone LAÇ3, sample R11, depth 0.61 m; ([159]:134) such as arboreal Cerrado, grasslands, herbaceous/scrub Cerrado, and palm swamp, suggesting a fluctuating environment. This shows a good match with the onset of a hiatus in the age curve (Fig 25B). Moreover, after ca. 3 ka BP (Zone LAÇ4B, sample R15, depth 0.33m; [159]:134) the authors also found pollen of Caatinga (warm, semi-arid) species together with species related to cold and humid (montane forest) and arboreal Cerrado, again suggesting strong climate variability with a good match with a second hiatus observed in Fig 25B.

Sabino et al. [161] presented another pollen analysis for Pandeiros swamp (Fig 3, number 50), 250 km NE from Laçador swamp. The 1.3 m core shows a chronology going back to 4.1 ka BP, and the authors observed that between ca. 4.1 ka BP and 3.7 ka BP there is an increase in organic matter and a decrease of sand fraction, suggesting low rates of slope erosion and a lentic environment surrounded by palm trees ([161]:1032). This period is in contrast with the 3.6 ka BP to 3.1 ka BP interval, when there is the disappearance of palm trees and a somewhat conflicting scenario where indicators of flooded environments appear together with species related to dry forest. Conditions seem to become even less favourable between 2.9 ka BP and 2.8 ka BP, when the marshland disappears, the dry forest decreases and the grassland expands ([161]:1033). This scenario matches very well the abovementioned second hiatus in archaeological ages (Fig 25B), especially when the authors observe that ca. 2.6 ka BP palm swamp taxa return, indicating a more stable and humid climate, which marks the end of the hiatus.

**Fig 26:**
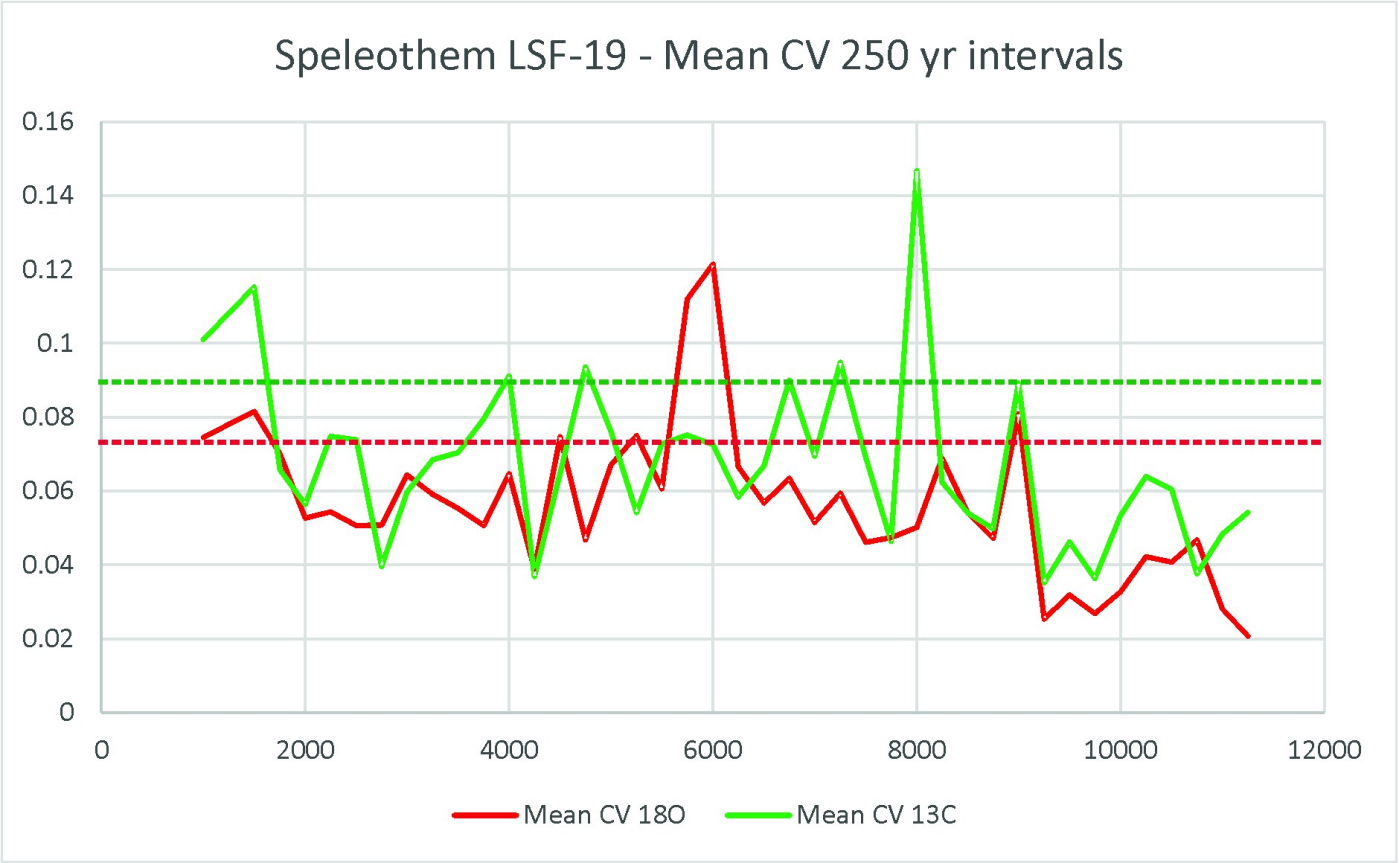
Lapa sem Fim speletothem data. Mean CVs averaged at 250 yr intervals for delta ^13^C (green line, proxy for vegetation) and delta ^18^O (red line, proxy for precipitation). Dashed lines show the value of the mean plus one standard deviation for each proxy. Note the peak of vegetation variability ca. 8 ka BP, not followed by precipitation. The opposite trend occurs at ca. 6 to 5.75 ka BP, when a high variability on precipitation is not followed by a vegetational response.

Stalagmites from two caves were studied [150] (Lapa Grande and Lapa Sem Fim, Fig 3, number 49 and 58). Contrary to what happened at Jaraguá cave, the Lapa sem Fim speleothem record shows a moderate positive correlation between delta ^18^O and delta ^13^C (Spearman r = 0.138; N = 692), although some peaks of extreme variability in the CVs of delta ^13^C and delta ^18^O do not match. Fig 26 compares the CVs of the two proxies, and it is possible to observe a marked peak of vegetation variability at ca. 8 ka BP, not followed by a similar variability in precipitation. The contrary occurs at ca. 6 ka BP, when a peak in precipitation variability apparently did not promote a variability in vegetation. It is important to note that there is a hiatus in the speleothem growth between ca. 8.1 and 7.6 ka BP, whose match with the hiatus in the radiocarbon ages between ca. 7.8 to 7 ka BP is not perfect but deserves attention (Fig 25B). It seems that, at least in this case, ^13^C seems a better proxy for environmental disturbances than ^18^O, since the archaeological signal seems more in accordance with the ^13^C curve.

The Espinhaço mountain range region has been studied by several authors from the paleoenvironmental point of view [162–165].

**Fig 27:**
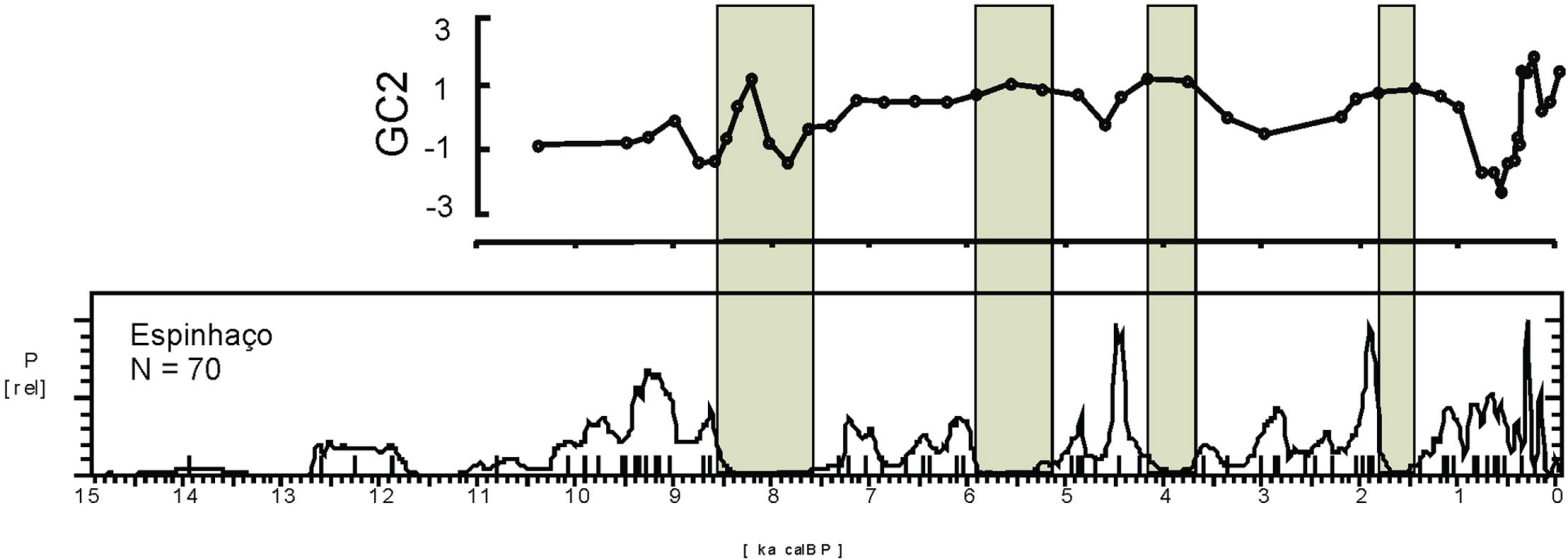
Pau de Fruta peat bog. Upper graph showing one of the factor scores from the PCA of geochemical analysis (GC2) which is related to the input of regional dust (modified from Horák-Terra et al. 2015). Negative scores indicate low contribution of regional dust. Note the sharp peak marking the 8.2 ka event and the overall good match between higher scores (probably related to rhesistatic conditions) and gaps in the archaeological signal.

Several authors [166–168] carried out analyses at Pau de Fruta peat bog (Fig 3, number 52), finding six main phases of paleoenvironmental change. Of interest here is the large instability associated to the 8.2 ka event, which marks a major hiatus in the archaeological ages (Fig 25C) and the statistical analysis (PCA) of the geochemical data, showing a very good match between the increase in the regional dust, which would be related to environmental instability (rhesistasy) and the absence of archaeological signal (Fig 27). Both the 8.2 ka event and the 4.2 ka event can be invoked in this case.

Pires et al. [164] conducted a pollen analysis of a swamp located 80 km NW of Diamantina (Juquinha swamp, Fig 3, number 59) in a 4 m core reaching 18.5 ka BP. There are only three radiocarbon ages for the profile, what somewhat hampers fine-grained interpretations, but the authors recognized a humid and colder interval between 18.5 ka BP and 8.2 ka BP, followed by a “dryer interval” between 8.3 ka BP and 7.8 ka BP. This interval shows two characteristics that could be interpreted as signals of strong climatic variability, probably related to the 8.2 ka event: a sandy layer in the middle of finer sediments, and a diminution of taxon diversity ([164]:104). From the archaeological point of view, there is a very good match given the absence of archaeological sites dated from the 8.3 to 7.8 ka BP interval (Fig 25C). The core shows other instances of deposition of coarse to medium sand across the profile with diminution of taxa ([164]:103) that could be interpreted as events of rhesistasy, but the age interpolations would not be very reliable.

Rocha-Campos et al. [165] studied another peat bog at Serra da Doida (Fig 3, number 53), 19 km West of Pau de Fruta, and found a layer of sand inside the peat accumulation sequence bracketed between 12.4 ka BP and 7.9 ka BP. According to the authors, this would be a signal of “torrential rains concentrated in few months, causing strong degradation of the hillsides”. It is also possible that such deposition is related to the 8.2 ka event observed both at Pau de Fruta and Juquinha, and not to a continuous record of instability spanning 4500 years.

Chueng et al. [169] presented a study using siliceous bioindicators (phytoliths and sponge spicules) in the region comprising three areas, but the chronology is short, barely reaching 6 ka BP, and the results pointed to “no major changes in the vegetation types” ([169]:1). A similar conclusion was given by Costa [170] and Machado et al. [171] at Rio Preto swamp (Fig 3, number 54), also using siliceous bioindicators, in spite of a larger chronological interval, ranging from 7 ka BP to 23 ka BP: “during the last 23,330 cal years BP, there has been no change in vegetation cover, only tree cover and palm density oscillations” ([171]:10). This coring site is located 40 km W of Diamantina and at a much higher elevation (ca. 1600 m a.s.l.), which could impart harsher environmental conditions without much room for vegetational changes. The other paleoenvironmental studies, as well the archaeological sites, are located up to 1300 m a.s.l.

In contrast to the areas presented before, the Pains region has virtually no paleoenvironmental data. The nearest study site was located 150 km towards South (Machado soil profile, Fig 3, number 55; [172]) and shows signs of considerable environmental change around 6 ka BP, when humidity appears to increase sharply. However, we will have to wait until a better paleoenvironmental scenario is available for this area.

The Triangulo region has several paleoenvironmental studies, the majority concentrated in the same location (Salitre ‐ Fig 3, number 60 and 64; [173–176]) and another at Serra Negra lake [177] (Fig 3, number 63). Palynological data presented by Ledru [174] at Salitre suggests a fluctuating early Holocene climate, with low pollen frequencies ca. 11.8 ka BP, with conditions “not favourable for forest development, probably because of a prolonged dry winter season combined with low temperatures” ([174]:95). By 10.4 ka BP there is a “sudden increase in arboreal taxa” and high frequency of *Araucaria*, meaning moister and colder climate, without a dry season. Around 8.9 ka BP the *Araucaria* forest is replaced by mesophytic semideciduous forest, meaning an increase in dry season. Between 6.4 ka BP and 5 ka BP the pollen frequency becomes very low, with evidence of strong moisture stress. Vernet et al. [176] recorded soil charcoal in order to infer paleoenvironmental conditions at Salitre and proposed a good match between the charcoal signal and Ledru’s [174] data. However, carbon isotopes signatures from soil profiles at Salitre studied by Pessenda et al. [175] did not reach the same conclusions: “our 13C data from charcoal and SOM samples suggest that C4 grasses were not the predominant vegetation at any time in the SOM record, including the drier periods postulated by Ledru (1993)” ([175]:199).

The Serra Negra lake record [177] is long, reaching 39 ka BP, but the last 18,000 years are compressed in the upper 1.6 m. The author stated that “since 5000 years BP [5.7 ka BP] the climate of Serra Negra has been characterized by reduced precipitation, and higher temperatures, allowing it to support cerrado and semideciduous forests in a two season climate” ([177]:83). This is in accordance with the mid-Holocene dry phase postulated by Ledru [174].

According to Fig 25E, the onset of the human occupation signal would start at ca. 8 ka BP, and the only visible gap in ages occurs between 2.8 ka BP and 1.6 ka BP. There seems to be no good match among any of the records (pollen, soils, archaeology) up to this moment, probably due to the small sample size of archaeological ages and the lack of more detailed paleoenvironmental studies in the region.

#### Main observed paleoenvironmental and archaeological trends for Central Brazil

Most depressions in the age SPD curves for Central Brazil show a good correlation with RCCs (Table 4), the only exceptions being a period between 2.6 to 2.3 ka in Pantanal, and 7.9 to 5.6 ka in Goiás. In this last case, the large interval could be the result of the several RCCs in sequence (from the 8.2 ka event until the 5.5 ka event). A visible trough in the SPD curves around 9.5 ka, present at Lagoa Santa, North/Central, Pains and Mato Grosso regions, also visible at the Jaraguá cave isotope data, seems well correlated to the 9.2 ka event that was described for the archaeological record of the Levant and Europe [38, 178]. The 8.2 ka event is also well represented in several records, as is the case of the 4.2 ka event. It is also worth noting that the Roman Warm Period [179] (2.0 to 1.3 ka) is well marked in Lagoa Santa, but less recognizable in the other regions.

**Table 4:** Paleoenvironmental and archaeological data for Central Brazil.

Author	Area / region	Instability Age Ka	Evidence	RCC relation
Whitney et al. 2014	Pantanal	9.0 to 7.8	Decrease plant taxa	Yes ‐ 9 to 8 ka Glacial Aftermath [6]
This paper	Pantanal	9.0 to 6.5	Age SPD depletion	Yes ‐ 9 to 8 ka Glacial Aftermath [6] + 6.4 ka event [97]
		6.0 to 5.5		
		4.1 to 3.5		Yes ‐ 5.5 ka event [95]
		2.6 to 2.3		Yes ‐ 4.2 ka [91] + 3.8 to 3.1 ka event [96]
		2.0 to 1.8		Unclear
				Yes ‐ RWP [179]
McGlue et al. 2012;	Pantanal	9.0 to 2.5	Contradictory paleoenvironmental signals	Unclear ‐ probably several RCCs averaged
Bezerra & Mozeto 2008;				
Rasbold et al. 2019; Sallun Filho et al. 2009				
Ferraz Vicentini 1999; Turcq et al. 2002	Feia lake	9.7	Low TOC	Yes ‐ Meltwater pulse 1C [8]
		5.8 to 5.7	High charcoal imput	
		4.3 to 4.1		Yes ‐ 5.5 ka event [95]
		3.4 to 3.1		Yes ‐ 4.2 ka event [91]
				Yes ‐ 3.8 to 3.1 ka event [96]
Cassino et al. 2020	Feia lake	10.5	Sterile layers	Yes ‐ Lake Agassiz outburst [9]
		9.1 to 8.8	Peaks in Ti and Fe	
		8.0 to 6.8		Yes ‐ 9 to 8 ka Glacial Aftermath [6]
		6.4		
				Unclear ‐ probably several RCCs averaged
				Yes ‐ 6.4 ka event [97]
This paper	Goiás	9.1 to 8.4	Age SPD depletion	Yes ‐ 9 to 8 ka Glacial Aftermath [6] Unclear ‐ 6.4 ka event?
		7.9 to 5.6		
		4.4 to 3.4		
				Yes ‐ 4.2 ka event [91]
		2.0 to 1.3		Yes ‐ RWP [179]
This paper	Mato Grosso	11.3	Age SPD depletion	Yes ‐ Meltwater pulse 1B [8]
		9.7 to 9.0		
		6.3 to 6.0		
		ca. 3.4		
		ca. 1.5		Yes ‐ 9.2 ka event [38]; [178]
				Yes ‐ 6.4 ka event [97]
				Yes ‐ 3.6 ka event [12]
				Yes ‐ 1.4 ka event [97]
This paper	Mato Grosso Sul	10.8 to 9.5	Age SPD depletion	Yes ‐ 10.5 ka event [42]
		6.3 to 6.0		Yes ‐ 6.4 ka event [97]
		ca. 3.5		Yes ‐ 3.6 ka event [12]
		2.1 to 1.7		Yes ‐ 2.1 ka event [12]
Novello et al. 2006	Pau d´Alho cave	1.2 to 1.0	Fluctuating isotope data	Yes ‐ 1.0 ka warm excursion [250]
This paper	Jaraguá cave	10.8	Fluctuating delta ^18^O isotope data	Yes ‐ 10.5 ka event [42]
		9.5		Yes ‐ 9.2 ka event [38]; [178]
		7.8		
		6.8		Unclear
		5.0		Yes ‐ 6.4 ka event [97]
				Yes ‐ 5.5 ka event [95]
Novello et al. 2019	Jaraguá cave	10.0 to 9.0	Increase in external minerals	Unclear ‐ Meltwater pulse 1C + 9.2 ka event?
		3.6 to 3.3		
				Yes ‐ 3.8 to 3.1 ka event [96]
Parolin & Stevaux 2001	Taquarassu	ca. 3.0	Dune formation	Yes ‐ 3.0 to 2.3 ka event [96]
This paper	Lagoa Santa	ca. 10.5	Age SPD depletion	Yes ‐ 10.5 ka event [42]
		ca. 9.5		Yes ‐ 9.2 ka event [38]; [178]
		ca. 8.2		
		7.5 to 6.3		Yes ‐ 8.2 ka event [93]
				Yes ‐ 7.6 to 7.0 ka [41] + 6.4 ka event [97]
		2.7 to 2.4		
		2.0 to 1.3		Yes ‐ 3.0 to 2.3 ka event [96]
				Yes ‐ RWP [179]
Raczka et al. 2013	Lagoa Santa	7.8 to 7.5	Lake drought	Yes ‐ 7.6 to 7.0 ka event [41]
This paper	North/Central MG	7.8 to 7.0	Age SPD depletion	Yes ‐ 7.6 to 7.0 ka event [41]
		3.5 to 2.8		
				Yes ‐ 3.8 to 3.1 ka event [96]
This paper	Espinhaço	11.7 to 11.2	Age SPD depletion	Yes ‐ Lake Agassiz outbursts [9]
		8.4 to 7.5		
		5.9 to 5.3		Yes ‐ 8.2 ka event [93]; 7.6 to 7.0 ka event [41]
		ca. 4 ka		Yes ‐ 5.5 ka event [95]
				Yes ‐ 4.2 ka event [91]
		1.7 to 1.5		
				Yes ‐ 1.6 ka climate excursion [250]
This paper	Pains	10.5 to 10.1	Age SPD depletion	Yes ‐ 10.5 ka event [42]
		9.5 to 9.3		Yes ‐ 9.2 ka event [38]; [178]
		8 to 3.5		
		3.0 to 2.0		Unclear ‐ probably several RCCs averaged
		ca. 1.4		
		ca. 1.0		Yes ‐ 3.0 to 2.3 ka event [96]
				Yes ‐ 1.4 ka event [97]
				Yes ‐ 1.0 ka warm excursion [250]
This paper	Triangulo	2.8 to 1.6	Age SPD depletion	Yes ‐ 2.8 ka event [94] + RWP [179]
Cassino & Meyer 2013	Laçador swamp	ca. 7.8	Contradictory pollen signals	Unclear ‐ 7.6 to 7.0 ka event?
		ca. 3.0		
				Unclear ‐ 2.8 ka event?
Sabino et al. 2021	Pandeiros swamp	3.6 to 3.1	Contradictory pollen signals	Yes ‐ 3.6 ka event [12]
		2.9 to 2.8		Yes ‐ 2.8 ka event [94]
Azevedo et al 2021	Lapa sem Fim cave	ca. 8.0	Isotopic variability	Yes ‐ 8.2 ka event [93]
		ca. 6.0		Yes ‐ 6.4 ka event [97]
Horák-Terra et al. 2015; Luz et al. 2017; Schellekens et al. 2014	Pau de Fruta peat bog	8.4 to 7.6	Regional dust input	Yes ‐ 8.2 ka event [93]
		5.9 to 5.3		Yes ‐ 5.5 ka event [95]+ 5.3 ka event [97]
		ca. 4.0		
				Yes ‐ 4.2 ka event [91]
		1.7 to 1.5		Yes ‐ 1.6 ka climate excursion [250]
Pires et al. 2016	Juquinha swamp	8.3 to 7.8	Sediment and pollen taxa fluctuation	Yes ‐ 8.2 ka event [93]
Ledru 1993	Salitre lake	ca. 11.8	Pollen fluctuation	Yes ‐ 11.8 ka climate excursion [250]
		8.9 to 5.0		
				Unclear ‐ Glacial aftermath + 8.2 ka + 5.5 ka + 5.3 ka events?

### Southern Brazil

Here we consider Southern Brazil as São Paulo (SP), Paraná (PR), Santa Catarina (SC) and Rio Grande do Sul (RS) states (Fig 4). São Paulo state was originally an ecotone between Cerrado and Atlantic rainforest, while the southern states show mostly ecotones between rainforest and grasslands (Paraná, Santa Catarina, and Rio Grande do Sul). While rainfall tends to be high in average, temperatures in these southernmost states can reach a few Celsius degrees below zero in the winter.

#### Paleoenvironments

Paleoenvironmental studies in Southern Brazil suggested that the Last Glacial Maximum (LGM) and Late Glacial were very dry and cold, with expansion of grasslands where today a variety of forest ecosystems occur [180], although more recent work using speleothem records tends to contradict the idea of a dry LGM (Botuverá cave, [181]:2261 ‐ Fig 3, number 97). The Late Glacial, however, was probably somewhat warmer than the LGM. During the Holocene, changes toward wetter conditions started around 6000 cal BP in São Paulo, and even later in southern Brazil, around 3 ka BP [180]. This suggested very different climatic patterns from the ones reigning towards the north. A more comprehensive discussion about the paleoenvironmetal data for this region is provided in the S5 File.

#### Archaeological data

**Fig 28:**
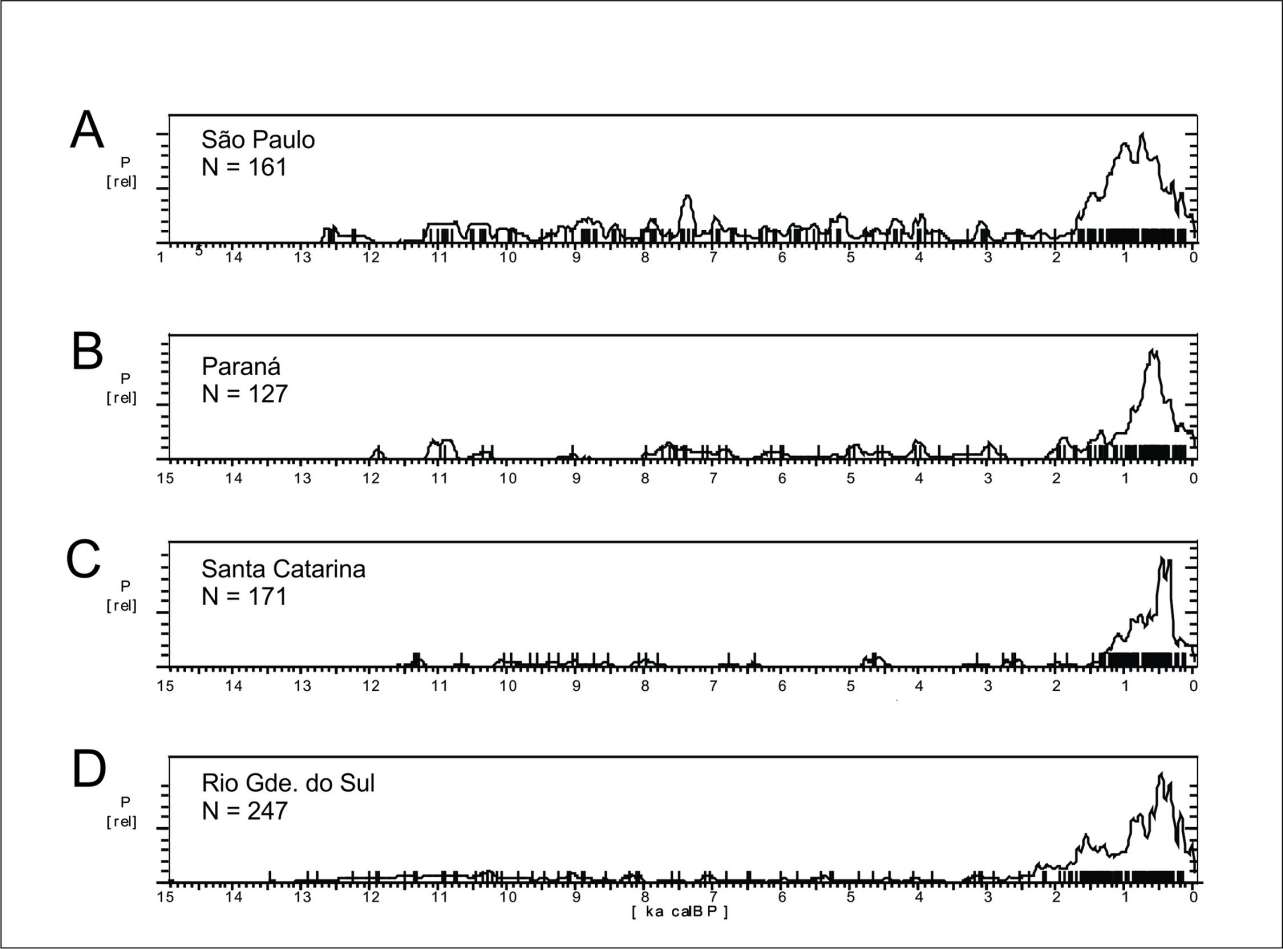
Summed probability distribution graphs for A) São Paulo; B) Paraná; C) Santa Catarina; D) Rio Grande do Sul.

The summed probability distribution graphs of the 816 ages available for Southern Brazil are shown in Fig 28. In spite of presenting the largest databases, both São Paulo (n = 242) and Rio Grande do Sul (n = 261) show no conspicuous gaps or clusters until very late, ca. 2.0 ka BP, when the curve increases sharply, probably marking the arrival and establishment of ceramist groups. The population crash following the European arrival can be observed in all graphs.

São Paulo (Fig 28A) shows a pattern suggesting an even and smooth occupation of the territory throughout the Holocene. The minor gap between 12.2 and 11.1 ka BP could be an artifact of little effort at aiming specifically the oldest human occupations in the state (most ages from the early Holocene were produced in the last years [182, 183], or alternatively a real diminution of the archaeological signal related to the YD. The 8.2 ka event, probably detected at the Paraíba do Sul valley [184], as well as near the coast [185], can also be perceived as a minor gap. A third minor depression in ages is visible ca. 3.5 ka BP to 3.2 ka BP, and can be related to a climatic shift detected by several authors [184, 186–190]. Lastly, another minor gap ca. 1.9 ka BP can be related to the “dry event” detected at Mogi Guaçu river [191].

**Fig 29:**
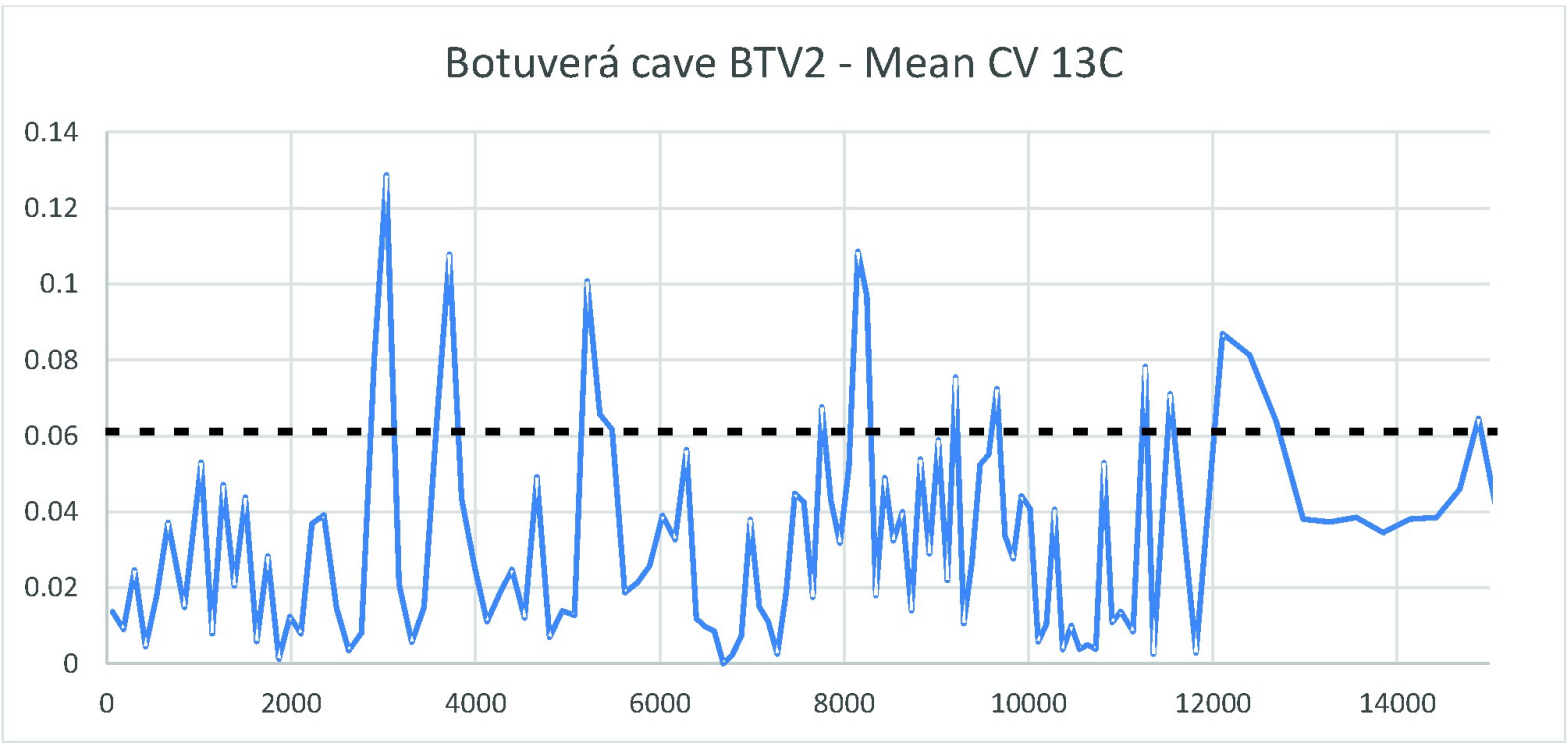
Botuverá cave BTV2 speleothem data for the last 15,000 years. Mean CVs averaged at 120 yr intervals for delta ^13^C (proxy for vegetation). Dashed line shows the value of the mean plus one standard deviation for the CV. The largest peaks of variability occur at 3.0, 3.7, 5.2, and 8.2ka BP.

**Fig 30:**
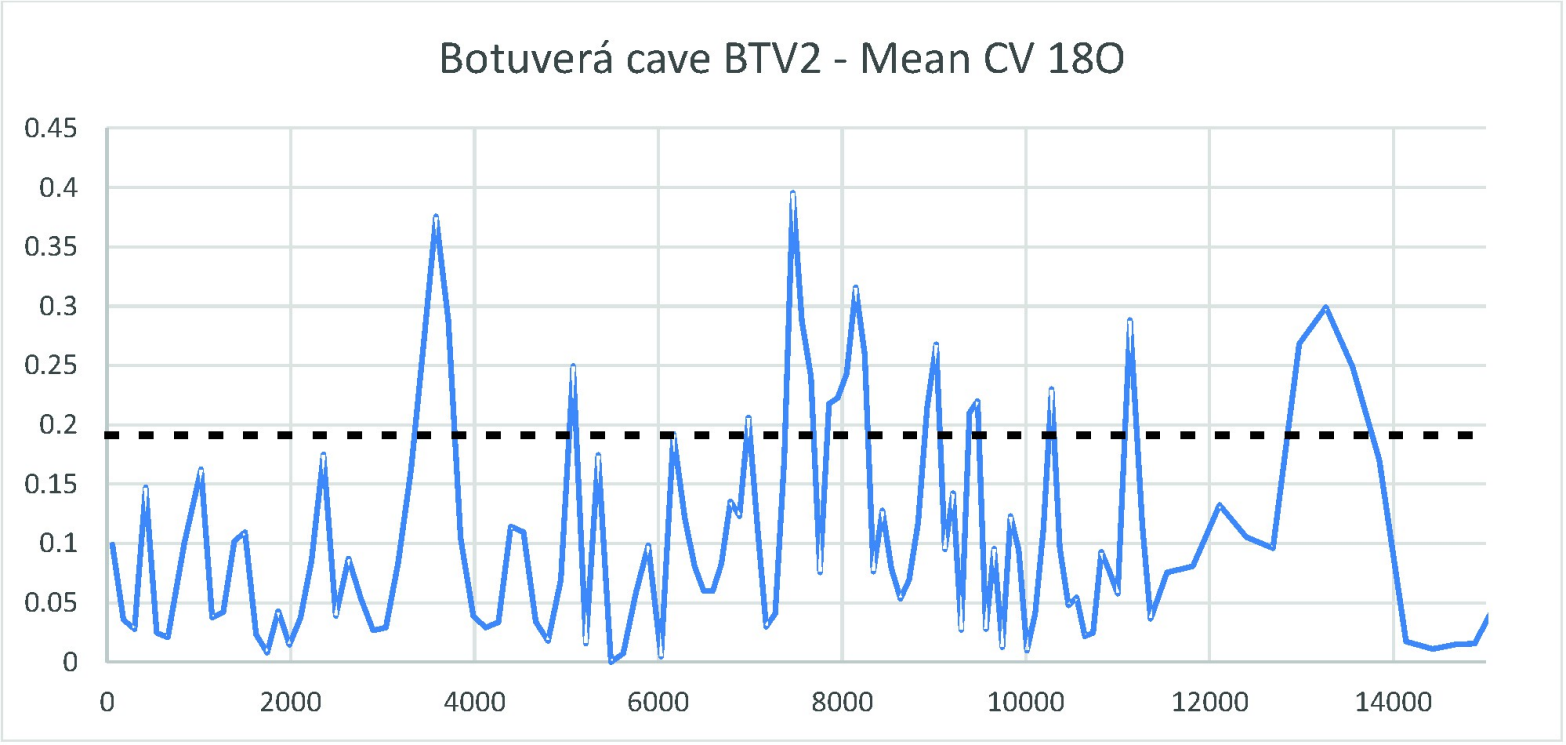
Botuverá cave BTV2 speleothem data for the last 15 ka. Mean CVs averaged at 120 yr intervals for delta ^18^O (proxy for precipitation). Dashed line shows the value of the mean plus one standard deviation for the CV. The largest peaks of variability occurr at 3.6, 7.5. and 8.2 ka BP.

The curves for Paraná (Fig 28B) and Santa Catarina (Fig 28C) show well defined gaps and seem antiphased. Periods with age peaks in Paraná represent gaps in Santa Catarina and vice-versa throughout the Holocene, which can be thought as north-south population displacements. Especially in Santa Catarina (Fig 28C), the relatively low number of ages between 7.7 and 2.0 Ka BP can be tentatively linked to the Mid-Holocene increase in instability [192]. High resolution speleothem data from Botuverá cave [193–195] show some possible explanations for the observed gaps: the correlation between delta ^18^O and delta ^13^C for speleothem BTV2 is weak but positive for the last 12,000 years (Spearman r = 0.296; N = 104) and also positively correlated with Sr/Ca ratios. All proxies pointed towards periods of increased climatic variability in the mid-Holocene. We ran the mean CVs for both ^18^O and ^13^C datasets, and the results are shown at Figs 29 and 30.

**Fig 31:**
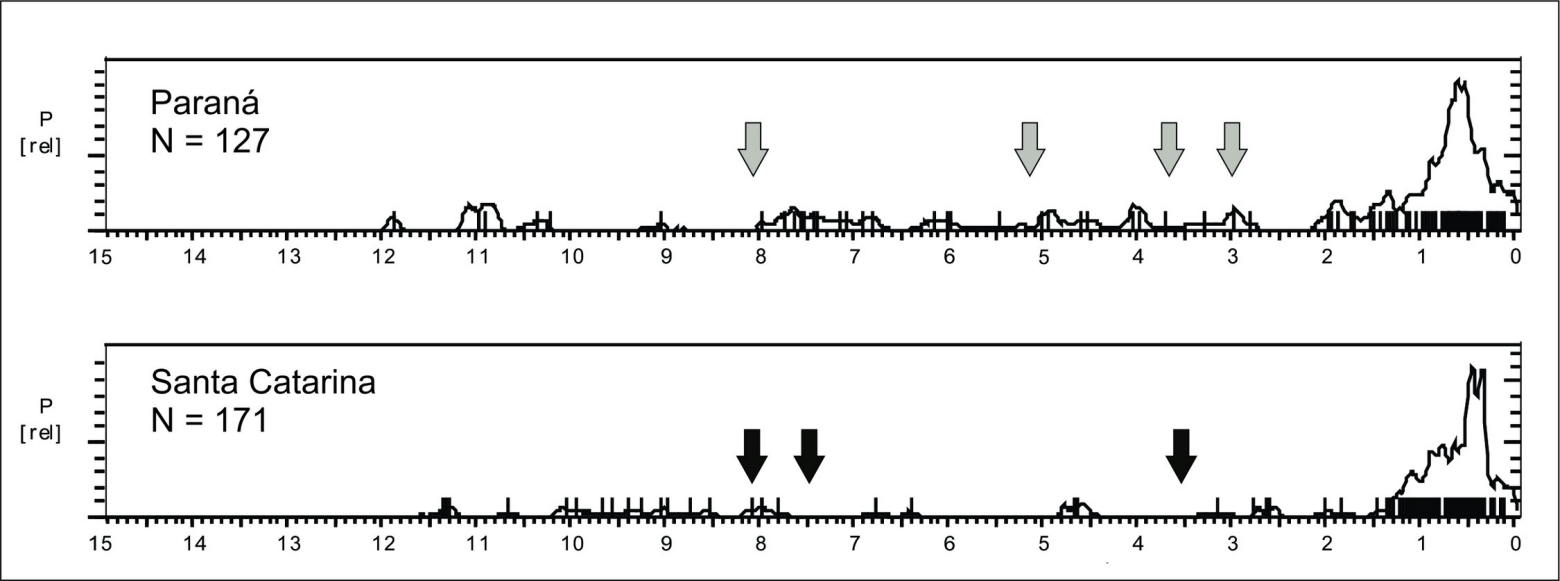
Summed probability distribution graphs for Paraná and Santa Catarina. The arrows mark events of high climatic variability calculated for Botuverá cave BTV2 speleothem. Grey arrows mark the ^13^C (proxy for vegetation) and black arrows the ^18^O (proxy for precipitation).

Albeit not perfectly coincident, the ages for the climatic variability peaks provided by the proxies should be taken into account in future studies encompassing Paraná and Santa Catarina (Fig 31). The 8.2 ka event is visible in all four curves, while the 7.5 ka BP peak shows a gap in Paraná, but an increase in ages in Santa Catarina.

The Rio Grande do Sul curve (Fig 28D) shows scattered ages during the mid to late Holocene that can be related to the same climatic pattern occurring at Santa Catarina. In fact, the datasets for both states are not significantly different (U = 19244, p = 0.0019). The climatic variability peaks of 3.6 ka BP, 5.2 ka BP and 7.5 ka BP are also coincident with gaps in Rio Grande do Sul, in spite of the larger dataset. The increase in moisture in the beginning of the Holocene [196] matches well the overall pattern of ages between 12.5 and 9.5 ka BP.

#### Main observed paleoenvironmental and archaeological trends for Southern Brazil

Even if the SPD curves do not show strong signals of population fluctuation throughout the Holocene, it was still possible to perceive some troughs that matched well the RCCs, especially the 8.2 ka event, that was also observed in the speleothem records of Botuverá cave (Table 5). The only periods where the correlation is unclear are ca. 9.6 ka for São Paulo, 6.7 to 6.4 ka and 2.7 to 2.2 ka for Paraná, and 4.4 to 3.4 for Santa Catarina. The 5.5 / 5.3 ka events are present in the southern states but not in São Paulo. The 4.2 ka event on the other hand is unclear, and seems to be present only in the Paraná SPD curve.

**Table 5:** Paleoenvironmental and archaeological data for Southern Brazil.

Author	Area / region	Instability Age Ka	Evidence	RCC relation
Behling & Safford 2010	Serra dos Órgãos	11.8 to 10.8	Pollen signal fluctuation	Yes ‐ 11.8 ka climate excursion [250]
Seixas et al. 2019	Paraíba do Sul valley	8.9 and 8.4	Stone lines	Unclear ‐ Glacial aftermath + 8.2 ka event?
Ledru et al. 2009	Colonia	6.2 to 3.7	Pollen signal	Unclear ‐ several RCCs in sequence?
Turcq et al. 1997, 2002	Tamanduá river	11.5 to 6.9	Sediment erosion	Unclear
Gadens-Marcon et al 2014	Mina Modelo pond	6.8 to 6.0	Pollen and geochemical fluctuation	Yes ‐ 6.4 ka event [97]
Leonhardt and Lorscheitter 2010	São Francisco de Paula	9.7 to 6.5	Pollen signal	Unclear ‐ several RCCs in sequence?
This paper	São Paulo state	12.0 to 11.5	Age SPD depletion	Yes ‐ 11.8 ka climate excursion [250]
		ca. 9.6		
				Unclear ‐ 9.2 ka event [38]?
		8.2		Yes ‐ 8.2 ka event [93]
		3.5 to 3.2		Yes ‐ 3.6 ka event [12]
		ca. 2.8		Yes ‐ 2.8 ka event [94]
This paper	Paraná state	11.8 to 11.2	Age SPD depletion	Yes- Lake Agassiz outbursts [9]
		10.2 to 9.3		
		8.8 to 8.0		Unclear
		6.7 to 6.4		Yes ‐ 9.0 to 8.0 Glacial aftermath [6] + 8.2 ka event
		5.8 to 5.3		Unclear
		4.4 to 4.2		
		3.8 to 3.5		Yes ‐ 5.5 ka event [95]
				Yes ‐ 4.2 ka event [91, 250]
		2.7 to 2.2		Yes ‐ 3.6 ka event
		ca. 1.6		Unclear
		ca. 1.4		Yes ‐ 1.6 cold excursion [250]
				Yes ‐ 1.4 ka event [97]
This paper	Santa Catarina state	8.5 to 8.3	Age SPD depletion	Yes ‐ 8.2 ka event [93]
		7.7 to 6.9		Yes ‐ 7.6 to 7.0 ka event [41]
		6.3 to 4.8		Yes ‐ 6.4 ka [97] + 5.5 ka [95]+ 5.3 ka events [97]
		4.4 to 3.4		
		2.5 to 2.1		Unclear ‐ 4.2 ka + 3.8 to 3.1 ka events?
		1.6		
				Yes ‐ 2.1 ka event [12]
				Yes ‐ 1.6 ka climate excursion [250]
This paper	Botuverá cave	8.2	Strong oscillation delta ^13^C	Yes ‐ 8.2 ka event [93]
		5.2		Yes ‐ 5.3 ka event [97]
		3.7		Yes ‐ 3.6 ka event [12]
		3.0		Yes ‐ 2.8 ka event [94]
This paper	Botuverá cave	8.2	Strong oscillation delta ^18^O	Yes ‐ 8.2 ka event [93]
		7.5		Yes ‐ 7.6 to 7.0 ka event [41]
		3.6		Yes ‐ 3.6 ka event [12]
This paper	Rio Grande do Sul state	ca. 8.4	Age SPD depletion	Yes- 8.2 ka event [93]
		ca. 7.5		Yes ‐ 7.6 to 7.0 ka event [41]
		5.2 to 4.9		Yes ‐ 5.3 ka event [97]
		3.6 to 3.3		Yes ‐ 3.6 ka event [12]
		ca. 2.8		Yes ‐ 2.8 ka event [94]
		ca. 1.2		Yes ‐ 1.4 ka event [97]

### The Brazilian Southern Coast

The coastal areas are known for presenting a stable source of foodstuff for both prehistoric and present populations. The relatively mild climate, with lesser temperature and precipitation amplitudes when compared to inland areas, general abundance of predictable resources, and easiness of travel along straight lines, either on foot or by boat, were recognized by several authors as reasons to believe that coastal areas were extremely important throughout human history [197] and configured major migration corridors [198–201].

Most dated sites located in the Brazilian coast during the Holocene are shellmiddens, and most are located in the Southern coast, from 19° to 30° latitude S. We will, therefore, concentrate our analysis on the Southern Brazilian coast (Fig 4) in spite of the fact that our database contains coastal sites from other regions, which can be further explored.

#### Paleoenvironments

The most conspicuous factor affecting the coastal areas, especially from an archaeological point of view, are related to changes in the mean relative sea level (MRSL), which in turn are linked to the temperature rise since the LGM and heavy input of water from melting glaciers and ice sheets [202]. The Southern Brazilian shelf was, therefore, subject to different MRSLs during the last 18,000 years, possibly reaching 120 to 130 m below the present sea level [203]. After this major drop in the MRSL, the rising occurred at different rates, with periods of very fast increase [8] intercalated with periods of stability. Corrêa [203] detected at least three periods of stability marked by submerged terraces in the continental shelf, the first at 11.0 ka BP when the MRSL was 60 to 70 m below present, the second at 9.0 ka BP with a MRSL of -32 to -45 m, and a third ca. 8.0 ka BP, with the MRSL at -20 to -25 m. More recently, Corrêa et al. [204] dated a peat deposit on the shore of Rio Grande do Sul with an age of 12.4 ka BP, at a depth of 60m. A more comprehensive discussion about the paleoenvironmetal data for this region is provided in S6 File.

#### Archaeological data

**Fig 32:**
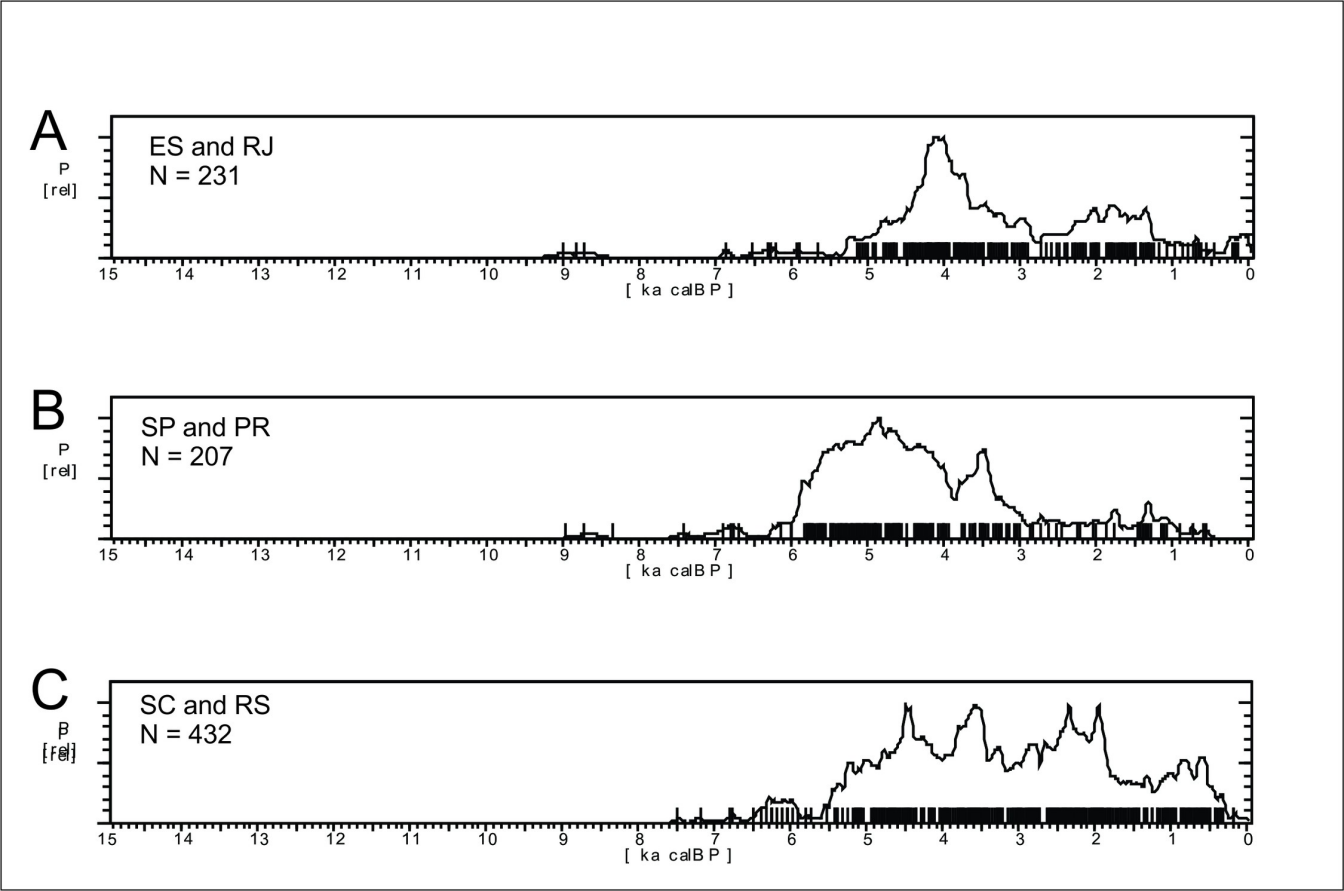
Summed probability distribution graphs for the Southern Brazil coastal areas, from North do South: A) Espirito Santo (ES) and Rio de Janeiro (RJ); B) São Paulo (SP) and Paraná (PR); C) Santa Catarina (SC) and Rio Grande do Sul (RS).

The Southern coast database was initially divided according to the state boundaries, but the Mann-Whitney U test showed no significant differences between Rio Grande do Sul and Santa Catarina (U = 4366.5, p = 0.0004), between Paraná and São Paulo (U = 4956, p = 0.812), and between Rio de Janeiro and Espírito Santo (U = 2478.0, p = 0.893), so these pairs of datasets were merged. Fig 32 presents the summed probability curves for these three regions, from North to South.

**Fig 33:**
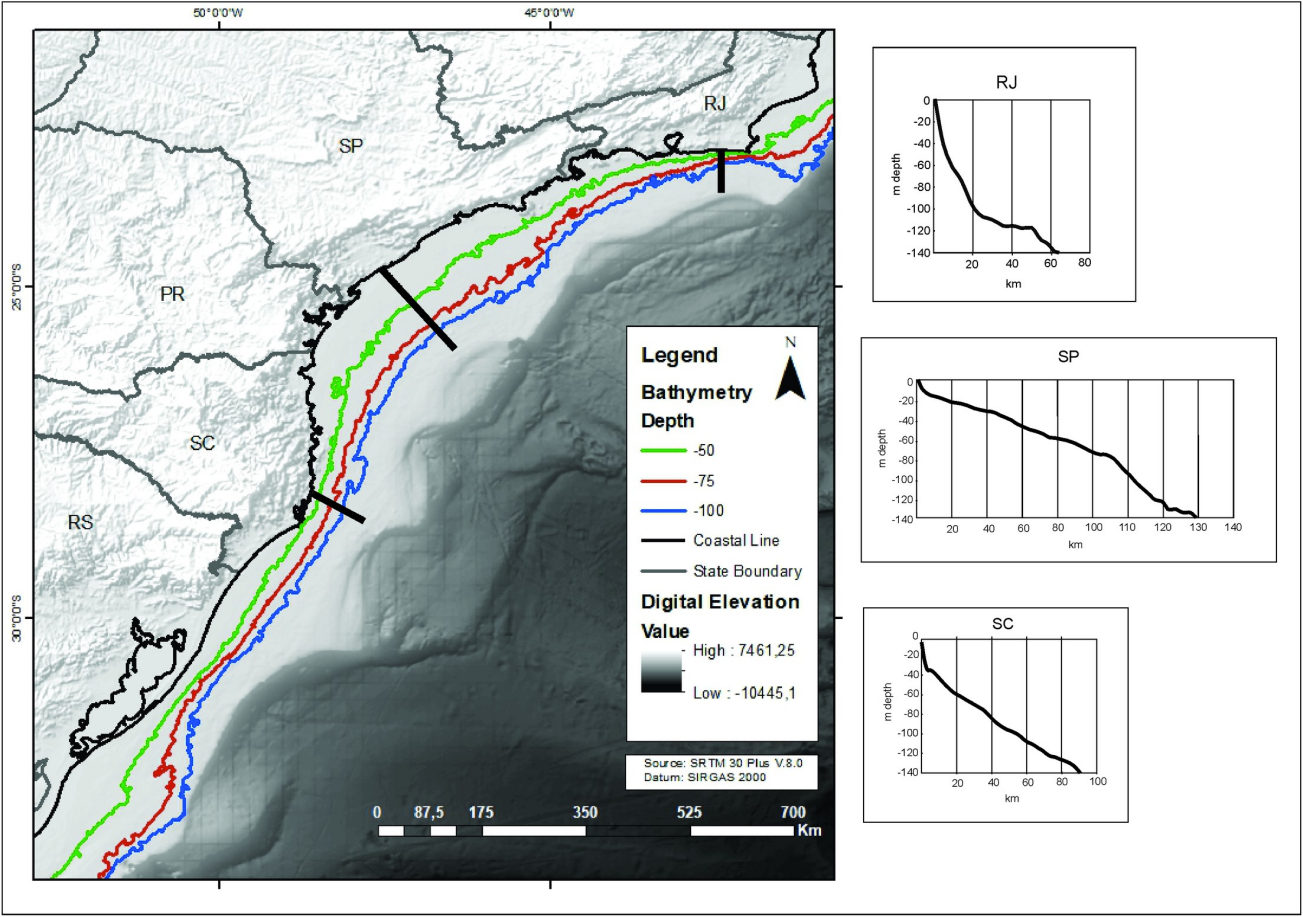
Aspect of the Southern Brazil coastal area showing the major differences in the geomorphology of the continental shelf. The black bars perpendicular to the coast represent bathymetric transects whose profiles are shown on the right. Given the same amount of sea level rise, the territorial loss would be much larger in the SP/PR coast than in the other regions.

One of the most conspicuous differences among the three regions can be perceived in the shape of the SP / PR curve, which shows a convex aspect suggesting a very strong increase in a short period of time, from 6.0 to 5.0 ka BP (Fig 32B), whereas the other two regions show a concave pattern, or a slower increase, with a time lag of some centuries. This feature is probably related to the different morphologies of the continental shelf: the SP / PR shelf is much wider and shows a less pronounced declivity in comparison to the other regions (Fig 33). As an example, we present three bathymetric profiles for RJ, SP, and SC. While the average inclination of the submerged platform at SP coast between present sea level and the isopleth of -100 m is 25°, at the coast of SC the same parameters give a number of 45°, and at RJ it reaches 70°. This means that given the same sea level rise at RJ, the amount of territorial loss at SC coast would be 32% higher, and at the SP/PR coast would be much larger, up to 122% higher, which would push the Amerindian populations inland at a faster rate, causing a domino effect and producing the observed curve.

After the steep increase in ages, the comparison between curves suggests a major drop ca. 4.0 ka BP, a second one ca. 3.0 ka BP, and a third ca. 1.3 ka BP.

#### Main observed paleoenvironmental and archaeological trends for the Brazilian Southern Coast

Given the sea level fluctuations that impart large impacts on the archaeological record, only the more recent RCCs can be eventually perceived in coastal sites. Table 6 shows the data consolidation for the paleoenvironmental and archaeological data for the Brazilian Southern coast.

**Table 6:** Paleoenvironmental and archaeological data for the Brazilian Southern coast.

Author	Area / region	Instability Age Ka	Evidence	RCC relation
Gyllencreutz et al. 2010	Southern coast	3.0 to 2.0	Increase ENSO activity	Yes ‐ 3.0 to 2.3 ka event [96] and 2.8 ka event [94]
Sallun et al. 2012	Juréia paleolagoon ‐ SP	8.2	Multiproxy analysis	Yes ‐ 8.2 ka event [93]
		ca. 4.0		Yes ‐ 4.2 ka event [91]
This paper	ES and RJ coast	2.8–2.7	Age SPD depression	Yes ‐ 2.8 ka event [94]
This paper	SP and PR coast	4.2 to 3.7	Age SPD depression	Yes ‐ 4.2 ka event [91]
Silva et al. 2021	Palhoça ‐ SC	ca. 3.0	Delta ^13^C and C/N ratios shift	Yes ‐ 2.8 ka event [94]
This paper	SC and RS coast	ca. 4.0	Age SPD depression	Yes ‐ 4.2 ka event [91]
		ca. 3.2		Unclear ‐ 3.8 to 3.1 ka event?
Barros et al. 2021	Patos lagoon ‐ RS	3.2	^13^C and ^18^O isototope oscillation	Unclear ‐ 3.8 to 3.1 ka event?
Lopes et al. 2021	Mirim lake ‐ RS	ca. 4.0	Diatom frequency	Yes ‐ 4.2 ka event

**Fig 34:**
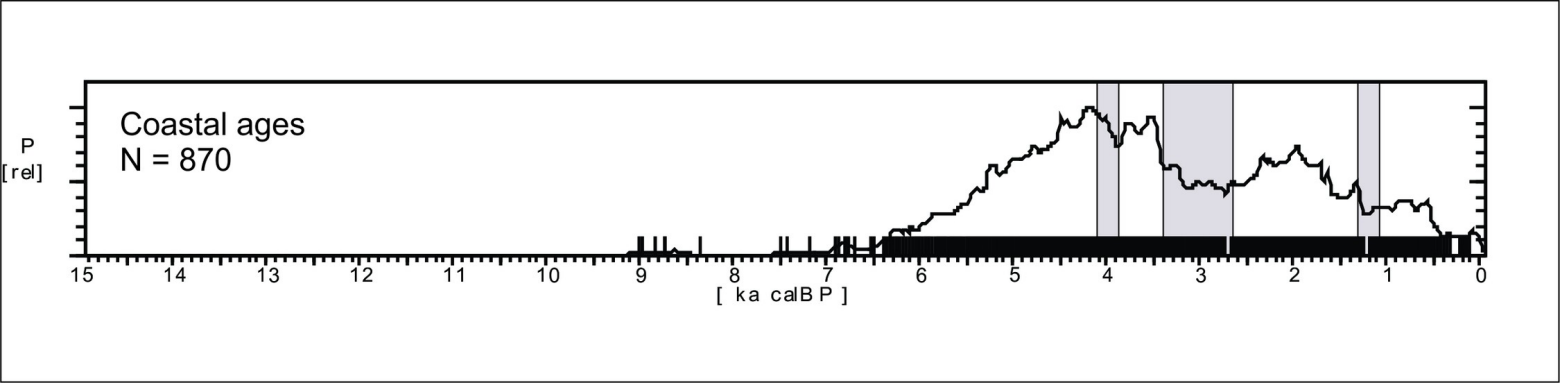
Summed probability distribution graphs for all coastal sites. Grey bars show the 1) a sharp depression in ages ca. 4 ka BP; 2) a pronounced valley around 3 ka BP; 3) another sharp decrease ca. 1.3 ka BP.

Events of climatic instability or shift between climatic conditions mentioned around 3.0 ka BP [205–208] can be related to the depressions in the number of ages in all curves shown at Fig 32, and is also apparent when we take the Brazilian coast (962 ages) as a whole (Fig 34). The 4.0 ka BP depression can be correlated to the drough detected by Lopes et al. [209], and the sharp oscillations in the proxies studied by Sallun et al. [185], and is well marked in the SC/RS and SP/PR curves (Fig 32B and 32C), but not in the ES/RJ (Fig 32A). It might also be related to the 4.2 ka RCC [6, 92]. However, we have to keep in mind the role of the MRSLs, that can be either independent of the climatic shifts abovementioned, or be in fact one of the results of these same climatic shifts, both factors affecting humans.

The behavior of the MRSL in the last 7000 years is contentious (see S6 File), with some authors finding evidence of a continuous descent after a peak at ca. 5.1 ka BP, while others still recognize possible events of sea levels below the present in the late Holocene [210, 211]. The close relationship between the location of shell middens and coast lines is well established in contexts that were intensively studied, either in Brazil or in other settings [212–216]. However, Angulo et al. ([217]:495) dismissed the archaeological evidence on the basis of “various possibilities that could have led an unknown culture to initiate a mound underwater”. We do not think that this claim is very parsimonious, and our data in fact gives some support to the idea that the lowering of sea level after the maximum transgression at 5.1 ka BP, if not following the exact scenario proposed by Martin and colleagues [218–220], may at least be a little more complex than the smooth lowering proposed by Angulo and colleagues [221, 222]. When we examine Fig 34, it is possible that at least the ca. 4.0 and 3.0 ka BP age depressions could be linked to coastal progradation, when human groups would follow sea level lowering episodes, and part of the archaeological sites created during these episodes would be destroyed by wave erosion or buried under coastal sediments during retrogradation, in the subsequent sea level rising periods. However, the ES / RJ coast did not show this depression around 4 ka (Fig 32A). On the contrary, there is a peak of ages. This suggests that RCCs could be invoked as the most parcimonious explanation (Table 6).

## Discussion

A feature somewhat common in recent attempts to match archaeological and paleoenvironmental data is the urge to combine “dry” periods with some kind of cultural disturbance [223] and, conversely, “wet” periods as “amelioration” [109]. We hope our data shows that climatic variability is a more parsimonious explanation in several cases where the decrease of the archaeological signal can be perceived, at least for three reasons: 1) it is unlikely that climate became “dry” for several millennia in Tropical South America; 2) if conditions of “dryness” were stable at least for some human generations, people would start to adapt to the new environments and after a relatively short period (in geological terms), the “dry” areas would be reoccupied; 3) several inconsistencies between paleoclimate proxies can be explained by this rationale, allowing for the contemporaneity of “wet” and “dry” signals in a given region.

When we look at the overall picture of age distributions in Eastern South America, it is possible to recognize some ubiquitous features. One of them is the decrease in ages ca. 11.5 ka BP that appears in almost all regions, with a notable exception in Western Nordeste. This feature is probably linked somehow to the Younger Dryas (YD) cold event, and if not to the event itself (which comprises the interval between 12.9 ka BP and 11.6 ka BP–Carlson 2010) at least to the interval called YD termination (Cheng et al., 2020), which matches the archaeological signal well. Our inferences are also supported by recent findings using Y chromosome sequences found in aDNA, which detected a major genomic impact on Amerindian populations related to the YD (Paz Sepúlveda et al., 2022). A second ubiquitous feature is marked by the 8.2 ka event, a RCC that was detected across the globe [6, 224] and whose impact on humans is well acknowledged elsewhere [225–227]. Several of the SPD curves we compiled show some weakening of the archaeological signal ca. 8.2 ka BP, in some cases with strong cultural implications (for instance, the abandonment of Lagoa Santa [228]. Again, Western Nordeste is antiphased, with a peak of ages around 8.2 ka BP (Fig 16A). Some RCCs seem absent altogether in vast areas. Such is the case of the 9.2 ka event, well marked in Central Brazil but not present in the Amazonian Lowlands, Nordeste, and Southern Brazil. The 4.2 ka event is present in the Amazonian Lowlands, Nordeste, and Central Brazil but extremely weak in Southern Brazil.

Regarding the several instances of antiphased age structure between areas, it is important to stress that we do not propose that a specific people left a specific area, for instance, Central Amazonia, and went to another area, such as Eastern Amazonia. There are still huge portions of Eastern South America without a good coverage in terms of ages, and the determination of populational shifts has to be made hand in hand with good culture historical data (i.e., reliable artifact analysis and chronology). However, the fact that some areas show apparent depopulation where others do not, suggests at least patterns that need to be addressed in future investigations, and should be taken into account in archaeological explanations, going beyond the “people’s choice” commonsensical perspective. The acknowledgment that large areas were depopulated during various periods throughout the Holocene helps to explain the large expanses of territory that have been occupied by distinct human groups since the Pleistocene transition in Eastern South America (see [199, 229] for an in-depth discussion). Such large territories as we see archaeologically are probably the outcome of strategies that prioritized territorial mapping and changing the focal area of occupation, rather than promoting new subsistence or intensification strategies. The Lagoa Santa archaeological record is a good example, since the focal area was initially occupied between 12.5 and 8.2 ka, abandoned for 3000 years, and reocuppied by the same cultural group between 5.3 and 4.0 ka [228]. We do not mean that no individual of the group ever visited the region for 3000 years; quite on the contrary, the territory was probably large enough to allow the maintenance of the traditional subsistence practices elsewhere (probably towards north) and the former region of focal occupation was still inside their territory, perhaps being visited sporadically during hunting or ceremonial activites. An ethnographic parallel can be made with the Bororo Indians, whose territory spanned from Bolivia (west), central/southern portion of Goiás (east), the Miranda river in Mato Grosso do Sul (south), and the sources if the Xingu river [230]. This territory comprised ca. 540,000 sq km (208,000 sq miles), the approximate size of France, and was entirely covered by boys and their adult companions during the rite of passage ceremony into adulthood.

Dating issues comprise an important aspect when comparing archaeological and paleoenvironmental data: most archaeological ages are obtained by means of wood charcoal, whereas paleoenvironmental studies commonly rely on soil organic matter (SOM). The inadequacy of SOM as a reliable chronological tool, even when the most stable fraction (humin) is used, was already demonstrated [231]. Ages obtained by SOM tend to be rejuvenated by several millennia when compared to charcoal, probably due to vertical migration. In some instances, not even charcoal is free of problems, especially where the water table is present and water is circulating, such as the case of alluvial plans and the deepest portions of soil profiles subject to annual water table oscillations. In these cases, charcoal contamination by younger organic matter brought by circulating water probably explains the common occurrence of age inversions in the deepest portions of soil profiles [98]:2311; [163]:33; [231]:21; [232]:211; [233]:96; [234]:78.

Charcoal influx in sediments poses an important problem to be dealt in the future. For instance, in Santa Catarina Jeske-Pieruschka et al. [235] pointed to the presence of relatively high concentrations of charcoal, as well an anomalous positive correlation between charcoal influx and forest species frequency (r = 0.57) in the late Pleistocene, when the opposite occurs during the Holocene (r = -0.59). These and similar observations by other authors [236–239] are intriguing and can be related to fires induced by human action, since high rates of natural fire are not expected in cold and wet environments.

Another important issue that must be taken into account in future paleoenvironmental reconstructions is the role of humans as plant dispersers. As an example, Buso Jr. et al. [240] detected pollen of some Amazonian rainforest species along the SE Brazilian coast during the mid-Holocene, suggesting a “connection between Amazonia and Atlantic Forest” ([240]:1759), which would mean very humid conditions in order to overcome the present biogeographic barrier which is represented today by *Cerrado* and *Caatinga* biomes. The listed genera includes *Glycydendron* (edible fruit), *Rinorea* (edible fruit, medicinal use; Correia 2017), *Senefeldera* (a fast growing pioneer tree, possessing pharmacological potential; Vieira et al., 2018), *Symphonia* (medicinal use, latex used as glue to fix canoes, arrow points, body painting [241, 242]; *Borismene* (a vine from the *Minispermaceae* Family,with medicinal use, also possess a compound widely used as “curare” poison [243]; *Macoubea* (also used as “curare” poison to fish or hunt [244]). De Oliveira et al. [115] also found pollen of Amazonian species in NE Brazil ca. 12.5 ka BP, and they all can be considered of human interest, according to Rios and Pastore Jr. [242]: *Cecropia* (edible fruits, fibres used for ropes and string-making, several medicinal uses), *Cedrela* (anti-malarian properties), *Pouteria* (anti-diarrheal, anti-thermal), *Protium* (resin with medicinal properties), *Simarouba* (widely known for its medicinal properties; Lorenzi and Matos 2002), *Symphonia* (mentioned above) and *Trichilia* (purgative). Naturally the success in the reproduction of these plants depends on local climatic conditions, but their spread rates and capacity to overcome biogeographic barriers can be related to human action, as already stated by other authors [245–248].

Sea level changes pose enormous challenges in the coastal region, one of them being the destruction / submersion of archaeological sites, but evidence and means to detect submerged shell middens are increasing (e.g., Hale et al., 2021). It is worth noting that Toldo Jr. et al. [249] performed a high-frequency seismic survey at Patos lagoon (RS) and detected two possible shell middens over a (possibly) Pleistocene age surface, buried under 6 m of mud. The estimated age for the onset of mud deposition is 8 ka BP, which would represent a *terminus ante quem* age for the buried surface. Even if the seismic anomalies are not shell middens, this gives a good example of the difficulties of finding such sites, but also shows the possibility that many of them could be very well preserved in specific geomorphic conditions (in this case, behind a sand barrier when the sea level rose and formed the lagoon).

## Conclusions

Our original observations made in 2005 are still valid; there were indeed major areas of Eastern South America which showed a diminution of the archaeological signal. However, the new data shows that there is a good match between extreme climatic variability and instances of decrease or gaps in the number of archaeological ages, which we consider as an *archaeological signal*, a surrogate for the intensity of human presence in a given area. Our approach was directed towards the detection of rapid climatic changes (RCCs), using the coefficient of variation (CV) of the values of paleoenvironmental proxies published by other authors, rather than focusing on “dry” or “wet” periods. Our data strongly suggests that RCCs are the most parsimonious explanations to account for the archaeological age patterns observed throughout Eastern South America. In several instances, we observed signals of the most conspicuous, globally detected RCCs (such as YD and the 8.2 ka event) as well as other events that, in spite of being more localized, can be of interest in modelling future climatic scenarios with the steady global mean temperature rising.

The fact that not all RCCs are globally detectable or can be perceived in the exact same time interval [250] should be no surprise, for the reasons we advanced throughout this article: different proxies, different dating methods, different resolutions, not to mention the vagaries of atmospheric circulation patterns. Some RCCs are very marked and widrespread, such as the 8.2 ka event, but an important aspect to bear in mind is that even RCCs that are not strongly visible in the paleoenvironmental record are sometimes very well marked in the archaeological record across different regions of the globe. A good example is the 4.2 ka event, which imposed a strong sinal on the archaeological record in different regions of the world, such as the Mediterranean [251], Northwestern Europe [252], China [253], Japan [254], India [255], Mesopotamia [16], Egypt [91], North America [256, 257], and South America [109]. At the same time, the 4.2 ka event is considered by some authors as not sufficiently marked from the point of view of other paleoenvironmental proxies [250, 258]. This highlights the fact that the RCCs were originally defined as human-sensitive, and occurr on a human time scale [6].

Finally, as put by Sandweiss et al. [259]:8276, “archaeological sites (…) record conditions from every ecosystem and time period important to human history and provide proxies where more traditional ones are absent, rare, or compromised”. We would add that archaeological data should be taken into account more seriously in paleoclimate reconstructions, especially regarding charcoal input and vegetal species occurrence / spread. Moreover, archaeological data should be understood as paleoclimate proxies *de facto*, since humans always were, are, and will be extremely sensitive to climatic conditions, and therefore can be regarded as good paleoenvironmental markers [22]. This is even more true for complex, hierarchical social systems whose subsistence is based on a extremely restricted assemblage of plant and animal domesticated species, and with strong territorial constraints, such as the case of our own industrial society.

## Supporting information

S1 File Theoretical underpinnings. (DOCX)

S2 File Methods. (DOCX)

S3 File Paleoenvironments in the Forested Amazonian Lowlands. (DOCX)

S4 File Paleoenvironments in Central Brazil. (DOCX)

S5 File Paleoenvironments in Southern Brazil. (DOCX)

S6 File Paleoenvironments along the Atlantic Coast. (DOCX)
