# Supplementary material for: Human-environment interaction during the Holocene in Eastern South America: Rapid climate changes and population dynamics
Source: PLoS One. 2025 Feb 3;20(2):e0315747. doi: 10.1371/journal.pone.0315747 (PMC11790176; doi:10.1371/journal.pone.0315747)
Supplement: S1 File — (DOCX) [file pone.0315747.s001.docx]

SUPPORTING INFORMATION 1

Theoretical underpinnings

Several problems can interfere with the often proposed relationship between number of ages and population, and examples of criticisms of the use of this approach are abundant. Such criticisms range from healthy caution [1] to the idea that the method is useless [2]. The main issues raised are related to sample size, sample representativeness, if the universe being sampled obbeys a normal distribution, the effect of the calibration curves, the effect of the software used in calculating the SPDs, taphonomic factors, research interest biases, geographic biases, money biases, and so on [3]. Many if not all of these factors are simply beyond the control of any reasearcher, what can lead to a more “nihilistic” view to the use of SPDs. Here we consider the shapes of SPDs as a valuable information about the presence or absence of people, and not so much about the *number* of people. This is because a single site with several ages can produce a “peak” in the SPD curve, but this peak is not necessarily related to the number of people living in a given area. So, we ask the reader to pay attention to the troughs, or intervals with few ages, and not so much to the peaks.

Be that as it may, taking these possible biases into account is not only necessary, but mandatory in a proper scientific posture. However, in our opinion, the best way to deal with these problems is to look at SPDs as proxies [4], as lines of evidence that need to be contrasted, verified or compared with other independent lines of evidence in order to provide a possible explanation for the observed phenomena. For example, while taphonomy can be invoked as a bias that would lead to the destruction of older sites and overrepresentation of younger ones [5], we have the exact opposite in some areas of Central Brazil, where the ages of the Early Holocene are much more abundant than those of the Middle Holocene [6,7]. While some authors claim that historically documented population crashes can barely be detected by SPDs [8], we see all SPD curves we run in this paper showing major troughs after ca. 1500 AD, when the Europeans arrived. In spite of the fact that there can be a bias in these post-colonial troughs related to research interests or taphonomic processes, if this was always the case, the troughs would likely be consistent and simultaneous. However, in areas where the impact of the European presence is historically documented as early and severe (e.g., SE Brazil), the troughs are visible right after AD 1500, whereas in more remote areas (e.g., Amazon, Nordeste, Mato Grosso), the diminution in the number of ages occurs much later. With these examples we want to illustrate that the possibility of biases does not imply any certainty that they will happen in every case study. They have to be checked against other lines of evidence. The same caution and cross-checking goes for any other proxy used in archaeology or Earth Sciences in general.

Regarding paleoenvironments, it is important to acknowledge that each proxy has its own potential and liabilities, and these characteristics have to be taken into consideration in order to interpret the data [9,10]. High resolution proxies such as ice cores and speleothems, which can provide data on decadal or even annual intervals, are spatially restrict. Moreover, one of the main proxies used in speleothem studies, delta ^18^O, is prone to errors related to salinity and temperature corrections that still need to be adequately accounted for (see [11]:40; [12]:409). On the other hand pollen, phytolith, and soil isotopic carbon analysis, which can be performed on a variety of environments such as open-air sites, peat bogs, lakes, rock shelters, and river terraces, can be considered low resolution proxies, with a strong time averaging and, therefore, subject of missing short time environmental changes. Another important issue is related to the geographical positioning of the paleoenvironmental data [13]; sites located in the central area of a given biome can be more complacent to climate change than sites near the border, or in ecotones [14,15]. Last but not least, the geometry and hydrology of lakes (depth/surface ratio, water input/output balance) can account for sharp differences on sedimentation rates, organic matter and pollen preservation, and geochemical characteristics of the sediments [16]. In terms of chronology, we do not expect a perfect match between paleoenvironmental studies and archaeological age patterns for, at least, five reasons: 1) because most paleoenvironmental studies are related to sediment cores extracted from lake bottoms, and in these cases some events of climatic change have their ages interpolated; 2) strong reservoir effects can be present when dating the organic fraction in lakes (with mismatches between 2000 and 4700 years; [17]); 3) there is a known difference in ages obtained by organic matter in sediments and single pieces of charcoal commonly dated in archaeological sites, the former being a mixture of several depositional events, the latter representing the death of a single plant [18]. Moreover, different organic fractions (e.g., humates, humin) are know to produce different ages [19]; 4) there is growing evidence that even humin, the most stable organic fraction, is liable to downward movement, at least in sandy soils [20]; 5) in the case of U-Th dating commonly used in speleothems, we can also have a difficult match between the very high “grain” of chronological determination reaching annual resolution and the radiocarbon or luminescence archaeological ages with their comparatively large associated errors.

References

1. Bamforth DB, Grund B. Radiocarbon calibration curves, summed probability distributions, and early Paleoindian population trends in North America. J Archaeol Sci. 2012;39(6):1768–74. http://dx.doi.org/10.1016/j.jas.2012.01.017
2. Torfing T. Neolithic population and summed probability distribution of 14C-dates. J Archaeol Sci. 2015;63:193–8. <http://dx.doi.org/10.1016/j.jas.2015.06.004>
3. Crema ER. Statistical inference of prehistoric demography from frequency distributions of radiocarbon dates: A review and a guide for the perplexed. J Archaeol Method Theory. 2022;29(4):1387–418. http://dx.doi.org/10.1007/s10816-022-09559-5
4. Timpson A, Manning K, Shennan S. Inferential mistakes in population proxies: A response to Torfing’s “Neolithic population and summed probability distribution of 14C-dates.” J Archaeol Sci. 2015;63:199–202. http://dx.doi.org/10.1016/j.jas.2015.08.018
5. Surovell TA, Brantingham PJ. A note on the use of temporal frequency distributions in studies of prehistoric demography. J Archaeol Sci. 2007;34(11):1868–77. <http://dx.doi.org/10.1016/j.jas.2007.01.003>
6. Araujo AGM, Neves WA, Piló LB, Atui JPV. Holocene dryness and human occupation in Brazil during the “archaic gap.” Quat Res. 2005;64(3):298–307. <http://dx.doi.org/10.1016/j.yqres.2005.08.002>
7. Araujo AGM, Pugliese FA Jr, Santos R, Okumura M. Extreme cultural persistence in eastern-central Brazil: the case of Lagoa Santa Paleaeoindians. An Acad Bras Cienc. 2018;90(2 suppl 1):2501–21. <https://doi.org/10.1590/0001-3765201720170109>
8. Contreras DA, Meadows J. Summed radiocarbon calibrations as a population proxy: a critical evaluation using a realistic simulation approach. J Archaeol Sci. 2014;52:591–608. http://dx.doi.org/10.1016/j.jas.2014.05.030
9. Araujo AGM. Paleoenvironments and Paleoindians in Eastern South America. In: Stanford DJ, Stenger A, editors. Pre-Clovis in the Americas: International science conference proceedings. 1st ed.Washington, DC: Smithsonian Institution; 2014. pp. 221-261.
10. Grosjean M, Cartajena I, Geyh MA, Núñez L. (2003). From proxy data to paleoclimate interpretation: the mid-Holocene paradox of the Atacama Desert, northern Chile. Palaeogeogr Palaeoclimatol Palaeoecol. 2003;194(1-3):247-258. <https://doi.org/10.1016/S0031-0182(03)00280-3>
11. Baker PA, Fritz SC. Nature and causes of Quaternary climate variation of tropical South America. Quat Sci Rev. 2015;124:31–47. http://dx.doi.org/10.1016/j.quascirev.2015.06.011
12. Bueno ML, Pennington RT, Dexter KG, Kamino LHY, Pontara V, Neves DM, et al. Effects of Quaternary climatic fluctuations on the distribution of Neotropical savanna tree species. Ecography (Cop). 2017;40(3):403–14. <http://dx.doi.org/10.1111/ecog.01860>
13. He Y, Theakstone WH, Zhonglin Z, Dian Z, Tandong Y, Tuo C, et al. Asynchronous Holocene climatic change across China. Quat Res. 2004;61(1):52–63. <http://dx.doi.org/10.1016/j.yqres.2003.08.004>
14. Mayle FE, Power MJ. Impact of a drier Early–Mid-Holocene climate upon Amazonian forests. Philos Trans R Soc Lond B Biol Sci. 2008;363(1498):1829–38. http://dx.doi.org/10.1098/rstb.2007.0019
15. Marchant R, Hooghiemstra H. Rapid environmental change in African and South American tropics around 4000 years before present: a review. Earth Sci Rev. 2004;66(3–4):217–60. http://dx.doi.org/10.1016/j.earscirev.2004.01.003
16. Bush MB, De Oliveira PE, Colinvaux PA, Miller MC, Moreno JE. Amazonian paleoecological histories: one hill, three watersheds. Palaeogeogr Palaeoclimatol Palaeoecol. 2004;214(4):359–93. http://dx.doi.org/10.1016/j.palaeo.2004.07.031
17. Geyh MA, Grosjean M, Núñez L, Schotterer U. Radiocarbon reservoir effect and the timing of the late-glacial/early Holocene humid phase in the Atacama desert (Northern Chile). Quat Res. 1999;52(2):143–53. <http://dx.doi.org/10.1006/qres.1999.2060>
18. Araujo AGM, Pilo LB, Neves WA, Atui JPV. Human occupation and paleoenvironments in South America: expanding the notion of an “Archaic Gap.” Rev Mus Arqueol Etnol. 2006;(15–16):3-35. https://www.revistas.usp.br/revmae/article/view/89707
19. Rowe HD, Guilderson TP, Dunbar RB, Southon JR, Seltzer GO, Mucciarone DA, et al. Late Quaternary lake-level changes constrained by radiocarbon and stable isotope studies on sediment cores from Lake Titicaca, South America. Glob Planet Change. 2003;38(3–4):273–90. http://dx.doi.org/10.1016/s0921-8181(03)00031-6
20. Araujo AGM, Feathers JK, Hartmann GA, Ladeira FSB, Valezio EV, Nascimento DL, et al. Revisiting Alice Boer: Site formation processes and dating issues of a supposedly pre‐Clovis site in Southeastern Brazil. Geoarchaeology. 2022;37(1):32–58. https://doi.org/10.1002/gea.21831
