# Supplementary material for: Human-environment interaction during the Holocene in Eastern South America: Rapid climate changes and population dynamics
Source: PLoS One. 2025 Feb 3;20(2):e0315747. doi: 10.1371/journal.pone.0315747 (PMC11790176; doi:10.1371/journal.pone.0315747)
Supplement: S2 File — (DOCX) [file pone.0315747.s002.docx]

SUPPORTING INFORMATION 2

Methods

In some portions of Eastern South America, OSL and TL ages were widely used and they amount to approximately 3.8 % of our database. Their insertion in the SPDs is not possible, and this problem will have to be tackled in a near future, not only because the use of luminescence dating is going to increase, but also because both methods should be used in tandem. Radiocarbon suffers from several problems and can not be considered as the only way to date sites, not to mention the bias imparted by the method regarding very old ages or very young ones. We also decided to be inclusive in our database, and by this we mean that we are making available all radiocarbon or luminescence ages that were considered *bona fide* by the researchers who published them, regardless of the fact that other researchers consider these ages inconsistent with their own models or beliefs. The same goes for papers that select ages based on the standard deviations. A large standard deviation means low precision, not necessarily low accuracy. We take for granted that judgements about the appropriateness of the ages can be made individually by the reader, since we provided the full references. We prefer to publish an age with a large associated error than to ignore it. Once again, since we are providing the tables as supplementary material, it is up to the reader to disregard specific ages and run his/her own analysis.

In terms of the geographic location of the sites/ages, we chose to provide UTM coordinates of the nearest municipality, instead of providing “exact” locations. This decision was made on three grounds: first, in the scale of analysis we are presenting, the location of the nearest municipality is more than sufficient to provide an adequate overview of the spatial distribution of the ages; second, the majority of the sites published before the advent and popularization of handheld GPS devices do not have an accurate location and therefore, to provide an “exact” location would be meaningless; third, when trying to plot sites using available databases, be they compilations of data or first publications of a given site, it is common to observe that the apparently “exact” geographic coordinates were plainly wrong, falling outside a given region or even the state. This is something that plagues large databases, generally compiled by several researchers and their students, so we argue that it is much easier to detect errors and convey the right location of a given site, at least approximately, if the municipality is taken into account. Hence, our database has a redundant location scheme: state, municipality, and UTM coordinates. If by some reason the UTM is wrong, the reader at least knows in which state and municipality it is located. The only exception to this procedure was made in the Amazon region (states of Amazonas, Pará, Maranhão, Rondonia). Municipalities in the region are fairly large, and we chose to plot the location of the site when it was considered to be too far away from the nearest urbanized area. It is important to note that problems of spatial location are not confined to the archaeological literature, but also occur in the paleoenvironmental literature, in spite of the much smaller datasets; sometimes it is necessary to crosscheck several references until a given paleoenvironmental study site can be safely plotted on the map.

We used the Mann-Whitney U-test in order to compare the age patterns among different regions. The U-test is non-parametrical, and therefore does not assume a normal distribution for the population being sampled, and was used to compare the lists of values of central ages (obtained by luminescence) or the calibrated ages (obtained by radiocarbon [1-4]. This was made in order to avoid the use of visual inspection as the only tool to judge if two regions are showing similar or dissimilar patterns. The results were very useful, since the method avoided considering present-day administrative regions as homogeneous, as can be perceived in several instances along the paper. Moreover, since we are comparing two number lists (and not the ages and associated errors), in this specific case it is possible to use the calibrated radiocarbon and the luminescence data in tandem. All statistics were run using BioEstat 5.0 [5].

References

1. Cersoy S, Zazzo A, Rofes J, Tresset A, Zirah S, Gauthier C, et al. Radiocarbon dating minute amounts of bone (3-60 mg) with ECHoMICADAS. Sci Rep. 2017;7(1):7141. <http://dx.doi.org/10.1038/s41598-017-07645-3>
2. Dyke AS, Savelle JM, Szpak P, Southon JR, Howse L, Desrosiers PM, et al. An assessment of marine reservoir corrections for radiocarbon dates on walrus from the Foxe Basin region of Arctic Canada. Radiocarbon. 2019;61(1):67–81. http://dx.doi.org/10.1017/rdc.2018.50
3. Kim J, Wright DK, Hwang J, Kim J, Oh Y. The old wood effect revisited: a comparison of radiocarbon dates of wood charcoal and short-lived taxa from Korea. Archaeol Anthropol Sci. 2019;11(7):3435–48. <http://dx.doi.org/10.1007/s12520-018-0766-8>
4. Nakamura Y, Waku D, Wakiyama Y, Watanabe Y, Koganebuchi K, Nagaoka T, et al. Collagen of ancient bones gives an indication of endogenous DNA preservation based on next-generation sequencing technology. Anth Sci. 2024; 240109.
5. Ayres M, Ayres Jr. M, Ayres DL, Santos A. Bioestat 5.0 – Aplicações estatísticas na área de Ciências Bio-Médicas. 2007. <https://www.mamiraua.org.br/downloads/programas/>
