# Supplementary material for: Human-environment interaction during the Holocene in Eastern South America: Rapid climate changes and population dynamics
Source: PLoS One. 2025 Feb 3;20(2):e0315747. doi: 10.1371/journal.pone.0315747 (PMC11790176; doi:10.1371/journal.pone.0315747)
Supplement: S3 File — (DOCX) [file pone.0315747.s003.docx]

SUPPORTING INFORMATION 3

Paleoenvironments in the Forested Amazonian Lowlands

The reasons for the lack of agreement between paleoenvironmental studies in Amazonia are probably related to the geographical settings of the different paleoenvironmental sites [1,2]. For instance, Bush et al. [3] found a continuous record of forest in the last 50 ka BP for Hill of Six Lakes region (Lake Pata), with forest taxa comprising around 80 to 90% of the pollen, while Absy et al. [4] found a strong decrease of forest taxa at 60 ka BP, 40 ka BP, and between 23 and 11 ka BP for Carajás. While Six Lakes is located in the middle of the Amazon (Fig 3, number 2), Carajás is located in an ecotone between the Amazonian forest and savannah (or “cerrado” – Fig 3, number 7), 2000 km SE of Hill of Six Lakes. Hence, this disparity is easily accounted for (but see [5] for a revision of the Six Lakes results). As stated by Mayle and Power ([6]:1832), the degree of forest fragmentation is expected to be enhanced in ecotonal areas, and the magnitude of impact of droughts is likely to be inversely correlated to the mean annual precipitation, and directly correlated with the length and severity of the dry periods.

Holocene records for the Amazonian Lowlands were also reported at Curuçá lake (Fig 3, number 13; [7]), Curuá river (Fig 3, number 9; [75]), Tapajós lake (Fig 3, number 17; [77]), Calado lake (Fig 3, number 4 ; [7]), Caracaranã lake (Fig 3, number 1; [8]), Comprido lake (Fig 3, number 8; [9]), Tapera lake (Fig 3, number 18 ; [10]), Geral lake, Saracuri lake, and Santa Maria lake (Fig 3, numbers 10, 15, 16; [11]), Humaitá (Fig 3, number 5; [12]) and Vilhena – Ariquemes (Fig 3, number 41; [13]). Recent reviews of the available data [8,14,15] show that there is an ubiquitous pattern in all Amazonian lakes: low lake stands in the Mid-Holocene (ca. 7 to 6 ka BP), suggesting an overall decrease in the rainfall patterns which may be enventually related to the 6.4 ka RCC event (Wanner et al. 2015). Disturbances in the floristic pattern during the Mid-Holocene were perceived even in the “core” of the wettest portion of the Amazonian rainforest. Bush et al. ([11]:210) observed that in a 50,000 yr paleoecological record from northwestern Amazonia, only one sedimentary layer, at 5.6 ka BP, contained charcoal. According to Mayle and Power ([14]:1833), the Lake Pata record shows a pollen assemblage located between 6 and 7 ka BP “suggestive of a change from a closed-canopy forest to a forest/woodland (...) consistent with a response to reduced precipitation”. A somewhat contrasting line of evidence comes from speleothems from Paraíso cave, 200 km SW of Santarém. According to Wang et al. [16], the records suggest an increase in precipitation (142% in relation to modern levels) during the mid-Holocene, ca. 6 ka BP. To Smith and Mayle ([17]:13), the explanation of this pattern is still unclear.

An important consideration to be made and recognized by several authors [11,14,18] is the possible impact that human activities can have on the palynological record. For instance, taxa such as *Cecropia*, which are indicative of forest clearing and growth of pioneer species, can be related either to precipitation, natural fires or human activity, and sometimes a conjunction of all three factors.

References

1. Marchant R, Hooghiemstra H. Rapid environmental change in African and South American tropics around 4000 years before present: a review. Earth Sci Rev. 2004;66(3–4):217–60. http://dx.doi.org/10.1016/j.earscirev.2004.01.003
2. Hooghiemstra H, van der Hammen T. Neogene and Quaternary development of the neotropical rain forest: the forest refugia hypothesis, and a literature overview. Earth Sci Rev. 1998;44(3–4):147–83. <http://dx.doi.org/10.1016/s0012-8252(98)00027-0>
3. Bush MB, De Oliveira PE, Colinvaux PA, Miller MC, Moreno JE. Amazonian paleoecological histories: one hill, three watersheds. Palaeogeogr Palaeoclimatol Palaeoecol. 2004;214(4):359–93. <http://dx.doi.org/10.1016/j.palaeo.2004.07.031>
4. Absy ML, Cleef A, Fournier M, Martin L, Servant M, Siffedine A, Ferreira da Silva M, Soubies F, Suguio K., Turcq B, van der Hammen T. Mise en évidence de quatre phases d'ouverture de la forêt dense dans le sud-est de l'Amazonie au cours des 60,000 dernières années. Première comparaison avec d'autres régions tropicales. Comptes Rendus d'Academie des Sciences 1991 Serie II; 312:673-8.
5. D’Apolito C, Absy ML, Latrubesse EM. The Hill of Six Lakes revisited: new data and re-evaluation of a key Pleistocene Amazon site. Quat Sci Rev. 2013;76:140–55. <http://dx.doi.org/10.1016/j.quascirev.2013.07.013>
6. Mayle FE, Power MJ. Impact of a drier Early–Mid-Holocene climate upon Amazonian forests. Philos Trans R Soc Lond B Biol Sci. 2008;363(1498):1829–38. http://dx.doi.org/10.1098/rstb.2007.0019
7. Behling H. Late Quaternary environmental changes in the Lagoa da Curuça region (eastern Amazonia, Brazil) and evidence of Podocarpus in the Amazon lowland. Veg Hist Archaeobot. 2001;10(3):175–83. http://dx.doi.org/10.1007/pl00006929
8. Cordeiro RC, Turcq PFM, Turcq BJ, Moreira LS, Rodrigues RC, da Costa RL, et al. Acumulação de carbono em lagos amazônicos como indicador de eventos paleoclimáticos e antrópicos. Oecol Australis . 2008;12(01):130–54. <http://dx.doi.org/10.4257/oeco.2008.1201.12>
9. Moreira LS, Moreira-Turcq P, Cordeiro RC, Turcq B, Caquineau S, Viana JCC, et al. Holocene paleoenvironmental reconstruction in the Eastern Amazonian Basin: Comprido Lake. J South Am Earth Sci. 2013;44:55–62. http://dx.doi.org/10.1016/j.jsames.2012.12.012
10. Toledo MB de, Bush MB. Vegetation and hydrology changes in Eastern Amazonia inferred from a pollen record. An Acad Bras Cienc. 2008;80(1):191–203. http://dx.doi.org/10.1590/s0001-37652008000100014
11. Bush MB, Silman MR, de Toledo MB, Listopad C, Gosling WD, Williams C, et al. Holocene fire and occupation in Amazonia: records from two lake districts. Philos Trans R Soc Lond B Biol Sci. 2007;362(1478):209–18. http://dx.doi.org/10.1098/rstb.2006.1980
12. Cohen MCL, Rossetti DF, Pessenda LCR, Friaes YS, Oliveira PE. Late Pleistocene glacial forest of Humaitá—Western Amazonia. Palaeogeogr Palaeoclimatol Palaeoecol. 2014;415:37–47. <http://dx.doi.org/10.1016/j.palaeo.2013.12.025>
13. Pessenda LCR, Gomes BM, Aravena R, Ribeiro AS, Boulet R, Gouveia SEM. The carbon isotope record in soils along a forest-cerrado ecosystem transect: implications for vegetation changes in the Rondonia state, southwestern Brazilian Amazon region. Holocene. 1998;8(5):599–603. http://dx.doi.org/10.1191/095968398673187182
14. Mayle FE, Power MJ. Impact of a drier Early–Mid-Holocene climate upon Amazonian forests. Philos Trans R Soc Lond B Biol Sci. 2008;363(1498):1829–38. http://dx.doi.org/10.1098/rstb.2007.0019
15. Prado LF, Wainer I, Chiessi CM, Ledru M-P, Turcq B. A mid-Holocene climate reconstruction for eastern South America. Clim Past. 2013;9(5):2117–33. http://dx.doi.org/10.5194/cp-9-2117-2013
16. Wang X, Edwards RL, Auler AS, Cheng H, Kong X, Wang Y, et al. Hydroclimate changes across the Amazon lowlands over the past 45,000 years. Nature. 2017;541(7636):204–7. <http://dx.doi.org/10.1038/nature20787>
17. Smith RJ, Mayle FE. Impact of mid- to late Holocene precipitation changes on vegetation across lowland tropical South America: a paleo-data synthesis. Quat Res. 2018;89(1):134–55. http://dx.doi.org/10.1017/qua.2017.89
18. Toledo MB. Holocene Vegetation and Climate History of Savanna-forest Ecotones in Northeastern Amazonia. PhD dissertation, Florida Institute of Technology. 2004.
