# Supplementary material for: Human-environment interaction during the Holocene in Eastern South America: Rapid climate changes and population dynamics
Source: PLoS One. 2025 Feb 3;20(2):e0315747. doi: 10.1371/journal.pone.0315747 (PMC11790176; doi:10.1371/journal.pone.0315747)
Supplement: S4 File — (DOCX) [file pone.0315747.s004.docx]

SUPPORTING INFORMATION 4

Paleoenvironments in Central Brazil

Based on palynological analyses, some authors proposed that a very dry Last Glacial Maximum (LGM) and Late Glacial (since 19 ka BP) was followed by an increase in moisture beginning ca. 6.0 – 5.0 ka BP, leading to modern conditions [1-4], while other authors proposed a different scenario, with a dry and cooler LGM followed by a moister and still cool Late Glacial between ca. ca.10 and 8.5 ka BP, and then a very dry period between 8.5 and 4 ka BP, followed by the onset of modern conditions after it [5-9]. These studies were carried out at Águas Emendadas swamp (Fig 3, number 107; [1]), Cromínia swamp (Fig 3, number 45; [2]), Salitre (Fig 3, number 60; [6]), Lagoa do Pires (Fig 3, number 67; [5]). It is also important to note that, for unknown reasons, the majority of the paleoenvironmental studies for Central Brazil are concentrated towards East, mainly in Minas Gerais State. Other portions of this vast area are inside a “scientific and cultural shadow” to use an expression by McGlue et al. [10] addressing the lack of data in the Pantanal, the world’s largest wetland system (135,000 km^2^ [11]).

References

1. Barberi M, Salgado-Labouriau ML, Suguio K. Paleovegetation and paleoclimate of “Vereda de Águas Emendadas”, central Brazil. J South Am Earth Sci. 2000;13(3):241–54. <http://dx.doi.org/10.1016/s0895-9811(00)00022-5>
2. Ferraz-Vicentini KR, Salgado-Labouriau ML. Palynological analysis of a palm swamp in Central Brazil. J South Am Earth Sci. 1996;9(3–4):207–19. <http://dx.doi.org/10.1016/0895-9811(96)00007-7>
3. Salgado-Labouriau ML, Casseti V, Ferraz-Vicentini KR, Martin L, Soubiès F, Suguio K, et al. Late Quaternary vegetational and climatic changes in cerrado and palm swamp from Central Brazil. Palaeogeogr Palaeoclimatol Palaeoecol. 1997;128(1–4):215–26. http://dx.doi.org/10.1016/s0031-0182(96)00018-1
4. Salgado-Labouriau ML, Barberi M, Ferraz-Vicentini KR, Parizzi MG. A dry climatic event during the late Quaternary of tropical Brazil. Rev Palaeobot Palynol. 1998;99(2):115–29. http://dx.doi.org/10.1016/s0034-6667(97)00045-6
5. Behling H. South and southeast Brazilian grasslands during Late Quaternary times: a synthesis. Palaeogeogr Palaeoclimatol Palaeoecol. 2002;177(1–2):19–27. http://dx.doi.org/10.1016/s0031-0182(01)00349-2
6. Ledru M-P. Late Quaternary environmental and climatic changes in central Brazil. Quat Res. 1993;39(1):90–8. http://dx.doi.org/10.1006/qres.1993.1011
7. Ledru M-P, Braga PIS, Soubiès F, Fournier M, Martin L, Suguio K, et al. The last 50,000 years in the Neotropics (Southern Brazil): evolution of vegetation and climate. Palaeogeogr Palaeoclimatol Palaeoecol. 1996;123(1–4):239–57. http://dx.doi.org/10.1016/0031-0182(96)00105-8
8. Martin L, Bertaux J, Corrège T, Ledru M-P, Mourguiart P, Sifeddine A, et al. Astronomical Forcing of Contrasting Rainfall Changes in Tropical South America between 12,400 and 8800 cal yr B.P. Quat Res. 1997;47(1):117–22. http://dx.doi.org/10.1006/qres.1996.1866
9. Servant M, Maley J, Turcq B, Absy M-L, Brenac P, Fournier M, et al. Tropical forest changes during the late quaternary in African and South American lowlands. Glob Planet Change. 1993;7(1–3):25–40. http://dx.doi.org/10.1016/0921-8181(93)90038-p
10. Mcglue MM, Silva A, Assine ML, Stevaux JC, Pupim F. Paleolimnology in the Pantanal: Using Lake Sediments to Track Quaternary Environmental Change in the World’s Largest Tropical Wetland. In: Bergier  M, Assine IL, editors. Dynamics of the Pantanal Wetland in South America. Springer; 2016. pp. 51–81.
11. Whitney BS, Mayle FE, Punyasena SW, Fitzpatrick KA, Burn MJ, Guillen R, et al. A 45kyr palaeoclimate record from the lowland interior of tropical South America. Palaeogeogr Palaeoclimatol Palaeoecol. 2011;307(1–4):177–92. http://dx.doi.org/10.1016/j.palaeo.2011.05.012
