# Supplementary material for: Human-environment interaction during the Holocene in Eastern South America: Rapid climate changes and population dynamics
Source: PLoS One. 2025 Feb 3;20(2):e0315747. doi: 10.1371/journal.pone.0315747 (PMC11790176; doi:10.1371/journal.pone.0315747)
Supplement: S5 File — (DOCX) [file pone.0315747.s005.docx]

SUPPORTING INFORMATION 5

Paleoenvironments in Southern Brazil

At Serra dos Órgãos, a subrange of Serra do Mar, 2130 m a.s.l. ( Fig 3, number 92; [1]), conditions in the early Holocene suggest the presence of “campos” (grassy savannah) since 12.4 ka BP. Between 12.3 and 11.8 ka BP, *campos* vegetation decreases and arboreal taxa increase, suggesting warm and wet conditions. The trend is reverted between 11.8 and 10.8 ka BP, with another expansion of *campos* and regression of forest. Between 10.8 and 4.9 ka BP there is a relatively steady balance between “campos” and forest taxa, with a slow upward migration of forest taxa and the local extinction of *Araucaria*. Climatic conditions remained practically the same since 4.9 ka BP up to present times, with a slight increase in moisture around 880 cal BP.

At Serra da Bocaina (Fig 3, number 84; [2]) pollen data from two cores, one at 1500 m a.s.l. and the other 1650 m a.s.l. show a lesser frequency of herbs in the Early Holocene (10.8 to 7.3 ka BP) when compared to the Late Glacial. However, the presence of herbs is still predominant. The interpretation is of a relatively warm but still dry climate, with the absence of *Araucaria* trees. Between 7.3 and 4.6 ka BP there is a marked forest expansion, suggesting a moister climate, and from 4.6 and 1.3 ka BP a slight decrease in forest taxa, a scenario that is very similar to Morro de Itapeva (Fig 3, number 89; [3]). For the Campos do Jordão region, (Fig 3, number 85; [4]) it was found evidence of soil formation (climatic stability) at 11.5 ka BP and since 8.3 ka BP.

Seixas et al. [5] presented a phytolith and carbon isotope study at Paraíba do Sul valley (Fig 3, number 93). The soil profile showed two stone lines, which we interpret as evidence of rhesistatic conditions. The lower stone line, at 150-158 cm depth, was dated at 8.9 ka BP. The upper stone line, at 125-138 cm depth was dated at 8.4 ka BP [6], both ages obtained by ^14^C AMS on soil organic matter (SOM, which imparts some uncertainties as we will discuss below). The authors found a trend of strong variability in the D/P phytolith index (which measures the density of tree cover) and also in the Bi index (hydric stress) in the lowermost portion of the profile (before 8.2 ka BP).

At Colônia, (Fig 3, number 86; [7]) it was found an increase in moisture since the beginning of the Holocene, with a full development of rainforest after 9 ka BP, but between 6.2 and 3.7 ka BP there was a major reduction in tree cover, followed by another expansion until reaching present-day fully developed rainforest.

In the Tamanduá river, Turcq et al. [8,9] (Fig 3, number 87) found evidence of a major erosion episode somewhere between 11.5 and 6.9 ka BP, followed by deposition suggesting climatic stability until the present.

Storani and Perez-Filho [10] detected a terrace formation at Mogi-Guaçu river (Fig 3, number 108) dated at 1.9 ka BP, considered by the authors as evidence of a dry climatic pulse.

Cruz [11] presented speleothem data from São Paulo (Santana cave - Fig 3, number 88) and Paraná (Botuverá cave - Fig 3, number 97) suggesting the expansion of rain forest and stability of the precipitation regime from 9.0 – 8.0 ka BP onwards. At Serra dos Campos Gerais, Paraná hinterland, (Fig 3, number 95; [12]), the Holocene is marked by an increase in temperature and moisture. Even so, the area was still occupied mainly by grassy savannah from 11.0 to 3.0 ka BP. Forest taxa increased when compared to the Late Glacial, suggesting an expansion of the forest patches in the valley bottoms. Between 3.0 and 1.4 ka BP there is an increase in *Araucaria*, that had been probably confined to the valleys, and which start to occupy the highest portions of the landscape.

At Serra de Araçatuba (Fig 3, number 94; [13]), the data suggest the permanence of cold and dry conditions (prevalence of herbaceous vegetation, virtually no trees) during almost the whole Holocene, from 12.9 to 2.0 ka BP, probably due to the height of 1500 m a.s.l.. From 2.0 ka BP onwards, there is an increase in the proportion of trees, suggesting wetter conditions. Towards the coast, only 40 km east of Serra de Araçatuba but 1500 m lower, data from Volta Velha (Fig 3, number 98; [14]) suggests that rainforest started to develop only in the beginning of the Holocene, probably expanding from the north in a successional way. By 7.7 ka BP rainforest is fully established.

In the hinterlands of Rio Grande do Sul, at São Francisco de Assis ( Fig 3, number 100; [15]), vegetation cover was composed mainly of grassy savannah (“campos”) since the LGM, even when climate changed from cold and dry to warm and dry in the Pleistocene / Holocene transition. An increase in gallery forest started only about 5.2 ka BP, signalling wetter conditions. By 1.6 ka BP the gallery forest was even more developed. The increase in humidity during the mid-Holocene and the full establishment of forests around 1.0 ka BP was perceived in other locations of Rio Grande do Sul, such as Cambará do Sul (Fig 3, number 101; [16]) and São Francisco de Paula (Fig 3, number 102; [17]). In this last locality, Leonhardt and Lorscheitter [18] found signals of cold and dry climate between 25 ka BP and 12.5 ka BP, with a peak of aridity ca. 16 ka. From 12.5 ka onwards the climate became moister, with dry and warm conditions appearing from 9.7 ka BP to 6.5 ka BP. A gradual increase in humidity was observed between 6.5 ka BP and 4 ka BP. Between 4 ka BP and 2 ka BP *Araucaria*  expanded, suggesting moister conditions.

The Mina Modelo pond (Fig 3, number 109), studied by Gadens-Marcon et al. [19] using pollen and geochemical analyses, showed evidence of climatic instability between 6.8 and 6.0 Ka BP. According to the authors,“the rainfall remained relatively constant until about 7000 years ago. Thereafter, rainfall patterns become irregular. (...).The oscillations between high and low rainfall periods become progressively more frequent and the high moisture levels of early Holocene tend to decrease” ([19]:111).

References

1. Behling H, Safford HD. Late‐glacial and Holocene vegetation, climate and fire dynamics in the Serra dos Órgãos, Rio de Janeiro State, southeastern Brazil. Glob Chang Biol. 2010;16(6):1661–71. <http://dx.doi.org/10.1111/j.1365-2486.2009.02029.x>
2. Behling H, Dupont L, DeForest Safford H, Wefer G. Late Quaternary vegetation and climate dynamics in the Serra da Bocaina, southeastern Brazil. Quat Int. 2007;161(1):22–31. <http://dx.doi.org/10.1016/j.quaint.2006.10.021>
3. Behling H. Late Quaternary vegetation, climate and fire history from the tropical mountain region of Morro de Itapeva, SE Brazil. Palaeogeogr Palaeoclimatol Palaeoecol. 1997b;129(3–4):407–22. http://dx.doi.org/10.1016/s0031-0182(97)88177-1
4. Modenesi-Gauttieri MC. Hillslope deposits and the Quaternary evolution of the altos campos - Serra da Mantiqueira , from Campos do Jordão to the Itatiaia massif. Revista Brasileira de Geociências. 2000;30:508–14.
5. Seixas AP, Coe HG, Silva AC, Lepsch I, Parolin M, Macario K. Reconstituição das condições paleoambientais relacionadas à ocorrência de linhas de pedra em Latossolo no médio vale do Rio Paraíba do Sul (RJ). Revista da Associação Nacional de Pós-graduação e Pesquisa em Geografia (Anpege). 2019;15:29–53.
6. Seixas AP. Condições paleoambientais associadas à ocorrência de linhas de pedra em latossolo no Médio Vale do Rio Paraíba do Sul-RJ. Master´s thesis, Fluminense Federal University; 2017
7. Ledru M-P, Mourguiart P, Riccomini C. Related changes in biodiversity, insolation and climate in the Atlantic rainforest since the last interglacial. Palaeogeogr Palaeoclimatol Palaeoecol. 2009;271(1–2):140–52. http://dx.doi.org/10.1016/j.palaeo.2008.10.008
8. Turcq B, Albuquerque ALS, Cordeiro RC, Sifeddine A, Simoes Filho FFL, Souza AG, et al. Accumulation of organic carbon in five Brazilian lakes during the Holocene. Sediment Geol. 2002;148(1–2):319–42. http://dx.doi.org/10.1016/s0037-0738(01)00224-x
9. Turcq B, Pressinotti MMN, Martin L. Paleohydrology and paleoclimate of the past 33,000 years at the Tamanduá River, central Brazil. Quat Res. 1997;47(3):284–94. http://dx.doi.org/10.1006/qres.1997.1880
10. Storani DL, Perez Filho A. Novas informações sobre geocronologia em níveis de baixo terraço fluvial do rio Mogi Guaçu, SP, Brasil. Rev Bras Geomorfol. 2015;16(2). http://dx.doi.org/10.20502/rbg.v16i2.656
11. Cruz FW. Estudo paleoclimático e paleoambiental a partir de registros geoquímicos quaternários em espeleotemas das regiões de Iporanga (SP) e Botuverá (SC). PhD Dissertation, University of São Paulo. 2003
12. Behling H. Late Quaternary vegetation, climate and fire history of the Araucaria forest and campos region from Serra Campos Gerais, Paraná State (South Brazil). Rev Palaeobot Palynol. 1997a;97(1–2):109–21. http://dx.doi.org/10.1016/s0034-6667(96)00065-6
13. Behling H. Late Quaternary vegetation, fire and climate dynamics of Serra do Araçatuba in the Atlantic coastal mountains of Paraná State, southern Brazil. Veg Hist Archaeobot. 2006;16(2–3):77–85. http://dx.doi.org/10.1007/s00334-006-0078-2
14. Behling H, Negrelle RRB. Tropical Rain Forest and climate dynamics of the Atlantic lowland, southern Brazil, during the late Quaternary. Quat Res. 2001;56(3):383–9. http://dx.doi.org/10.1006/qres.2001.2264
15. Behling H, Pillar VD, Bauermann SG. Late Quaternary grassland (Campos), gallery forest, fire and climate dynamics, studied by pollen, charcoal and multivariate analysis of the São Francisco de Assis core in western Rio Grande do Sul (southern Brazil). Rev Palaeobot Palynol. 2005;133(3–4):235–48
16. Behling H, Pillar VD, Orlóci L, Bauermann SG. Late Quaternary Araucaria forest, grassland (Campos), fire and climate dynamics, studied by high-resolution pollen, charcoal and multivariate analysis of the Cambará do Sul core in southern Brazil. Palaeogeogr Palaeoclimatol Palaeoecol. 2004;203(3–4):277–97. http://dx.doi.org/10.1016/s0031-0182(03)00687-4
17. Behling H, Bauermann SG, Neves PCP. Holocene environmental changes in the São Francisco de Paula region, southern Brazil. Journal of South American earth sciences. 2001;14(6):631-639. https://doi.org/10.1016/S0895-9811(01)00040-2
18. Leonhardt A, Lorscheitter ML. The last 25,000 years in the Eastern Plateau of Southern Brazil according to Alpes de São Francisco record. J South Am Earth Sci. 2010;29(2):454–63. http://dx.doi.org/10.1016/j.jsames.2009.09.003
19. Gadens-Marcon GT, Guerra-Sommer M, Mendonça-Filho JG, Mendonça J de O, Carvalho M de A, Hartmann LA. Holocene environmental climatic changes based on palynofacies and organic geochemical analyses from an inland pond at altitude in southern Brazil. Am J Clim Change. 2014;03(01):95–117. <http://dx.doi.org/10.4236/ajcc.2014.31009>
