# Supplementary material for: Human-environment interaction during the Holocene in Eastern South America: Rapid climate changes and population dynamics
Source: PLoS One. 2025 Feb 3;20(2):e0315747. doi: 10.1371/journal.pone.0315747 (PMC11790176; doi:10.1371/journal.pone.0315747)
Supplement: S6 File — (DOCX) [file pone.0315747.s006.docx]

SUPPORTING INFORMATION 6

Paleoenvironments along the Atlantic Coast

For the last 7000 years the behavior of the mean relative sea level (MRSL) along the Brazilian coast is subject to controversy; based on multiple proxies (geomorphic, sedimentological, biological, and archaeological), some authors [1-3] proposed at least two high magnitude oscillations after the maximum Holocene transgression which occurred ca. 5.1 ka BP, causing two events of MRSLs below the present sea level, meaning amplitudes up to 3 to 4 m, the first between 4.1 to 3.8 ka BP, and the second between 3.0 and 2.7 ka BP. Other authors disagreed [4-6], basing their claim on a single proxy (vermetids) and proposing a steady MRSL lowering after the 5.1 ka BP transgression, reaching the present sea level. The subject is still open, and there are strong and weak points in both arguments [7,8]. Be as it may, the impact of these events in terms of coastal geomorphology depend on local conditions, including tectonism, and global signals can be either enhanced or obscured. Significantly, the shape and declivity of the coastal shelf will be of paramount importance in terms of impact on humans; the same MRSL increase will impart a huge territorial loss in a wide shelf with low declivity, and a much less pronounced impact on a narrow shelf with high declivity. As we will see below, this geomorphic factor can potentially explain the overall features of the age curves, in tandem with changes in oceanic and atmospheric currents that can have impacts on biological productivity.

Regarding paleoenvironments along the Southern coast, several studies are available.

Gyllencreutz et al. [9] performed high-resolution sediment grain size analyses from three ocean cores across the Southern coast (Cores 7606, 7616, and 7620 - Fig 3, number 104, 105, 106) and found evidence of a weak ENSO (El Niño Southern Oscillation) activity from 7 to 5 ka BP, followed by an increase from 5 ka BP onwards. At ca. 3.0 – 2.0 ka BP the ENSO amplitude was further enhanced, causing increased “El Niño – La Niña” events. The authors also perceived different sensitivities between profiles, with the ENSO signal more visible in the Southern core (SC - 7606) and less pronounced in the northern core (RJ - 7620).

Buso Jr. et al. [10] provided data on pollen and phytoliths from Sooretama nature reserve, northern Espirito Santo coast (Fig 3, number 70) and found the prevalence of Atlantic rainforest since at least 7.6 ka BP, with no signal for a dry season until ca. 4.0 ka BP. After 4.0 ka BP taxa related to extreme moisture disappear. It is worth noting that the authors recognize a decrease in the marine influence ca. 2.5 ka BP, in accordance with the MRSL oscillation model of Martin and Suguio [11].

Santos et al. [12] studied sponge spicules, diatoms, and phytoliths from Maricá (RJ - Fig 3, number 91), finding a very dense scrub / tree vegetation between 8.5 ka BP and 6.5 ka BP, signalling a wetter climate, with a less humid period between 6.5 ka BP and 3.0 ka BP. At 3.0 ka BP a denser vegetation returns.

Coe et al. [13] analysed phytoliths and carbon isotopes from Cabo Frio (RJ - Fig 3, number 90) finding two zones in the soil profile. The bottom (Zone I) was dated from 10.2 ka BP to 6.2 ka BP showing a more humid environment than the present, while Zone II, showed a more xeric environment.

Ybert et al. [14] studied pollen and diatoms from a core at Cananéia (SP - Fig 3, number 83) suggesting the presence of a large paleo lagoon from 4.9 to 3.5 ka BP, suggesting a sea level 1.2 to 2 m higher than at present, which would explain the presence of a regional peak of shellmiddens dated between 5.7 and 3.3 ka BP, and 50 km away from the present coast. The lagoon desiccated ca. 3.5 ka BP, following the sea level lowering. The presence of rainforest was continuous since 5.0 ka BP in the area, with no major changes except to some episodes of higher humidity.

Sallun et al. [15] performed a multiproxy analysis of textural, mineralogical, geochemical and isotopic compositions of a paleo lagoon in Juréia (SP - Fig 3, number 82) detecting a clear signal of extreme variability in several elements marking the 8.2 ka RCC event, and another ca. 4.0 ka BP (which the authors relate to the 4.2 ka event, following Bond et al. [16]).

Silva et al. [17] studied pollen and carbon isotopes at Palhoça (SC - Fig 3, number 96) and detected three distinct intervals reflecting the lowering of sea level since 7.7 ka BP, with a strong shift in the delta ^13^C and C/N ratio values ca. 3.0 ka BP.

Barros et al. [18] studied ^13^C and ^18^O isotopes in mollusk shells at Patos Lagoon (RS - Fig 3, number 103) and detected a period of climatic oscillation ca. 3.2 ka BP, with a mean temperature increase of 6° C (from 17 to 23° C) and the increase of mixohaline species, suggesting instability in the water salinity.

Lopes et al. [19] studied diatoms from soils in the vicinities of Mirim Lake (RS - Fig 3, number 99) and found evidence of a drought ca. 4.0 ka BP, also probably linked to the 4.2 ka event.

References

1. Martin L, José M. L. Dominguez, Abilio C. S. P. Bittencourt. Fluctuating Holocene sea levels in eastern and southeastern Brazil: Evidence from multiple fossil and geometric indicators. J Coast Res. 2003;19(1):101–24. http://www.jstor.org/stable/4299151
2. Suguio K, Martin L, Bittencourt ACSP, Dominguez JML, Flexor J-M, Azevedo AE. Flutuações do nível relativo do mar durante o quaternário superior ao longo do litoral brasileiro e suas implicações na sedimentação costeira. Rev Bras Geociênc. 1985;273–86. http://dx.doi.org/10.25249/0375-7536.1985273286
3. Martin L, Bittencourt ACSP, Dominguez JML, Flexor JM, Suguio K. Oscillations or not oscillations, that is the question: comment on Angulo R.J. and Lessa G.C. “The Brazilian sea-level curves: a critical review emphasis on the curves from the Paranaguá and Cananéia regions.” Marine Geology. 1998;150:179–87
4. Angulo R, Lessa G, Souza M. A critical review of mid- to late-Holocene sea-level fluctuations on the eastern Brazilian coastline. Quat Sci Rev. 2006;25(5–6):486–506. <http://dx.doi.org/10.1016/j.quascirev.2005.03.008>
5. Angulo RJ, Lessa GC. The Brazilian sea-level curves: a critical review with emphasis on the curves from the Paranaguá and Cananéia regions. Mar Geol. 1997;140(1–2):141–66. <http://dx.doi.org/10.1016/s0025-3227(97)00015-7>
6. Lessa GC, Angulo RJ. Oscillations or not oscillations, that is the question-reply. Mar Geol. 1998;150:189.
7. Rubira FG, Perez Filho A. Regressão marinha que sucedeu o optimum climático holocênico. Rev Bras Geomorfol. 2021;22(3). http://dx.doi.org/10.20502/rbg.v22i3.1843
8. Santos-Fischer CB dos, Corrêa ICS, Weschenfelder J, Torgan LC, Stone JR. Paleoenvironmental insights into the Quaternary evolution of the southern Brazilian coast based on fossil and modern diatom assemblages. Palaeogeogr Palaeoclimatol Palaeoecol. 2016;446:108–24. <http://dx.doi.org/10.1016/j.palaeo.2016.01.018>
9. Gyllencreutz R, Mahiques MM, Alves DVP, Wainer IKC. Mid- to late-Holocene paleoceanographic changes on the southeastern Brazilian shelf based on grain size records. Holocene. 2010;20(6):863–75. <http://dx.doi.org/10.1177/0959683610365936>
10. Buso Junior AA, Ruiz Pessenda LC, de Oliveira PE, Fonseca Giannini PC, Lisboa Cohen MC, Volkmer-Ribeiro C, et al. Late Pleistocene and Holocene vegetation, climate dynamics, and amazonian taxa in the Atlantic Forest, Linhares, SE Brazil. Radiocarbon. 2013;55(3):1747–62. http://dx.doi.org/10.1017/s0033822200048669
11. Martin L, Suguio K. Variation of coastal dynamics during the last 7000 years recorded in beach-ridge plains associated with river mouths: example from the central Brazilian coast. Palaeogeogr Palaeoclimatol Palaeoecol. 1992;99(1–2):119–40. http://dx.doi.org/10.1016/0031-0182(92)90010-3
12. Santos CP, Gomes Coe HH, Carvalho da Silva AL, Osterrieth ML, Parolin M, Corrêa Luz Souza RC, et al. Paleoenvironmental evolution during the Holocene of the coastal plain of Maricá, Rio de Janeiro, Brazil, through silica biomineralizations. J South Am Earth Sci. 2023;121(104098):104098. http://dx.doi.org/10.1016/j.jsames.2022.104098
13. Coe HHG, Macario K, Gomes JG, Chueng KF, Oliveira F, Gomes PRS, et al. Understanding Holocene variations in the vegetation of Sao Joao River basin, southeastern coast of Brazil, using phytolith and carbon isotopic analyses. Palaeogeogr Palaeoclimatol Palaeoecol. 2014;415:59–68. <http://dx.doi.org/10.1016/j.palaeo.2014.01.009>
14. Ybert J-P, Bissa WM, Catharino ELM, Kutner M. Environmental and sea-level variations on the southeastern Brazilian coast during the Late Holocene with comments on prehistoric human occupation. Palaeogeogr Palaeoclimatol Palaeoecol. 2003;189(1–2):11–24. http://dx.doi.org/10.1016/s0031-0182(02)00590-4
15. Sallun AEM, Sallun Filho W, Suguio K, Babinski M, Gioia SMCL, Harlow BA, et al. Geochemical evidence of the 8.2 ka event and other Holocene environmental changes recorded in paleolagoon sediments, southeastern Brazil. Quat Res. 2012;77(1):31–43. http://dx.doi.org/10.1016/j.yqres.2011.09.007
16. Bond G, Showers W, Cheseby M, Lotti R, Almasi P, deMenocal P, et al. A pervasive millennial-scale cycle in North Atlantic Holocene and glacial climates. Science. 1997;278(5341):1257–66. <http://dx.doi.org/10.1126/science.278.5341.1257>
17. Silva WG, Souza PA, Garcia MJ, Carvalho M de A, Dillenburg SR, Cancelli RR, et al. Middle to Late Holocene paleoenvironmental changes in the coastal plain of southern Brazil. J South Am Earth Sci. 2021;111(103514):103514. http://dx.doi.org/10.1016/j.jsames.2021.103514
18. Barros CE, Baitelli R, Carvalho DDS, Dehnhardt BA, Bernardi CZ, Becker CD, et al. Aplicações de isótopos de oxigênio e carbono na reconstrução paleoambiental do interior da Lagoa dos Patos, RS, Brasil: um estudo de caso. Quat Environ Geosci. 2021;12(1). http://dx.doi.org/10.5380/abequa.v12i1.73396
19. Lopes RP, Silva de Souza M, Pereira JC, Raupp SV, Tatumi SH, Yee M, et al. Late Pleistocene-Holocene diatomites from the coastal plain of southern Brazil: Paleoenvironmental implications. Quat Int. 2021;598:38–55. <http://dx.doi.org/10.1016/j.quaint.2021.04.041>
